# An argument for abandoning the “allowed” and “forbidden” classification of electrocyclic reactions†

**DOI:** 10.1039/d4sc08748h

**Published:** 2025-02-19

**Authors:** Barry K. Carpenter

**Affiliations:** a School of Chemistry, Cardiff University CF10 3AT UK carpenterb1@cardiff.ac.uk

## Abstract

The division of electrocyclic reactions into “allowed” and “forbidden” classes carries the implication that reactions of the latter class are so energetically penalised that they will occur only if their “allowed” alternatives are rendered effectively impossible. The present work tests that assumption, using NEVPT2 and DFT calculations on a variety of cyclobutene ring openings and (*Z*)-1,3,5-hexatriene ring closures, and their benzannelated congeners. The results show the assumption to be incorrect. The potential energy differences between “forbidden” and “allowed” transition states are found to cover a wide range of values, with the smallest being less than half the classical barrier to internal rotation of ethane. It follows that planning a total synthesis on the presumption that electrocyclic reactions will always follow the “allowed” stereochemical course is an unreliable strategy because other commonly occurring factors, such as routine steric and electronic substituent effects, can easily outweigh the electronic penalty for following the nominally forbidden mechansim. A particular case involving a proposed synthetic route to a class of anticancer compounds is highlighted as an example.

## Abstract 2

The energy gaps between “forbidden” and “allowed” mechanisms of routine electrocyclic reactions can be negligibly small.

## Graphical abstract

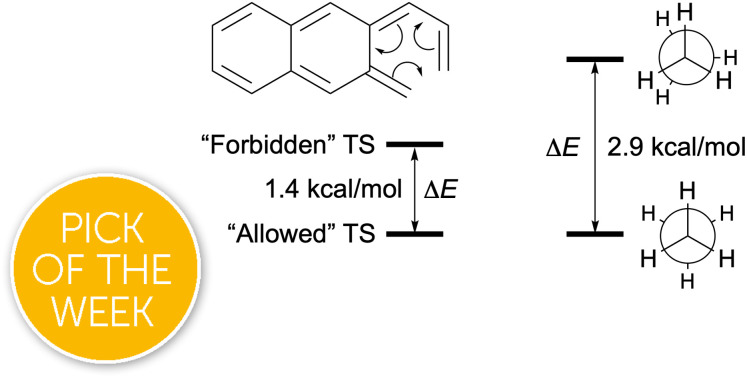

## 1. Introduction

Experimental and computational research over the last several decades has revealed that the neat partition of pericyclic reactions into “allowed” and “forbidden” classes can be an oversimplification. Particularly for sigmatropic reactions, the important role played by nonstatistical dynamics has blurred the boundary between the classes.^1–12^ However, electrocyclic reactions have seemed more clear-cut. To date, there have been no examples of such reactions in which dynamic effects have overridden the expectations from Transition State Theory. Nevertheless, the present work suggests that the binary classification of electrocyclic reactions is also misleading, but for reasons unrelated to dynamics. The implications of the present analysis are exemplified by the ring expansions of various dihydrocyclobuta-arenes that have been studied experimentally. Two cases are highlighted in which the final reaction stereochemistry is different from that predicted by the application of the Woodward–Hoffmann rules, although the reasons for the discrepancies are different in the two examples. One of these studies was a precursor to proposed total syntheses of anticancer compounds.

### 1.1 Selection rules for the stereochemistry of electrocyclic reactions

Electrocyclic reactions, by definition, involve the conversion of a π bond into to a σ bond or *vice versa*.^13,14^ This bonding change necessarily includes two internal rotations, which, if concerted, can occur with the same sense (conrotation) or the opposite sense (disrotation).^13,14^ The preferences for one stereochemistry or the other have been analysed by examination of the symmetries of frontier orbitals,^13,15,16^ by construction of correlation diagrams,^17,18^ and by the identification of the competing transition states as being aromatic or antiaromatic.^19–21^ No matter which model one chooses for the analysis, the outcome is invariably cast in binary terms: one stereochemistry is “allowed” and the other “forbidden.” The implication is that the underlying electronic factors – which are certainly real – are so powerful that, except in extreme circumstances, no other factors need be considered in making a stereochemical prediction for an electrocyclic reaction. The present work challenges the validity of that assumption.

The general strategy of this paper will be to compute energy differences between “forbidden” and “allowed” transition states. Such a task obviously depends on the identification of the mechanism of the “forbidden” process – an issue that requires resolution of a historical disagreement, which surprisingly seems not to have been addressed to date. In 1972, Berson and Salem published a communication^22^ in which they addressed the nature of “forbidden” pericyclic reactions. They wrote:

“By implication, the forbidden reaction would have a transition state with net antibonding character. It would be reasonable, therefore, to postulate that if extrasymmetric factors precluded the symmetry-allowed process, the system would shun the forbidden concerted pathway in favor of one in which the reactive sites tended to overlap as little as possible. This would have the important consequence that reactions proceeding by other than allowed pathways would tend to occur in two steps and stereorandomly. We wish to suggest that this conclusion is misleading and that there is a significant electronic factor favoring stereospecific, *concerted*, *forbidden* reactions”.

The electronic factor that Berson and Salem identified was the change in energy of the orbitals below the highest occupied molecular orbital (HOMO). They called these the subjacent orbitals.

Two years later, without citing the Berson and Salem publication, Dewar and Kirschner came to exactly the opposite conclusion,^23^ specifically for the case of electrocyclic reactions:

“During a ‘forbidden’ electrocyclic reaction, the system has to pass through a biradical intermediate corresponding to the HOMO/LUMO crossing. The most direct path from reactant to product leads through an intermediate biradical which is of very high energy, being antiaromatic. The most stable state of such a biradical will be one derived from the cyclic reactant by rotating one methylene group only, since any rotation of the second methylene will give rise to an unfavorable cyclic conjugation. The lowest point in the biradical barrier will therefore be that corresponding, in the symbols used above, to a 90°, 0° configuration (*i.e. ϕ*_1_ = 90°, *ϕ*_2_ = 0°). The 'best' reaction path will be one in which the reactant approaches this ideal structure as closely as possible before trying to cross the barrier. This can be achieved by rotating one methylene group only, the other retaining its original position (*ϕ*_2_ = 0°). As the first methylene rotates, the C–C σ-bond weakens and a new C–C π-bond forms. At some point, in the general vicinity of *ϕ*_1_ = 45°, the formation of the new π-bond will begin to outweigh weakening of the old σ-bond so the energy will begin to decrease with further increase in *ϕ*_1_. At this point any rotation of the second methylene will still increase the energy since interactions between it and the adjacent sp^2^ carbon are still antibonding. The transition state for the overall reaction will therefore correspond to the maximum (∼45°, 0°) since the path from this to the 90°, 0° configuration is downhill”.

The LUMO to which Dewar and Kirschner^23^ refer is the lowest unoccupied molecular orbital, and the torsion angles *ϕ*_1_ and *ϕ*_2_ are about the C=C bonds to the terminal methylenes in the ring-opened structure. Note, too, their explicit assumption that an antiaromatic transition state must be “…of very high energy…”.

It is clear that the Berson–Salem^22^ and Dewar–Kirschner analyses cannot both be correct under all circumstances. So, is one analysis always right and the other always wrong? Are they both wrong? Or are there some circumstances in which one is correct and different circumstances in which the other is correct? If so, what are those circumstances? The present work attempts to answer those questions, *en route* to its principal goal of computing energy differences between “allowed” and “forbidden” mechanisms.

### 1.2 Ring expansions of dihydrocyclobuta-arenes

**Scheme 1:**
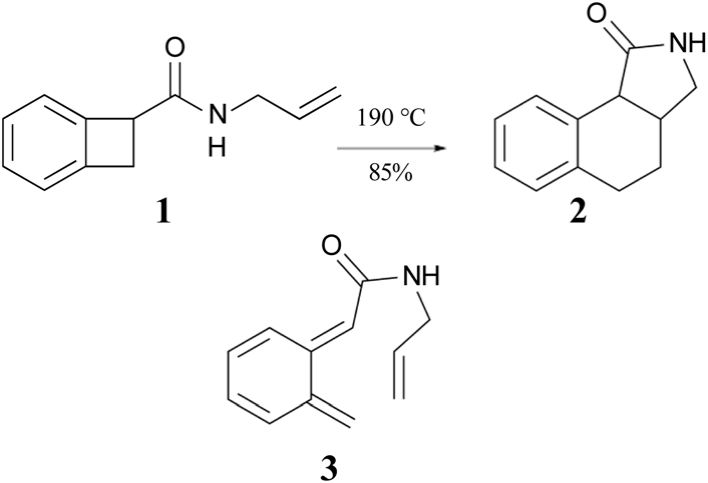
The first reported example of a benzocyclobutene ring expansion, presumed to occur by electrocyclic ring opening of 1 to 3 and subsequent intramolecular Diels–Alder cyclization of 3 to 2.

The ring opening and subsequent intramolecular cycloaddition of benzocyclobutenes‡‡Benzocyclobutene is not the correct IUPAC name for the parent hydrocarbon – it should be bicyclo[4.2.0]hexa-1,3,5-triene – but benzocyclobutene is commonly used throughout the organic chemistry literature, and so is adopted for this paper. was first reported by Oppolzer, who showed that heating compound 1 to 190 °C as a toluene solution in an autoclave resulted in an 85% isolated yield of compound 2 (Scheme 1).^24^ Although not isolated, compound 3 was presumed to be an intermediate in the reaction. This mechanism built on earlier studies involving intermolecular trapping of so-called orthoquinodimethanes.^25,26^ A study on the kinetics of a related compound also supported the mechanism.^27^ The sequence 1 → 3 → 2 would constitute a pair of pericyclic transformations – an electrocyclic reaction followed by a cycloaddition – to which the Woodward–Hoffmann (W–H) stereochemical selection rules^28^ would be expected to apply. The first reaction should be _π_6_s_ + _σ_2_a_ (*i.e.*, a conrotatory ring opening) and the second should be _π_8_s_ + _π_2_s_ (*i.e.*, a suprafacial cycloaddition). However, the reactant lacked the labels or substituents that could reveal whether the expected stereochemistry was observed.

Following the Oppolzer report, this general strategy for the synthesis of benzannelated compounds was widely adopted, notably by Kametani's group,^29–32^ but also by many others.^33–39^

In compound 3, the linker between diene and dienophile has a length of three atoms. The general strategy is known to work with other linker lengths.^36^ Of particular interest for the present work is the case of zero linker length, whereby the “dienophile” is directly connected to the diene. In such circumstances, the second step of the reaction is converted from a cycloaddition reaction to an electrocyclic ring closure. Again, this possibility has been recognised and demonstrated to work, with two independent research teams reporting such reactions in 1974, one being the group of Maitland Jones at Princeton^40^ and the other the group of Peter Sammes at Imperial College, London.^41^

**Scheme 2:**
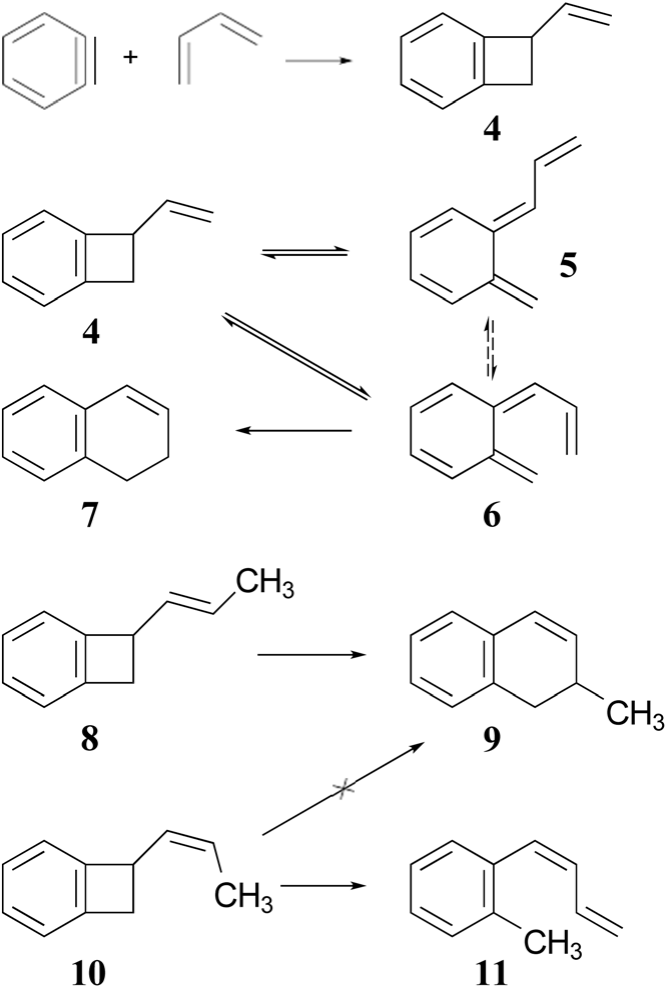
The preparation and rearrangement of alkenyl-benzocyclobutenes, reported by the group of Maitland Jones.^40^ The dashed reversible arrow linking species 5 and 6 constitutes a transformation investigated in the present work, but not suggested by the original authors.

The Jones group prepared the reactants by 2 + 2 addition of benzyne to various 1,3-dienes (Scheme 2). The rearrangements of the adducts were carried out at 190 °C. These authors recognised that the ring expansion could, in principle, be a single-step [1,3]-sigmatropic rearrangement, but they ruled out that mechanism when they discovered that the stereoisomeric reactants 8 and 10 underwent very different reactions. Compound 8 underwent the expected ring expansion, whereas 10 gave *o*-methylphenyl-1,3-butadiene – a reaction that could be understood as a [1,7]-hydrogen migration from the intermediate in the two-step mechanism.^40^

These authors also recognised that the initial ring opening of the benzocyclobutene, 4, could occur to give two stereoisomeric intermediates, 5 and 6, but that only 6 could undergo the second electrocyclic reaction. They expected that the activation barrier to form 5 would be lower than that to form 6, but that 5 would simply return to the reactant by the reverse of the electrocyclic reaction which formed it. They did not address the possibility that 5 and 6 might directly interconvert by internal rotation, but that possibility is explored in the present work.

**Scheme 3:**
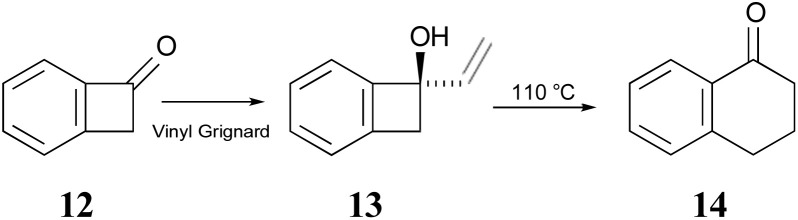
The prototype of the alkenyl-benzocyclobutene preparations and rearrangements reported by Sammes and coworkers.^41^

The Sammes group prepared compound 13 by addition of vinyl magnesium bromide to ketone 12, and then showed that it was quantitatively converted to α-tetralone (14) by refluxing in toluene (Scheme 3). Of some relevance to the discussion that follows is the fact that the temperature required for the rearrangement in this case was substantially lower than that reported by Oppolzer and Jones for their reactions.

In 1996, Wallace and coworkers expanded on the Sammes' chemistry in pursuit of synthetic routes to natural products, such as anticancer agents of the aureolic acid class (15).^42^ In their work, these researchers paid particular attention to the expected stereochemistry of the dual electrocyclic-reaction sequence. Specifically, the W–H rules would predict that compounds of type 17 should ring open by conrotation to give intermediates of type 18. As noted above, there are two possible conrotatory ring openings, but Wallace invoked Houk's “torquoselectivity” principle,^43^ to suggest that the preferred direction would rotate the hydroxyl group outwards, and hence the alkene substituent inwards, as required for the subsequent electrocyclic ring closure, for which the W–H rules predict disrotation. The torquoselectivity could explain why the hydroxy (or alkoxy) substituted benzocyclobutenes appear to rearrange at lower temperature than their hydrocarbon analogues.

In their experimental studies, the Wallace group did not probe the stereochemistry, but they did demonstrate that the ring expansion worked both for benzocyclobutenes, such as 20 → 21 and for dihydrocyclobuta[*b*]naphthalenes, such as 22 → 23. The latter reaction is important because it provides the prototype for synthesis of compounds such as 15.^42^ If one could demonstrate the stereoselectivity predicted in the sequence 16 → 19, it might seem all but self-evident that the same outcome would be found for the analogous dihydrocyclobuta[*b*]naphthalenes. Nevertheless, the present work suggests that this seemingly reasonable expectation is incorrect. Furthermore, deprotonation of alcohols such as 17 is also predicted to change the stereochemistry of the final ring closure.

**Scheme 4:**
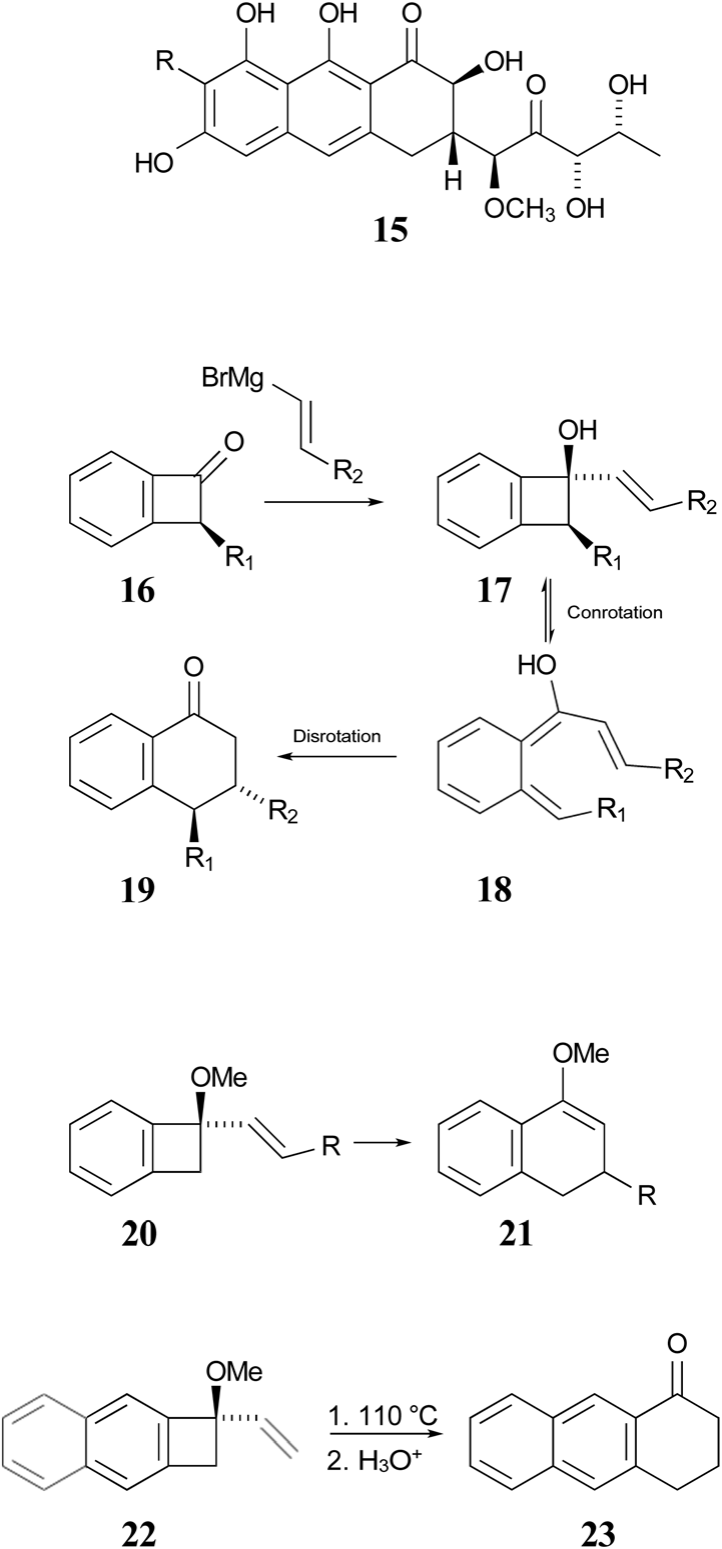
Potential application of dihydrocyclobuta-arene ring expansions to the synthesis of natural products, as suggested by Wallace and coworkers.^42^ The sequence 17 to 19 illustrates the stereochemical outcome expected from the W–H pericyclic selection rules.

## 2. Computational methods

The calculations have been carried out using the ORCA, GAMESS-US and Gaussian computational chemistry packages. Full references to these packages are provided in the ESI.† The calculations used the cc-pVTZ basis set,^44^ except where noted. Most of the results relied on complete active space self-consistent field (CASSCF) geometry optimisations, with the active spaces ranging from four electrons in four orbitals to fourteen electrons in fourteen orbitals, as required for the specific molecules under consideration. Dynamic correlation corrections were computed by NEVPT2(full, sc) single-point calculations from the CASSCF reference wave functions,^45–48^ where ”full” indicates that the frozen-core approximation was not employed and “sc” indicates that the strongly contracted version of NEVPT2 was chosen.^49^

For the molecules with methoxy substituents, density functional theory (DFT) was used instead of the NEVPT2//CASSCF model. There were three reasons for this. First, it was difficult to treat the oxygen lone pairs by CASSCF. Exclusion from the active space risked unrealistic localisation on oxygen for systems where the p-type lone pair could have interacted with a π-system. However, attempts to include the lone pairs in the active space were unsuccessful, with one or both lone-pair orbitals being consistently ejected from the active space during CASSCF wavefunction optimisation. Second, some of the molecules were too large to be treated at the NEVPT2 level with the available computational resources. Third, several of the calculations centred on the influence of nonbonded interactions. Even though these could be expected to be well described by the NEVPT2 single-point calculations, they might not be handled properly during the underlying CASSCF geometry optimisations, which have no better representation of dispersion effects than Hartree–Fock theory for orbitals not included in the active space. Three different functionals were tested: ωB97XD,^51^ PBE0-D3BJ^52,53^ and M06-2X.^54^ These calibration results are summarised in Table 1. As can be seen from the table, the functional providing the best results was M06-2X, and so it was selected for the remaining calculations in which DFT was required.

**Table 1:** Comparison of computational and experimental enthalpy changes (kcal mol^−1^). A is cyclobutene, B is s-*trans*-1,3-butadiene, C is benzocyclobutene and D is orthoquinodimethane. MUE is the mean, unsigned error

Enthalpy change	NEVPT2	ωB97X-D	PBE0-D3BJ	M06-2X	Expt
A → B Δ*H*°	−16.2	−9.8	−8.7	−11.1	−11.9^a^
A → B Δ*H*^‡^	30.4	36.3	35.0	34.9	31.4^b^
C → D Δ*H*°	10.2	17.2	17.8	14.9	10.6^c^
C → D Δ*H*^‡^	37.0	45.3	42.5	43.6	39.9^c^
MUE	2.2	4.8	4.2	3.1	

a Active thermochemical tables, v. TN1.202 (https://atct.anl.gov); accessed 23rd Oct. 2024.

b NIST Chemical Kinetics Database (https://kinetics.nist.gov/kinetics); accessed 23rd Oct. 2024.

c Ref. 50.

The pericyclic selection rules are based on classical potential energy differences between competing pathways. Hence, in the Results and discussion section, most of the focus is on ΔΔ*E*^‡^ – the difference in potential energy barriers between forbidden and allowed transition states. However, for comparison with experiment, calculations of ΔΔ*H*^‡^ or ΔΔ*G*^‡^ – respectively differences in enthalpy or Gibbs free energy barriers – are required. Such calculations require specification of a temperature. Where available, the experimental reaction temperature was utilised. When an experimental temperature was not available, one was selected that would give a unimolecular rate constant of 10^−5^ s^−1^, *i.e.* a half-life of about 19 h. Where Gibbs free-energy calculations were required, the entropy computations used the model described by Grimme for interpolating between low-frequency harmonic-oscillator and free-rotor partition functions.^55^ This is the default methodology for free-energy calculation in the ORCA package. However, unlike the default for that package, the calculations reported here used atomic masses for the most abundant single isotopes of each element, rather than natural-abundance weighted averages.

For some of the calculations reported in the Results and discussion section, it was found useful to calculate linear synchronous transit (LST) paths between pairs of structures. This has previously been carried out in Cartesian coordinates,^56^ with the steps along the path being equally spaced linear interpolations between the starting and ending structures. Unfortunately, that procedure typically provides poor descriptions of internal rotations, which invariably become combined with spurious bond-stretching motions. The problem can be corrected by constrained optimisation at each point,^56^ but that is impractical for CASSCF calculations because each calculation requires manual verification of the correct active space. An alternative approach is to carry out the LST procedure in bonded internal coordinates, a procedure which is here called BIC-LST. The “bonded” descriptor implies that the internal coordinates are chosen to correspond only to atoms connected by conventional covalent bonds. These can, of course, be identified manually, but that becomes tedious for large molecules, and so a procedure, executed in the form of a Python script, was developed to compute the BIC-LST automatically between two structures entered as Cartesian coordinates. The steps of the script are as follows:

(1) A connectivity list is generated for the first structure, based on the covalent radii and distances of the atoms.

(2) The connectivity list is subjected to a depth-first search (dfs). Each atom, in turn, is tried as the starting node for the dfs. The one leading to the longest connected path is selected.

(3) The result of the dfs is used to renumber the atoms.

(4) The renumbered Cartesian coordinates are used to generate a Z-matrix.

(5) Using the same renumbering, a Z-matrix is generated for the second structure.

(6) The two Z-matrices are interpolated in even steps.

(7) Each of the interpolated Z-matrices is converted back to Cartesian coordinates.

(8) The frames of Cartesian coordinates are back transformed to the original numbering scheme.

(9) The first frame is translated and rotated to the spectroscopic principal orientation and then, starting with the second frame, the Cartesian coordinates are reoriented for maximum overlap with the previous frame, using the Kabsch algorithm.^57^ This step does not change the energies of the frames, but it does make animation of the complete set of frames more easily visualised.

(10) A final combined output file is written and is used as input for a series of single-point energy calculations by the chosen electronic-structure program.

**Fig. 1:**
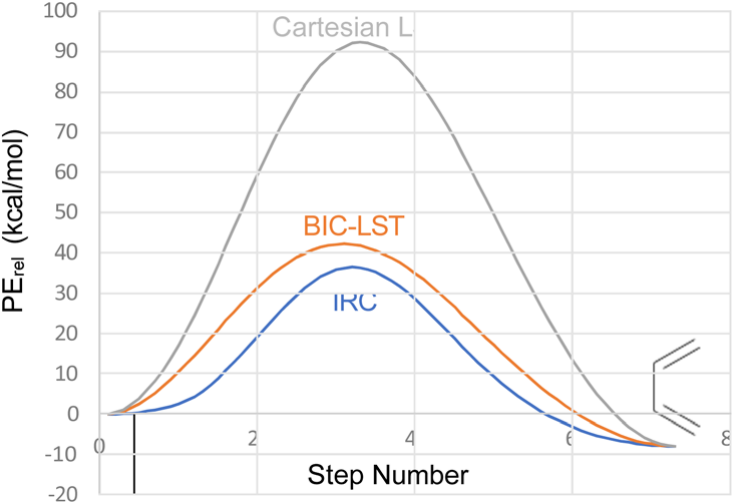
Comparison of Cartesian LST, BIC-LST and IRC for the conrotatory ring opening of cyclobutene at the M06-2X/cc-pVTZ level.

The advantage of the BIC-LST over the unoptimized Cartesian LST is summarised in Fig. 1, which compares both to the true intrinsic reaction coordinate (IRC) for the conrotatory ring opening of cyclobutene, calculated at the M06-2X/cc-pVTZ level.

## 3. Results and discussion

### 3.1 Nature of the transition states for “forbidden” electrocyclic reactions

**Scheme 5:**
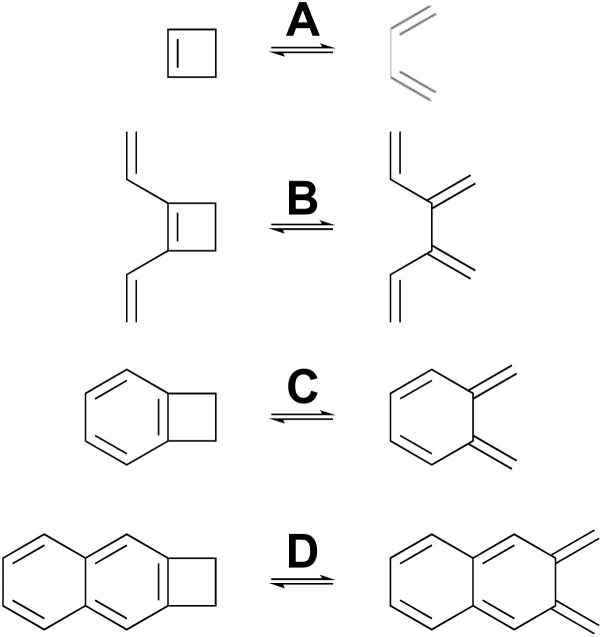
Ring openings of cyclobutene and its derivatives, for which computed activation barriers are reported in Table 2.

Many calculations have been carried out on the transition states of “forbidden” electrocyclic reactions, including some very recent reports.^58–65^ However, none of these studies has directly addressed the fundamental disagreement between Berson and Salem^22^ on the one hand and Dewar and Kirschner^23^ on the other, as outlined in the Introduction. That, then, is the focus of this section. NEVPT2(full, sc)//CASSCF(*n*,*n*)/cc-pVTZ calculations were carried out on four electrocyclic reactions for which the W–H rules would predict a conrotatory mechanism (Scheme 5). The parameter, *n*, specifying the number of electrons and also the number of orbitals in the active space is, in each case, twice the number of π-bonds in the ring-opened structure. The reactions were chosen so that all mechanisms for a particular ring opening would lead to the same product (in the absence of labels), thereby avoiding any thermodynamic bias on the transition-state energies. The reactions were also restricted to those for which the disrotatory transition structures would have exact *C*_s_ symmetry. This was necessary because three of the four stationary points of this kind turned out to be second-order saddles and it was only possible to optimise their geometries in exact *C*_s_ symmetry. Also calculated were the Dewar–Kirschner structures corresponding to 90° internal rotation of only one methylene. The results are summarized in Table 2. It should be noted that the conrotatory and disrotatory saddle points for reactions A and C have previously been calculated at the CASMP2//CASSCF(8,8)/6-31G(d,p) level by Sakai,^65^ who found an enthalpy gap of 19.6 kcal mol^−1^ for the former and 8.0 kcal mol^−1^ for the latter. The present calculations find somewhat larger values of 26.3 kcal mol^−1^ and 14.7 kcal mol^−1^, respectively (see ESI†), but the trend to a smaller gap upon benzannelation is clearly similar. Sakai provided a specific and sophisticated explanation for this trend. Here, it is presented in a wider context, as described below.

**Table 2:** NEVPT2(full, sc)//CASSCF(*n*,*n*)/cc-pVTZ potential energy and free energy barriers (kcal mol^−1^) for three mechanisms of the reactions depicted in Scheme 5. See text for explanation of the CASSCF parameter *n*

Reaction	Conrotation		Disrotation		Diradical	
	Δ*E*^‡^	Δ*G*^‡^	Δ*E*^‡^	Δ*G*^‡^	Δ*E*^‡^	Δ*G*^‡^
A	32.8	29.7	61.9^a^	54.8	45.7	39.2
B	34.8	31.9	64.2^a^	58.0	51.7	45.8
C	40.1	35.9	56.8^a^	50.3	47.7	41.2
D	43.7	38.9	54.7	47.8	46.9	40.5

a Second-order saddle point.

At first sight, the data in Table 2 look like a clear win for Dewar and Kirschner's diradical mechanism over Berson and Salem's forbidden concerted mechanism. The diradicals derived by 90° monorotation of one methylene are, in all four reactions, lower in energy than the saddle points for concerted disrotation. Furthermore, for three of the four reactions, the disrotation saddle points are of second order, meaning they are hilltops on the potential energy surface. However, there is more to say. It seems unambiguous that Berson and Salem got it wrong, but why? An obvious explanation is σ-strain. The disrotation saddle points have C–C–C bond angles somewhere between those of the cyclobutene and the ring-opened polyene. Since the cyclobutenes have considerable ring strain, the disrotation saddle points presumably have some fraction of that, too. Of course, the same is true for the conrotation saddle points, but they have the stabilising effect of transition state aromaticity, expected for an “allowed” reaction. By contrast, the monorotation diradicals have no bonding between the carbons that had been σ-bonded in the cyclobutenes, and so they have no significant angle strain. Because the Berson–Salem analysis concentrated only on the π-electrons in the reaction, they omitted this factor, which selectively destabilizes the disrotatory saddle point over the monorotatory one.

**Fig. 2:**
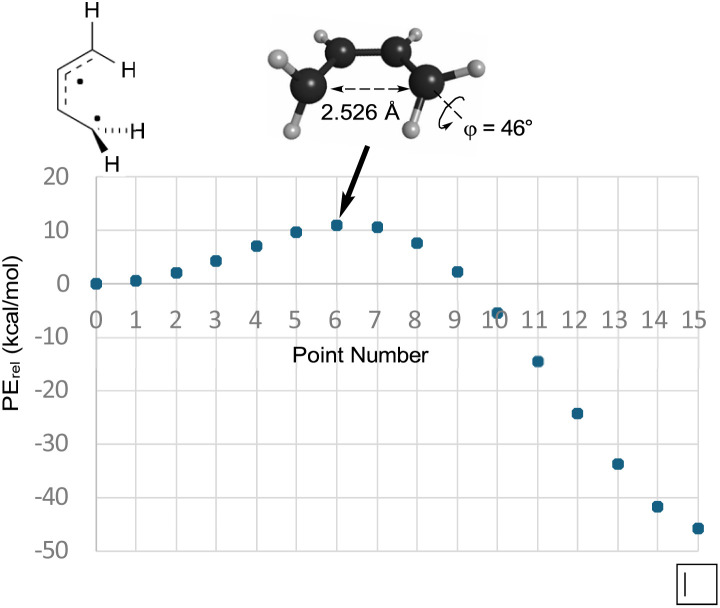
The BIC-LST path between the s-*cis*-butadiene internal rotation transition state and cyclobutene. Energies are relative potential energies at the NEVPT2(full, sc)//CASSCF(4,4)/c-pVTZ level.

So, if Berson and Salem got it wrong, did Dewar and Kirschner get it right? Well, no, not really. As can be seen in the text quoted in the introduction, Dewar and Kirschner expected the 0°, 90° diradicals to be intermediates, whereas they are first-order saddle points. Furthermore, they are not transition states for the “forbidden” electrocyclic reaction but are, instead, transition states for internal rotation of the ring-opened products, as revealed by IRC analysis. Still, one might be able to rescue something akin to the Dewar–Kirschner model if these saddle points could be directly connected to their cyclobutene analogues. A first-order saddle point along such a path could look very much like the one Dewar and Kirschner predicted. The only difference would be that it would lead not to a diradical intermediate but, instead, to a reaction path bifurcation. In order to see whether this was indeed the situation, a BIC-LST path (see the Computational methods section for description) was calculated for monorotatory cyclobutene ring opening. The result is shown in Fig. 2. The outcome was a path with a highest energy point about 11 kcal mol^−1^ above the diradical. The structure of this point was indeed very similar to the Dewar–Kirschner prediction for the “forbidden” transition state of cyclobutene ring opening. However, there is no geometry optimization involved in the BIC-LST calculation (except for the first and final points), and so an unconstrained transition state search was undertaken with the highest energy BIC-LST geometry as a starting point. This procedure, no matter which search algorithm was tried, invariably led to the conrotatory saddle point. Hence, at least at the level of theory used in the present work, there does not appear to be a direct path between cyclobutene and a disrotatory product *via* a conventional transition state. Instead, the lowest energy path to a disrotatory product is to follow the conrotatory ring opening and then to undergo 180° internal rotation about one of the terminal methylenes in the ring-opened product.

**Scheme 6:**
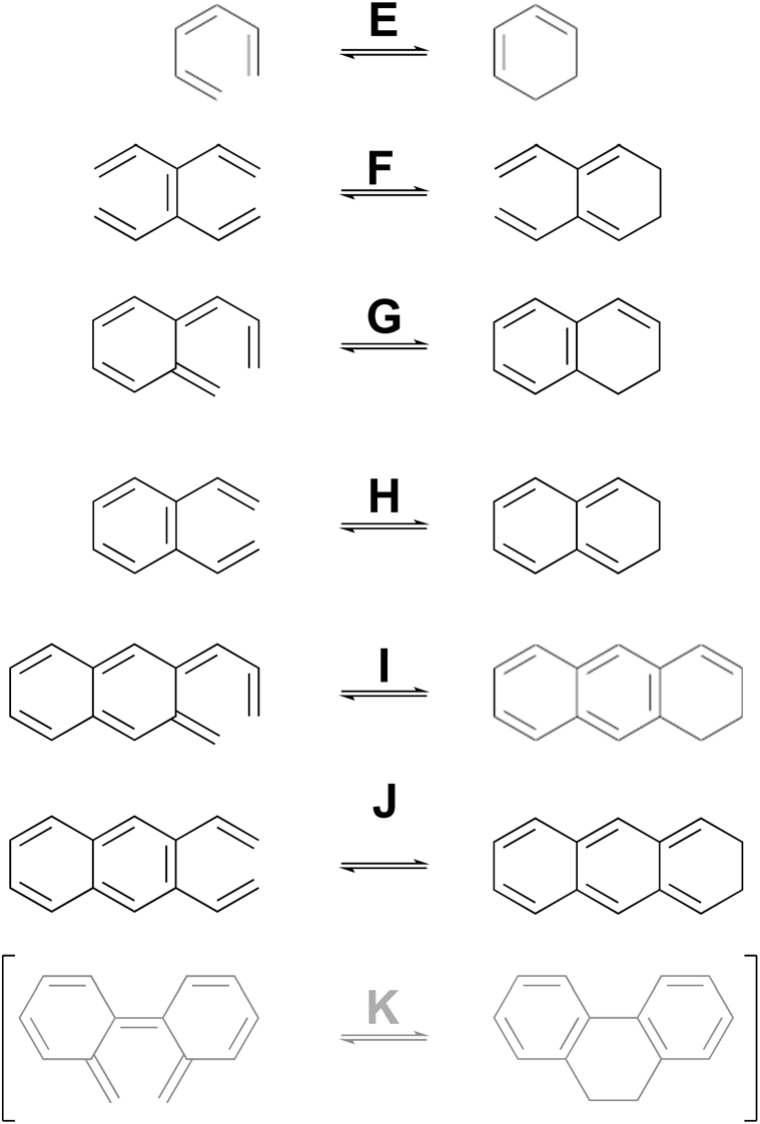
Ring closure reactions of (*Z*)-1,3,5-hexatriene and its derivatives, for which computed activation barriers are reported in Table 3. Reaction K was investigated but then excluded for reasons described in the text.

As a complement to the electrocyclic reactions described above, calculations were also carried out on the electrocyclic reaction of (*Z*)-1,3,5-hexatriene and its derivatives, summarised in Scheme 6. This time, it was not necessary to restrict the systems to those that would have strict *C*_s_ or *C*_2_ symmetry in the transition states, because the saddle points corresponding to concerted reactions (whether “allowed” or “forbidden”) all turned out to be of first order.

As can be seen from the data in Table 3, the story for this set of electrocyclic reactions is completely different from that for the cyclobutene derivatives. Now, the pericyclic saddle points for the “forbidden” conrotatory reactions are universally lower in energy than their monorotation, diradical counterparts.

**Table 3:** NEVPT2(full, sc)//CASSCF(*n*,*n*)/cc-pVTZ potential energy and free energy barriers(kcal mol^−1^) for various saddle points of the reactions depicted in Scheme 6. See text for explanation of the CASSCF parameter *n*

Reaction	Conrotation		Disrotation		Diradical	
	Δ*E*^‡^	Δ*G*^‡^	Δ*E*^‡^	Δ*G*^‡^	Δ*E*^‡^	Δ*G*^‡^
E	54.7	52.5	31.1	32.2	64.6^a^	61.5
F	43.6	41.2	25.3	25.5	54.6^a^	50.8
G	24.3	25.4	14.2	17.5	39.2^a^^b^	39.3
H	51.0	51.5	39.4	41.2	69.9^a^	67.5
I	10.4	9.5	9.0	9.3	27.6^a^^b^	26.2
J	46.9	48.3	44.8	46.3	69.4^a^	67.3
K	−0.8	−1.0	9.9	11.0	—c	—

a Second-order saddle point.

b There are two possible monorotations; only the lower energy one is shown here, but both are included in Table 4 and Fig. 5.

c The *C*_s_ diradical was too crowded, leading to rearrangement during attempted geometry optimisation.

**Fig. 3:**
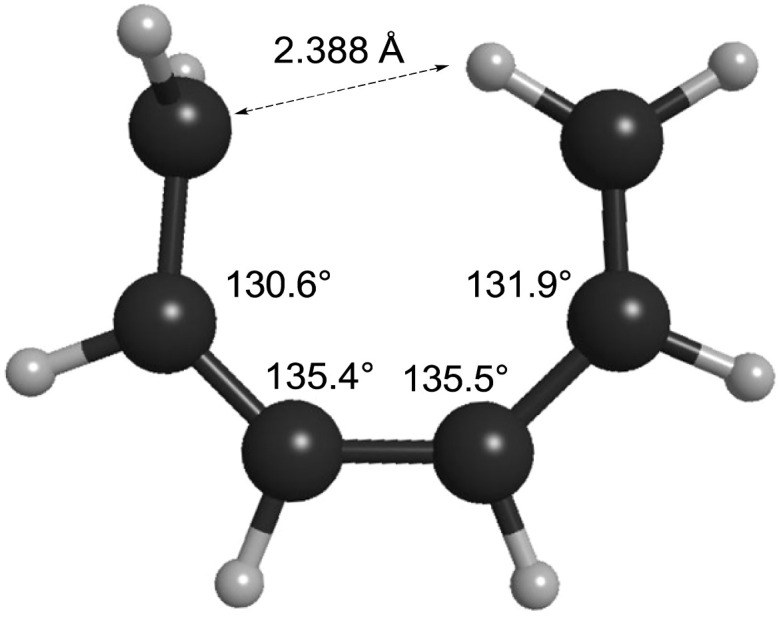
CASSCF(6,6)/cc-pVTZ optimised geometry of the Dewar–Kirschner *C*_s_ diradical derived from (*Z*)-1,3,5-hexatriene.

How can one understand the striking contrast between the two sets of electrocyclic reactions? The answer has to do with the omissions from the competing theoretical models. As described earlier, Berson and Salem had omitted σ-strain from their model, but so had Dewar and Kirshner. In the case of the cyclobutene ring openings, the omission selectively disadvantaged the Berson–Salem model, but for the hexatriene ring closures it is the other way round. The “forbidden” conrotatory saddle points have lower σ-strain than their monorotatory counterparts. The source of the strain in the latter can be seen in Fig. 3, which depicts the CASSCF(6,6)/cc-pVTZ optimised geometry of the parent *C*_s_ diradical.

One can see that one hydrogen of the in-plane methylene would be very close to the carbon of the out-of-plane methylene if the other carbons had normal bond angles to their neighbours. Relief of that nonbonded repulsion comes from expanding the C–C–C bond angles, but that costs σ-strain energy. In the case of reaction K, the corresponding diradical is so strained that it could not be geometry optimised without a hydrogen migration occurring.

There is a second omission from the Dewar–Kirshner model. As already highlighted, they assumed that “forbidden” pericyclic transition states would be fatally destabilized by their antiaromaticity. As described in more detail in the next section, that assumption was incorrect. The source of their error was omission of precisely the subjacent orbital effects that Berson and Salem had identified.^22^ Although the HOMO invariably rises in energy upon closing an antiaromatic ring, as Dewar and Kirschner expected,^23^ the other occupied orbitals can decrease in energy, and may do so by an amount that exceeds the rise of the HOMO.

It should be mentioned that this discussion of omissions from the Dewar–Kirschner model applies specifically to their quoted qualitative model. They carried out MINDO/3 semiempirical calculations, which seemed to support their qualitative picture, and which should have accounted for the factors claimed to be omissions in the present discussion.^23^ However, the MINDO/3 method had a flaw that applied specifically to singlet diradicals. In order to describe such species, one needs at least a two-configuration wave function. In MINDO/3 this was accomplished by a 2 × 2 configuration-interaction (CI) calculation. However, MINDO/3 included an empirical correction for electron correlation, which arose by adjustment of its empirical parameters to fit experimental heats of formation. When an explicit addition of an electron-correlation effect was included, as in the CI calculation, it corresponded to a “double counting” of correlation, with the result that singlet diradicals were systematically over stabilised with respect to their closed-shell isomers. The equivalent error in *ab initio* theory would be to compare the energy from a CASSCF calculation on a singlet diradical with that from a simple RHF calculation on its closed-shell isomer, instead of using CASSCF with a consistent active space throughout.

In summary, then, one can conclude that the Dewar and Kirschner model for “forbidden” electrocyclic reactions is universally incorrect, while the Berson and Salem model is correct for hexatriene ring closures but incorrect for cyclobutene ring openings, probably because of their omission of σ-strain effects.

### 3.2 The argument for abandoning the “allowed” and “forbidden” classification of electrocyclic reactions

The results presented here constitute the principal point of this paper. The argument begins with a seemingly foolish step, which is to find out what simple Hückel theory^66–68^ has to say about the reactions A–J in Schemes 5 and 6 (for reasons described below, reaction K is omitted). The reader may well question the sanity of this step because it seems impossible to believe that a model as crude as simple Hückel theory could have anything useful to contribute beyond what the NEVPT2 calculations have already revealed. However, it is the very simplicity of Hückel theory that is its strength for the present purposes. The Hückel model ascribes all effects to just one thing – the one-electron part of the Hamiltonian for the π-electrons. In other words, there are no σ-electron effects, no explicit electron–electron repulsions and no electron correlation effects. Those omissions sound as if they would be disastrous for the problem at hand, and indeed they are if one considers only reactions of individual molecules. However, as shown below, their omission is actually helpful when one compares reactions of similar type. Critically, all the historical descriptions of what makes an electrocyclic reaction “allowed” or “forbidden” should be contained within the Hückel description. The Berson–Salem subjacent orbital effects will also be included. Hence, the real exercise being undertaken here is a separation of the essence of electrocyclic reaction selection rules from all other factors that might influence the reaction barrier.

**Fig. 4:**
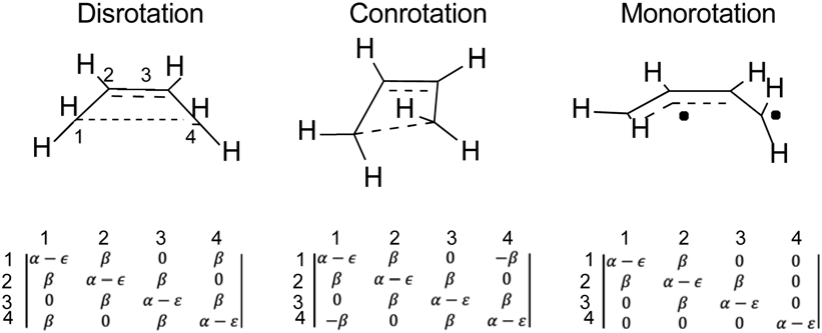
Hückel secular determinants corresponding to the structures above them. The parameters α and β are, respectively, the Hückel coulomb and resonance integrals.

An illustration of how the Hückel calculations are carried out is presented in Fig. 4, where the Hückel secular determinants are written down for each of the three types of ring opening of cyclobutene: disrotation, conrotation and monorotation. These determinants are set equal to zero and expanded to solve for the energy levels, ε, in terms of the Hückel coulomb and resonance integrals α and β, respectively. Alternatively, and more practically for machine computation, the matrices corresponding to each determinant are diagonalised to find their eigenvalues, which are then the energy levels. The total π-electron energy is taken to be simply the sum of the energies of the orbitals, weighted by their occupation numbers.

The discussion in Section 3.1 leads to the recognition that σ-strain effects are very different between cyclobutene-type and hexatriene-type electrocyclic reactions, and also between the diradicals corresponding to monorotation and either of the pericyclic saddle points. This recognition leads naturally to a set of empirical linear equations that should describe energy differences between reaction types for the reactions A–J in Schemes 5 and 6:

1 (Δ*E*^‡^_DIS_ − Δ*E*^‡^_CON_)_A–D_ = *m*(*E*^F^_4_ − *E*^A^_4_) + *c*_1_

2 (Δ*E*^‡^_CON_ − Δ*E*^‡^_DIS_)_E–J_ = *m*(*E*^F^_6_ − *E*^A^_6_) + *c*_2_

3 (Δ*E*^‡^_DIS_ − Δ*E*^‡^_DR_)_A–D_ = *m*(*E*^F^_4_ − *E*^D^_4_) + *c*_3_

4 (Δ*E*^‡^_CON_ − Δ*E*^‡^_DR_)_E–J_ = *m*(*E*^F^_6_ − *E*^D^_6_) + *c*_4_

In these equations, the terms on the left-hand sides refer to NEVPT2 potential energy differences, with the subscripts to the parentheses specifying the reactions to which they apply. On the right-hand sides, the Hückel energy terms in parentheses have a superscript that specifies whether they are for the “forbidden” or “allowed” pericyclic transition states, or for the diradical corresponding to monorotation. The subscripts specify whether these are cyclobutene-type ring openings (reactions A–D) or hexatriene-type ring closures (reactions E–J). The parameters *m* and *c*_1_–*c*_4_ are empirical constants with values to be determined by least-squares fitting. All the missing factors in Hückel theory, outlined above, are lumped into these constants. The relevant data are summarised in Table 4.

**Table 4:** Comparison of simple Hückel and NEVPT2 results for the reactions A–J depicted in Schemes 5 and 6. The meanings of the Hückel *E* parameters are described in the text

	Hückel (β units)	NEVPT2 (kcal mol^−1^)	Hückel (β units)	NEVPT2 (kcal mol^−1^)
	***E*** ^**F**^_**4**_ **− *E*** ^**A**^_**4**_	**Δ*E*** ^**‡**^_**DIS**_ **− Δ*E*** ^**‡**^_**CON**_	***E*** ^**F**^_**4**_ **− *E*** ^**D**^_**4**_	**Δ*E*** ^**‡**^_**DIS**_ **− Δ*E*** ^**‡**^_**DR**_
A	−1.657	29.18	1.172	16.23
B	−1.261	29.39	1.333	12.49
C	−0.914	16.72	1.661	9.07
D	−0.714	10.98	1.773	7.78

	***E*** ^**F**^_**6**_ **− *E*** ^**A**^_**6**_	**Δ*E*** ^**‡**^_**CON**_ **− Δ*E*** ^**‡**^_**DIS**_	***E*** ^**F**^_**6**_ **− *E*** ^**D**^_**6**_	**Δ*E*** ^**‡**^_**CON**_ **− Δ*E*** ^**‡**^_**DR**_
E	−1.072	23.54	1.464	−9.94
F	−0.855	18.31	1.585	−11.02
G	−0.585	10.06	1.714	−14.92
G^a^	—	—	1.895	−21.35
H	−0.585	10.37	1.895	−18.98
I	−0.430	1.36	1.801	−17.27
I^a^	—	—	1.993	−23.61
J	−0.430	2.09	1.993	−22.52

a Results for the higher energy of the two diradicals.

Fitting the empirical constants in eqn (1)–(4) to these data afforded the following results:*m* = −23.6 ± 1.4 kcal mol^−1^ β^−1^*c*_1_ = −5.2 ± 7.5 kcal mol^−1^*c*_2_ = −4.6 ± 3.7 kcal mol^−1^*c*_3_ = 46.4 ± 2.5 kcal mol^−1^*c*_4_ = 24.8 ± 3.6 kcal mol^−1^

**Fig. 5:**
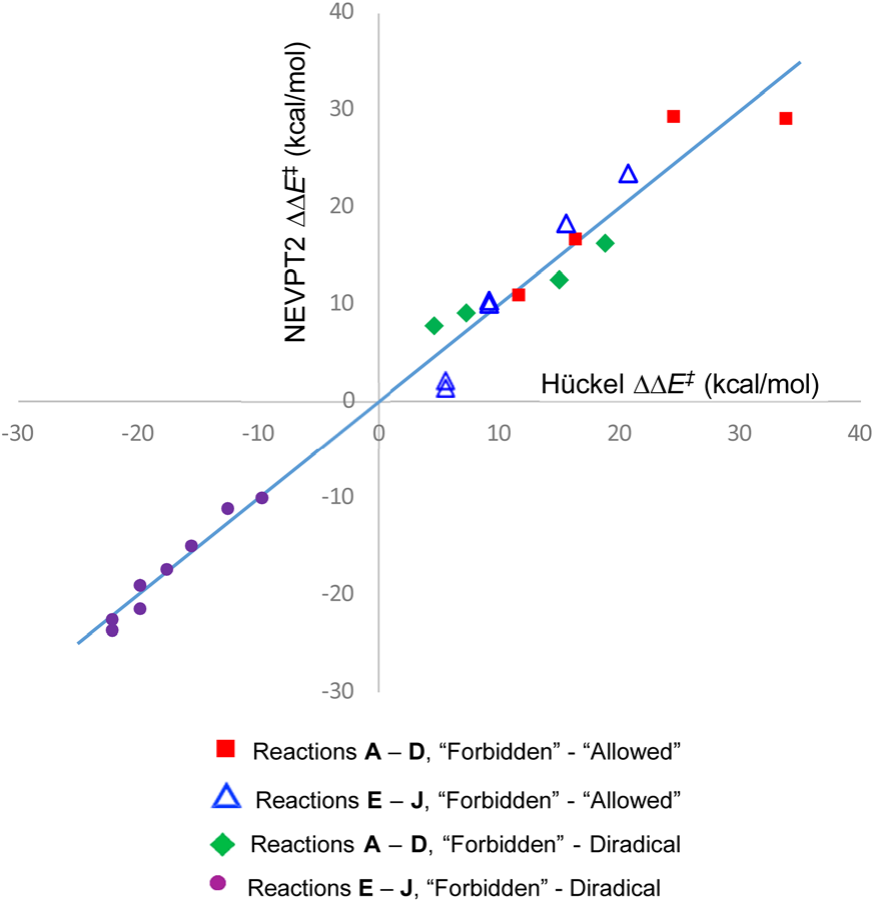
Graphical depiction of eqn (1)–(4), using best-fit values for the parameters *m*, *c*_1_–*c*_4_. The line has unit gradient and zero intercept and is included just to guide the eye.

The quality of the fit is depicted in Fig. 5. The value of *m* from the fit can be taken as a best estimate of the magnitude of the Hückel resonance integral in kcal mol^−1^. It turns out to be very similar to the value from a previous estimate.^69^

**Fig. 6:**
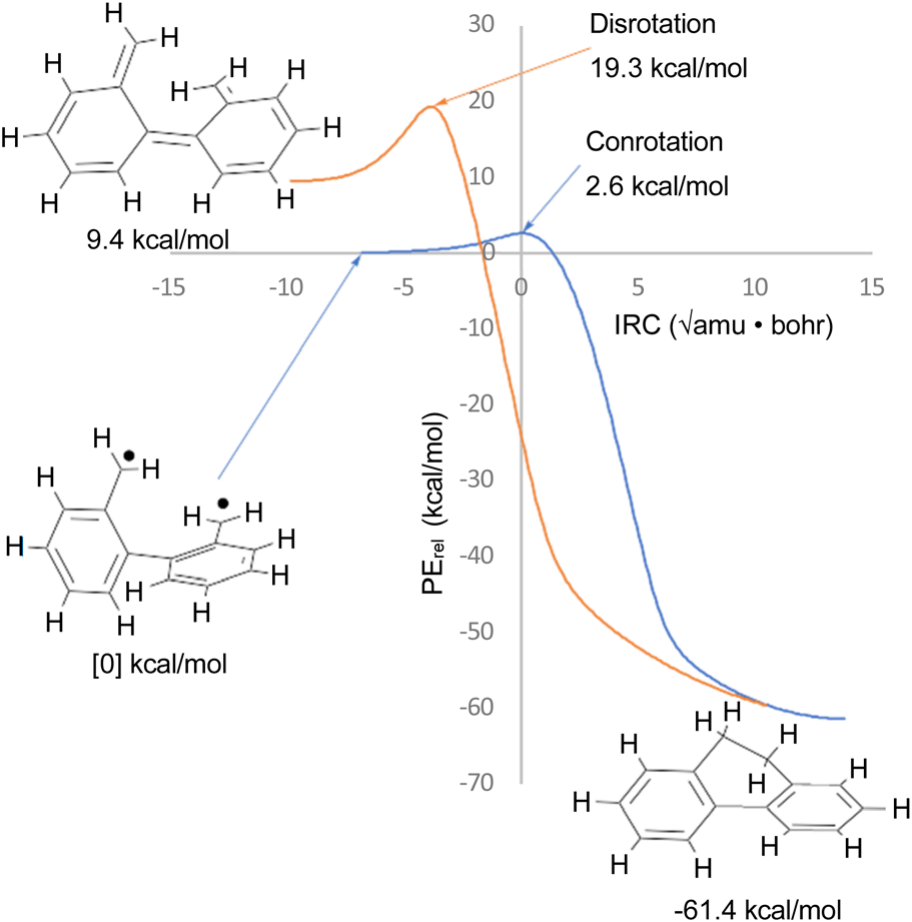
CASSCF(14,14)/cc-pVTZ intrinsic reaction coordinates for reaction K in Scheme 6. The 9,10-dihydrophenanthrene product is connected to two different electronic states of the reactant by the disrotatory and conrotatory mechanisms. The energies are different from those in Table 3, which included the NEVPT2 correction.

The justification for excluding reaction K from the analysis is that CASSCF(14,14)/cc-pVTZ IRC calculations revealed that the disrotatory and conrotatory ring openings of 9,10-dihydrophenanthrene (*i.e.* the reverse of reaction K) lead to different electronic states of the ring-opened structure. This is illustrated in Fig. 6. Apparently, the steric clash between the methylenes is sufficiently severe that the system prefers a bibenzyl diradical state for the ring-opened molecule. In all likelihood, there exists a conical intersection between the closed-shell and open-shell singlet states in the vicinity of the transition states, but it was deemed to be beyond the scope of this paper to search for it.

One message to be taken away from Fig. 5 is the failure of the Dewar–Kirshner model for hexatriene ring closures. Had their model been universally valid, the purple circles and green diamonds in Fig. 5 would all have been in the upper right quadrant of the plot. The green diamonds are, but the purple circles clearly are not. The reasons for this were discussed in Section 3.1.

But the most important message, and the reason for the title of this paper, is the distribution of blue triangles and red squares in Fig. 5. As outlined in the introduction, the binary “allowed” *vs.* “forbidden” classification of electrocyclic reactions encourages one to believe that the energy difference between the two will always be large enough to outweigh any other effects, except in extreme circumstances. Fig. 5 shows that this is simply not an accurate approximation. Instead, there is a range of values from 1.4 to 29.3 kcal mol^−1^. The former value is less than half the height of the classical energy barrier to internal rotation in ethane.^70^

On reflection, this range of values for the energy gap between “forbidden” and “allowed” pericyclic mechanisms shouldn't come as a surprise. Since the early days of attempts to analyse pericyclic reactions, it has been recognised that the concepts of transition state aromaticity or antiaromaticity provide useful insights into the energies of competing mechanisms.^21^ Sakai has more recently expanded that discussion, using modern theoretical methods.^71^ But for ground state molecules, aromatic stabilisation or antiaromatic destabilization are not single-valued quantities. On the aromatic side, the 9,10 bond in phenanthrene behaves much more like an alkene than part of a benzene ring.^72^ On the antiaromatic side, cyclobutadiene has but a fleeting existence in solution, but its benzannelated derivative, biphenylene, is an isolable, crystalline solid with a well-defined melting point.^73^ The present work is merely emphasising that transition state aromaticity and antiaromaticity have the same variability as their ground state counterparts. But there are real-world consequences from that variability, as Section 3.4 seeks to demonstrate.

### 3.3 A comment on transition-state polarity effects

**Scheme 7:**
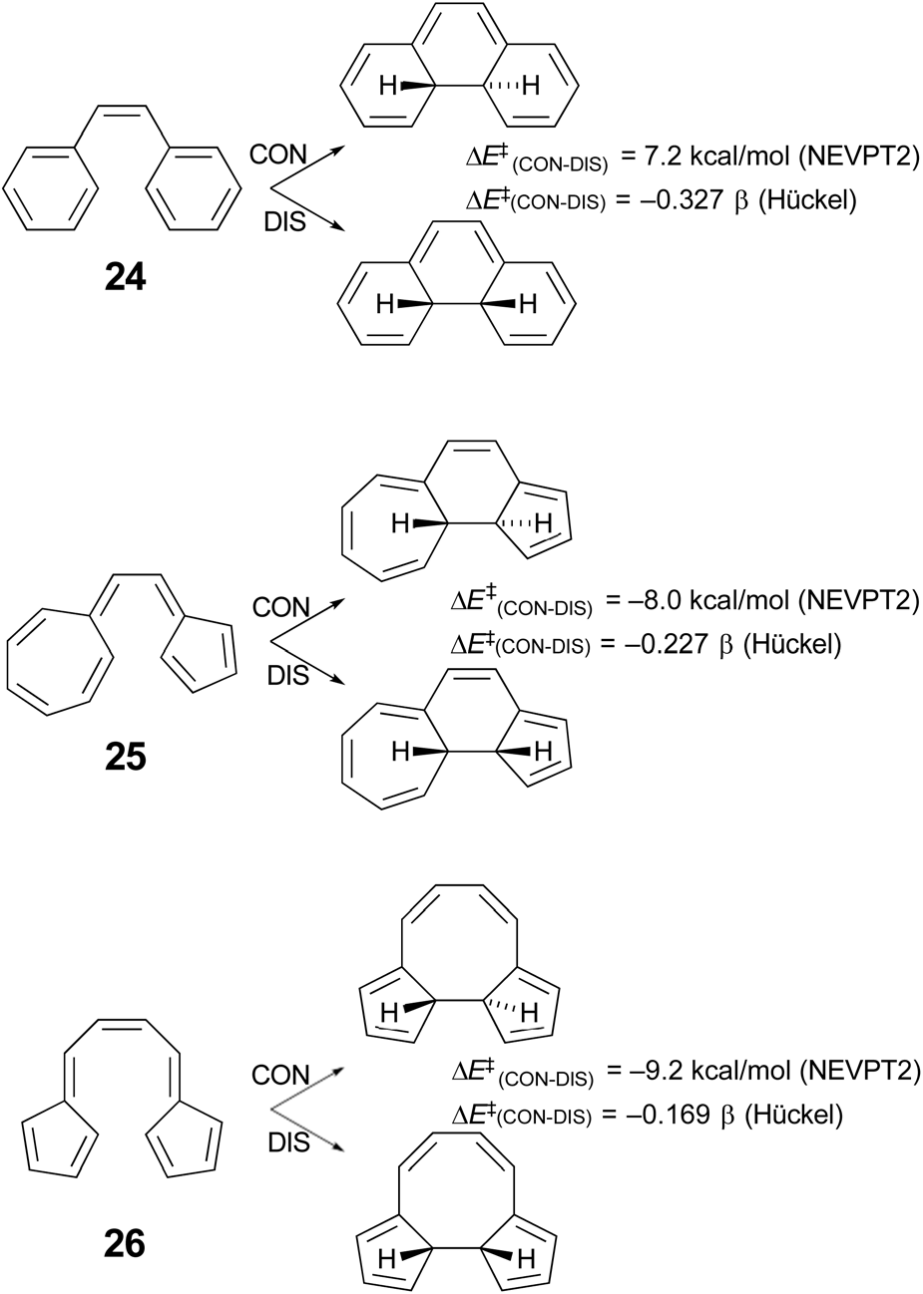
Results of simple Hückel and NEVPT2(full, sc)//CASSCF(14,14)/cc-pVTZ) calculations on three 14 π-electron electrocyclic ring closures.

Houk and coworkers^74^ have studied the cyclisation of hydrocarbon 25 (Scheme 7), which Prinzbach had earlier shown to close by the “forbidden” conrotatory pathway.^75^ Houk's DFT calculations were consistent with the experimental results, showing a Gibbs free energy difference of 11.2 kcal mol^−1^ between the transition states for disrotatory and conrotatory closure. Their analysis of why the reaction followed the supposedly “forbidden” pathway led them to conclude that it was the highly dipolar character of the transition state that was responsible. This was subsequently identified more generally as one of the characteristics of a pericyclic transition state that should be considered when assessing the applicability of the W–H rules.^64^ However, it seemed worth considering the possibility that the variable transition-state aromaticity effects addressed here might also play a role, because the difference in Hückel π-electron energies between conrotatory and disrotatory transition states for ring closure of compound 25 is only −0.227 β, which is smaller in absolute magnitude than the gaps found for any of the reactions A–J in Table 4. The smallest of these, reaction I, with a Hückel energy gap of −0.430 β, had an NEVPT2 potential energy difference between conrotatory and disrotatory pathways of only 1.36 kcal mol^−1^ and a free energy difference that was actually slightly in favour of conrotation, by 0.12 kcal mol^−1^. It seemed conceivable, therefore, that the much narrower Hückel energy gap for compound 25 could imply a stronger preference for the conrotatory mechanism.

Unfortunately, the 14 π-electron ring closure of compound 25 cannot be quantitatively compared with any of the reactions A–J, because it does not belong to either the of the classes of cyclobutene ring opening or hexatriene ring closure considered in Schemes 5 and 6. However, one can carry out a numerical experiment to see whether polarity is the sole factor leading to the “forbidden” ring closure of compound 25. The idea is to compare the difference in calculated barriers for conrotatory and disrotatory closures of three isomers – 24, 25 and 26 (Scheme 7).

In each case, the electrocyclic reaction has 14 π electrons and so would be predicted to be disrotatory by the W–H rules. As just discussed, this expectation is not met experimentally or by DFT computation for compound 25. If the polarity of the transition state were the sole explanation for this outcome, one would predict preferred disrotation for the ring closures of both 24 and 26. Houk and coworkers did carry out DFT calculations on the ring closure of 24 and found that, at the UωB97X-D/6-311++G(d,p)//UωB97X-D/6-31-G(d) level, the preferred route was indeed disrotatory, but the energy gap was small.^74^ Those results have been qualitatively confirmed by NEVPT2 calculations in the present work. But of more interest is the result for the ring closure of compound 26, which is found to have an even narrower Hückel energy gap than that for compound 25. If transition-state polarity were the principal factor determining the stereochemistry of the reaction, the small Hückel energy gap should be irrelevant, whereas the variable aromaticity effects considered here could lead one to expect an even stronger preference for conrotation for compound 26 over compound 25. The NEVPT2 results clearly support the latter outcome. It would appear that the ring closure of compound 26 is, in the language of ref. 64, a W–H violation of the second order. Compound 26 has been synthesised, again by Prinzbach.^76,77^ He clearly anticipated that it would ring close by the “forbidden” conrotation. Unfortunately, the product was not stable to the reaction conditions and so an experimental check of his expectations and of the present calculations is not possible.

### 3.4 Ring expansions of dihydrocyclobuta-arenes

**Fig. 7:**
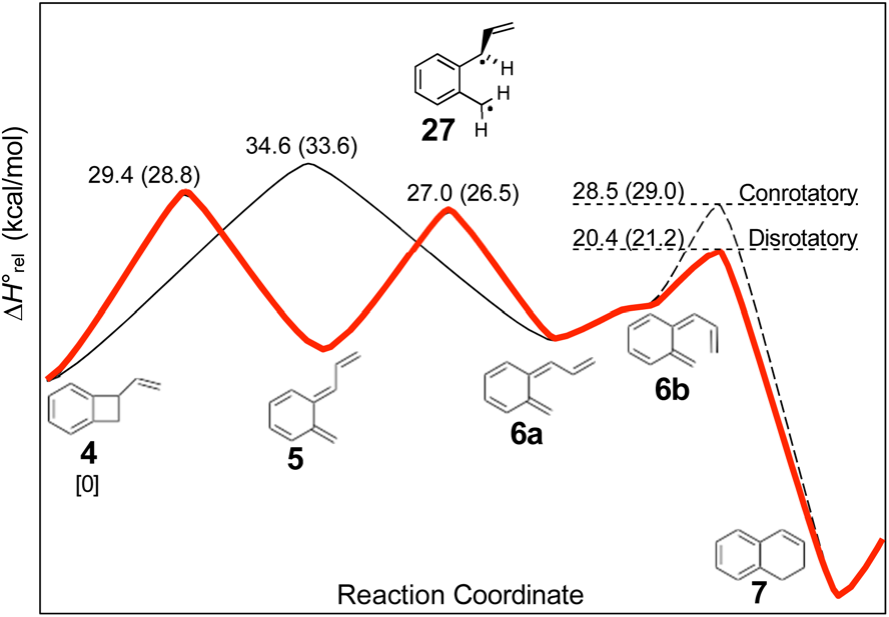
NEVPT2(full, sc)//CASSCF(10,10)/cc-pVTZ enthalpy profile for the ring expansion of vinyl-benzocyclobutene to 1,2-dihydro-naphthalene. The minimum energy path is shown in red. Numbers in parentheses are relative Gibbs free energies at the reaction temperature of 190 °C.

The experimental study on the ring expansion of vinyl-benzocyclobutene (4) to 1,2-dihydronaphthalene (7) by Maitland Jones's group^40^ was presented in the Introduction. The computed NEVPT2 enthalpy profile for the reaction is shown in Fig. 7.

**Scheme 8:**
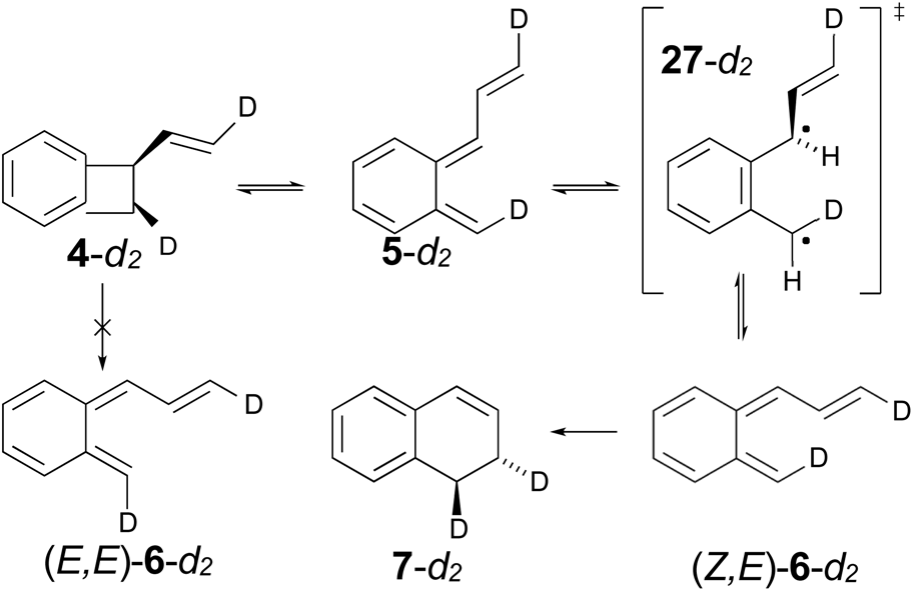
Predicted stereochemistry for the ring expansion of labelled compound 4-*d*_2_. The stereochemistry of 7-*d*_2_ is not the one predicted by the selection rules for electrocyclic reactions.

The authors had anticipated that the “outward” ring opening of 4 to 5 would face a lower barrier than the “inward” opening to 6, and the calculations support that expectation, with the barrier to form 5 being lower by 5.2 kcal mol^−1^ in enthalpy and 4.8 kcal mol^−1^ in free energy at the reaction temperature of 190 °C. Only stereoisomer 6 is capable of undergoing the second electrocyclic reaction to give the final product, 7. The authors had expected that 5 would be formed reversibly and would simply return to 4 by the reverse of the conrotatory electrocyclic reaction that had created it. However, that is not what the calculations reveal. They find that the direct interconversion of 5 and 6a by internal rotation has a lower barrier than the reversion to 4 by 2.4 kcal mol^−1^ in enthalpy and 2.3 kcal mol^−1^ in free energy. Furthermore, the internal rotation directly generates compound 6a, whereas the reverse electrocyclic reaction only regenerates 4, which then faces a barrier of 34.6 kcal mol^−1^ (33.6 kcal mol^−1^ in free energy) to formation of 6a. At 190 °C the difference of 7.1 kcal mol^−1^ in free energy between the two mechanisms corresponds to a rate-constant ratio of more than 2000 in favour of internal rotation. This has an important consequence for the overall reaction stereochemistry, as detailed in Scheme 8. The transition state for the internal rotation is the Dewar–Kirschner diradical 27. The discussion in Section 3.1 would lead one to expect that this diradical would be lower in energy than the second-order saddle point for disrotatory ring opening of 4, but the present calculations find that it is also lower in energy than the first-order saddle point for “outward” conrotatory ring opening of 4. The reason for the unusual stability of the diradical in this case is obvious: both of the nominally unpaired electrons are resonantly stabilised in 27, whereas only one had been in the diradicals discussed in Section 3.1.

Compound 6 must achieve conformation 6b (Fig. 7) before it can undergo the second electrocyclic reaction. The CASSCF geometry optimisation did not find 6b to be a local minimum. Instead, it shows up just as a “shoulder” on the IRC for the disrotatory ring closure. The calculations find the “allowed” disrotatory ring closure to have a lower barrier than the conrotatory one by some 8 kcal mol^−1^. These findings can now be combined to make a stereochemical prediction for the ring expansion of the as-yet-hypothetical labelled compound 4-*d*_2_. It is shown in Scheme 8.

The lower activation free energy for the outward conrotation of 4-*d*_2_ over its “inward” counterpart means that the first-formed ring-opened product will be 5-*d*_2_. This is predicted to isomerise to the stereoisomer that can undergo the final ring closure not by reverting to the reactant, as the authors had guessed, but by undergoing internal rotation *via* a diradical transition state. This means that the precursor to the final electrocyclic reaction is (*Z*,*E*)-6-*d*_2_, rather than (*E*,*E*)-6-*d*_2_. The final electrocyclic reaction is predicted to occur by the “allowed” disrotatory mechanism, affording the final product 7-*d*_2_.

The authors' mechanism would have led them to predict a *cis* relationship between the labels in the final product. The fact that the present calculations predict a *trans* relationship is not a failure of the pericyclic selection rules but occurs because of the unanticipated intervention of the Dewar–Kirschner diradical. The story is different for the next set of reactions, which are treated by DFT rather than NEVPT2, for reasons outlined in the Computational methods section.

**Fig. 8:**
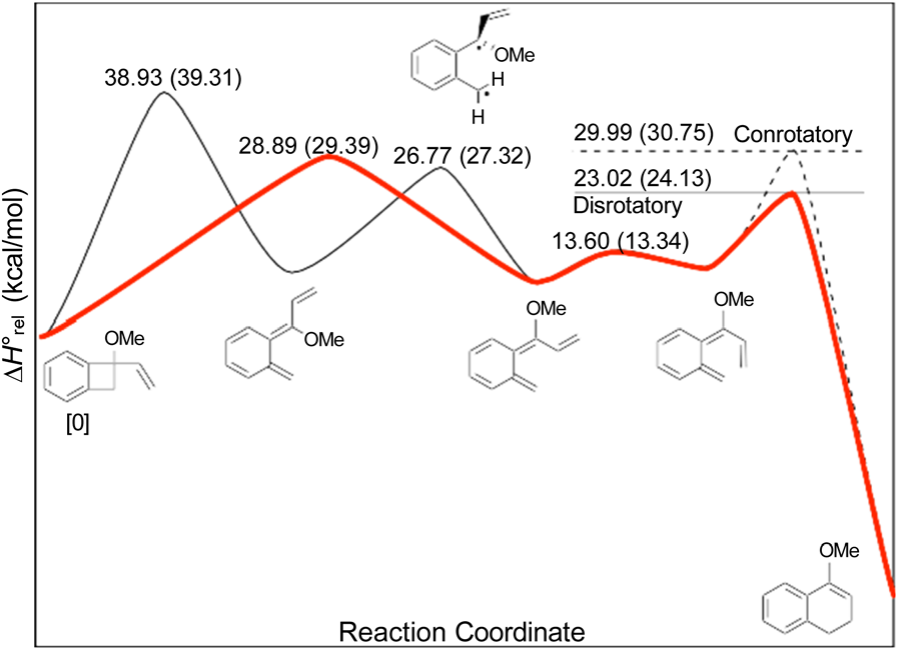
UM06-2X/cc-pVTZ enthalpy profile for the ring expansion of methoxy-vinyl-benzocyclobutene to 4-methoxy-1,2-dihydro-naphthalene. The minimum energy path is shown in red. Numbers in parentheses are relative Gibbs free energies at the reaction temperature of 110 °C.

In the Introduction, the work of Wallace's group^42^ was outlined, building on earlier research by Sammes. They created their alkenyl-benzocyclobutenes by adding Grignard reagents to benzocyclobutenone (Scheme 4). This meant that their ring expansions occurred in the presence of an alkoxy substituent. They recognised that it could have the benefit of controlling the sense of the initial conrotatory ring opening to drive the alkenyl substituent “inwards”, as required for the second electrocyclic reaction. As summarised in Fig. 8, the present calculations support that expectation.

**Scheme 9:**
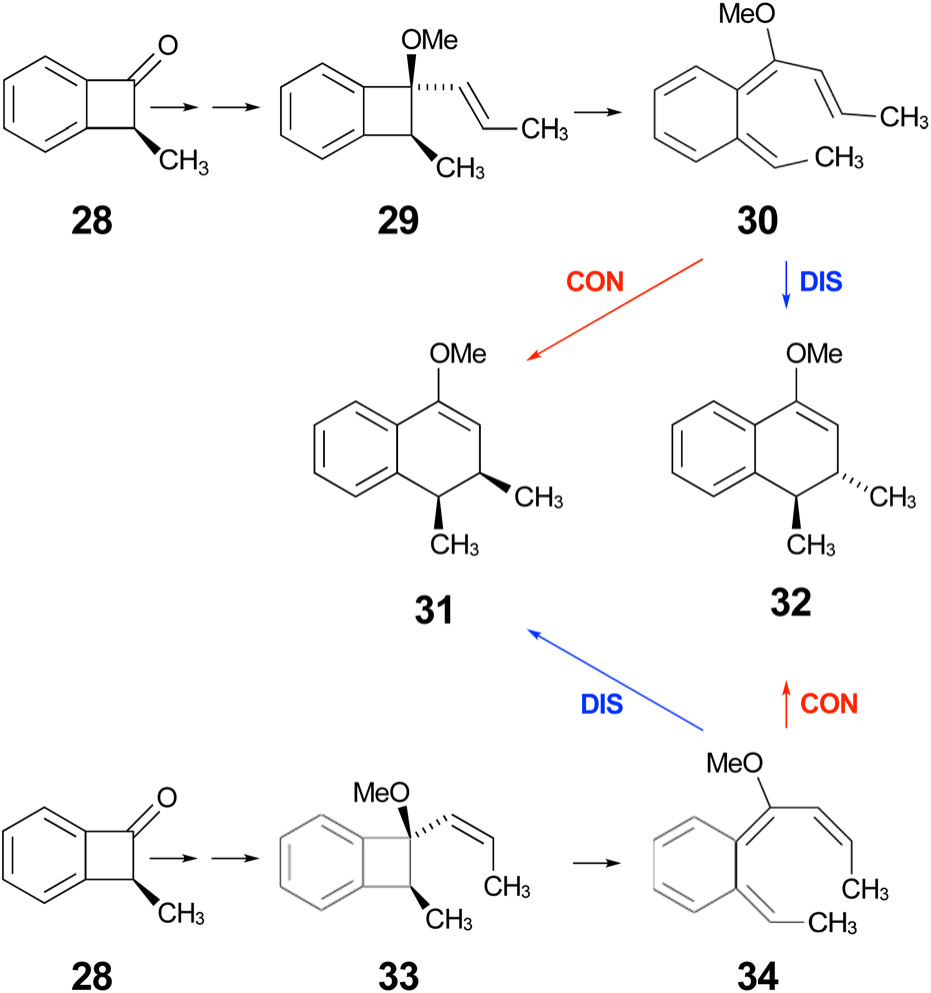
Expected stereochemical outcomes from the propenyl Grignard additions and subsequent ring expansions of ketone 28.

In the ring expansion of vinyl-benzocyclobutene, 4-*d*_2_, the calculations suggested that the overall stereochemistry was controlled by internal rotation of intermediate 5-*d*_2_, which had to take place before the final 6 π electrocyclic ring closure could occur (Fig. 7 and Scheme 8). However, in the case of the methoxy derivative (Fig. 8) the overall stereochemistry is controlled by the energy difference between conrotatory and disrotatory modes of the final step. This gap is found to be 7.0 kcal mol in enthalpy or 6.6 kcal mol^−1^ in free energy at the reaction temperature of 110 °C. Although significant, such an energy difference between “allowed” and “forbidden” mechanisms is not overwhelming, and one might wonder whether it could be overcome such mundane things as steric effects between substituents. That question was addressed by calculations on some methyl derivatives, as shown in Scheme 9.

Following the stereochemical arguments of Wallace and coworkers, the addition of propenyl Grignard reagents to ketone 28 should occur preferentially from the less hindered side to generate, after methylation, the compounds 29 and 33. The “torquoselectivity” of the methoxy substituent should influence the benzocyclobutene ring openings to give intermediates 30 and 34, and their final ring closures would give the dihydronaphthalene derivatives 31 and 32. One could anticipate that steric interactions between the methyl substituents would favour the *trans* over the *cis* isomer of the final products, and that this effect might be felt in the transition states for their formation.

**Fig. 9:**
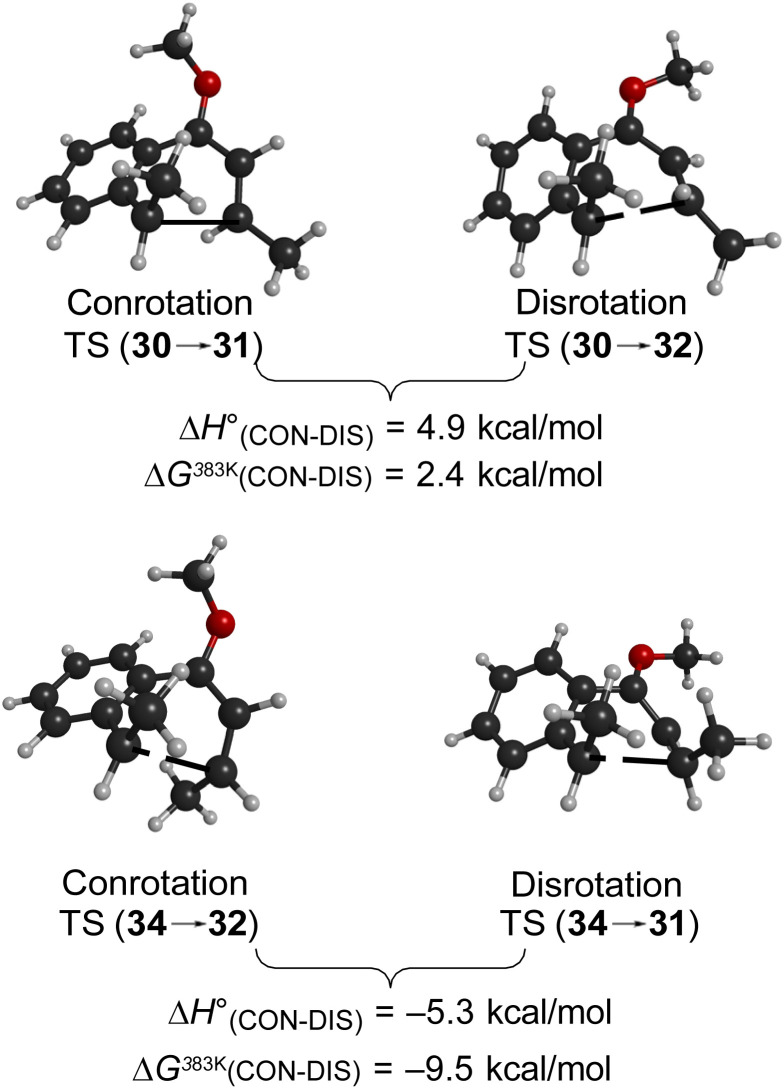
Relative UM06-2X/cc-pVTZ transition state energies.

As summarised in Fig. 9, UM06-2X/cc-pVTZ calculations supported those expectations. The lower half of Fig. 9 reveals that, indeed, the energy difference between “forbidden” and “allowed” transition states for the 6 π cyclisation could be overcome by a simple steric effect between methyl groups. However, there would not be much consequence for practical chemistry, because the calculations reveal that, for intermediate 34 (Scheme 9), the barrier to [1,7]-hydrogen migration is lower than either mode of electrocyclic ring closure, just as Maitland Jones^40^ had found for compound 10 (Scheme 2).

**Fig. 10:**
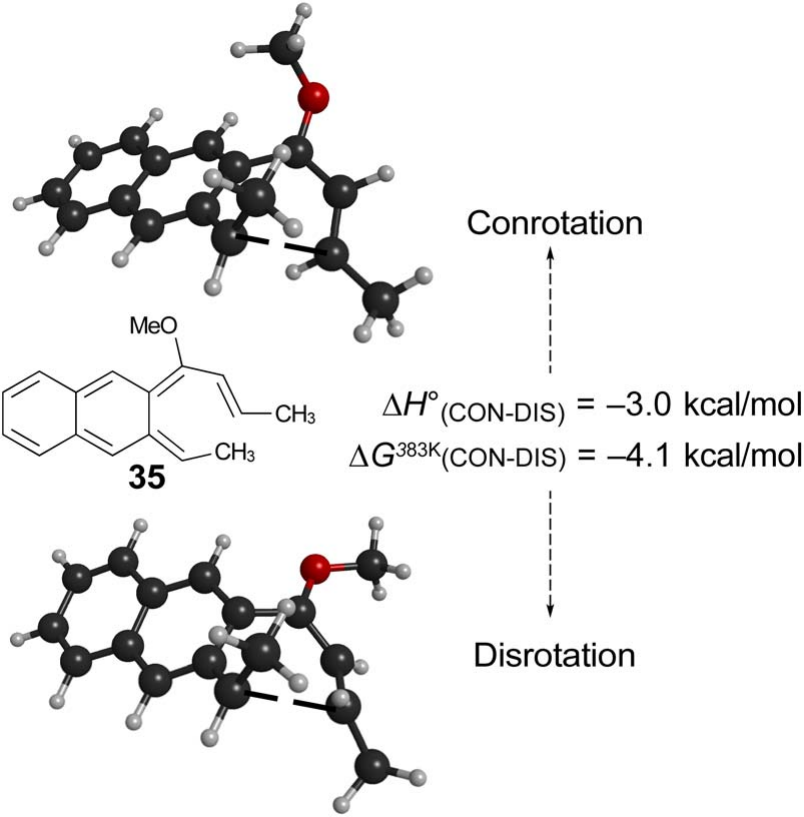
UM06-2X/cc-pVTZ results for the 6 π ring closure transition states of compound 35.

Of more interest is the small free energy difference between conrotation and disrotation for closure of intermediate 30 – only 2.4 kcal mol^−1^ according to the calculations. The calculations on reactions G and I (Scheme 6) had revealed that the potential energy gap between “forbidden” and “allowed” mechanisms was smaller for the naphthalene derivative than for its benzene analogue. It seemed possible, therefore, that the free energy difference for the methyl- and methoxy-substituted case (compound 35) might be smaller still, or possibly even inverted for the isomer that does complete the 6 π electrocyclization. As shown in Fig. 10, this was indeed how the calculations came out.

**Scheme 10:**
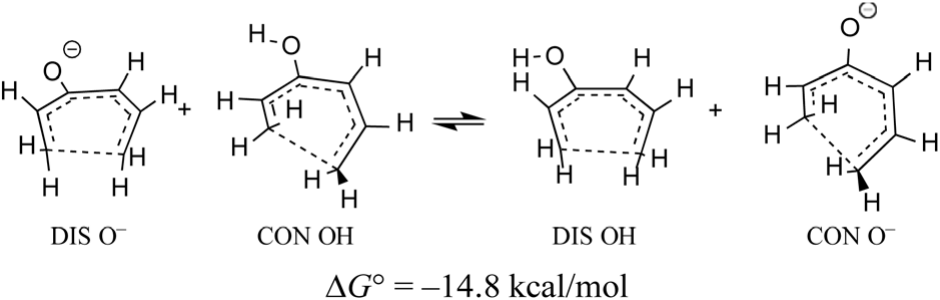
UM06-2X/aug-cc-pVTZ free energy change illustrating the relative gas-phase acidities of the disrotatory and conrotatory transition states for ring closure of (*E*)-1,3,5-hexatrien-3-ol. The free energy is calculated at 298.15 K and 1 atm.

When the energy gap between “forbidden” and “allowed” transition states is modest, one can anticipate that it might also be overcome by electronic substituent effects. In particular, it has previously been pointed out that π-type substituents (whether donors or acceptors) are likely to stabilise an antiaromatic transition state more than its aromatic counterpart.^78^ In the present context, that effect is exemplified by the calculated free energy change for a hypothetical homodesmotic proton transfer between conrotatory and disrotatory transition states for ring closure of (*E*)-1,3,5-hexatrien-3-ol and its conjugate base (Scheme 10).

**Scheme 11:**
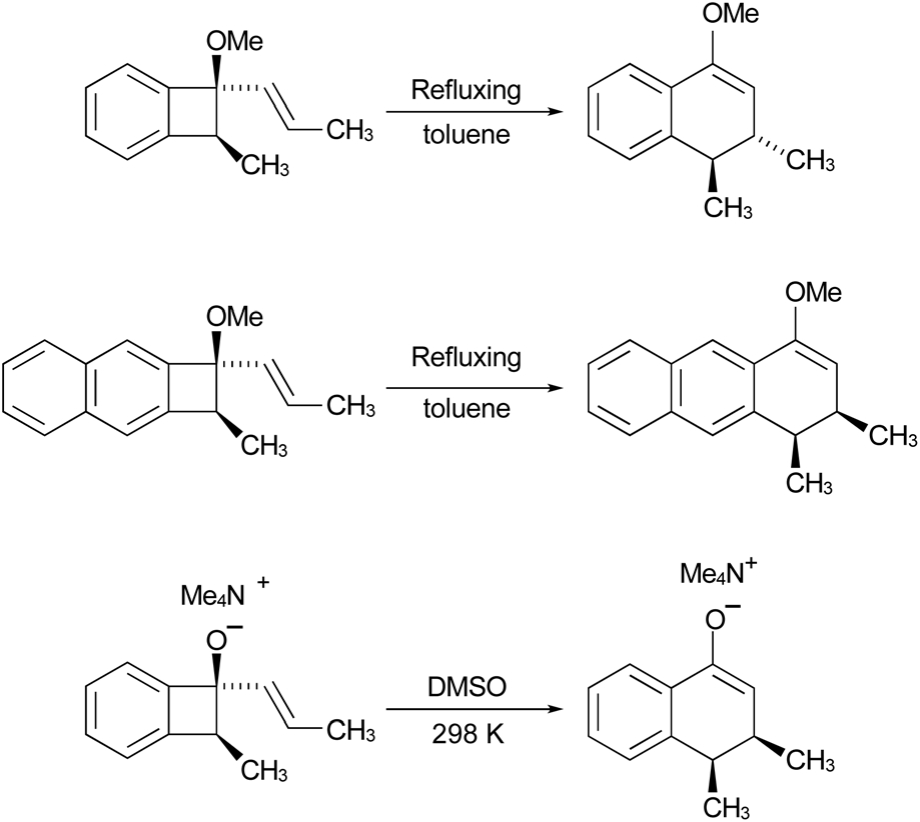
Predicted principal products from the reactions shown.

Rearranging the equilibrium in Scheme 10, so that the two anionic species are on one side and the two neutral species on the other, reveals that the free energy difference between conrotatory and disrotatory transition states is 14.8 kcal mol^−1^ smaller for the anions than for their conjugate acids. Hence, one can anticipate that, while the hydroxy analogue of compound 29 should give the same stereochemistry for the final product as 29 itself, the corresponding anion might give the opposite stereochemistry. UM06-2X/aug-cc-pVTZ calculations run using the SMD solvation model^79^ for DMSO, and employing a tetramethylammonium counterion, confirmed this expectation, with the nominally forbidden conrotatory closure being 3.1 kcal mol^−1^ lower in free energy than the disrotatory counterpart at 298 K. The lower temperature for this calculation is appropriate because the alkoxide substituent greatly accelerates the initial benzocyclobutene ring opening as well as changing the stereochemistry of the final ring closure (see the ESI† for details). The principal findings of this section are summarised in Scheme 11. The differences in stereochemistry arise directly from the phenomena outlined in Section 3.2 and serve to emphasise that an unthinking adherence to the binary selection rules for electrocyclic reactions can be unwise.

## 4. Conclusions

The principal conclusion from the present work is that modern electronic structure theory does not support a binary classification of electrocyclic reactions into “allowed” and “forbidden” sets. The electronic factors that formed the basis of the selection rules for such reactions are, of course, real but their energetic consequences are not always overwhelming. One sees that there is a continuous distribution of effects, ranging from strong aromatic stabilisation to strong antiaromatic destabilisation of the transition states, but with reactions in the middle of that spectrum for which the effects are small and comparable in magnitude to many other factors that affect reaction barriers, such as steric interactions. Although not as catchy as “allowed” and “forbidden,” electrocyclic reactions would more accurately be characterised as “Woodward–Hoffmann favoured” and “Woodward–Hoffmann disfavoured,” respectively.

In the process of carrying out the present calculations, it became necessary to address a historical disagreement on the nature of the transition states of “forbidden” electrocyclic reactions. It was found that the Berson–Salem “subjacent orbital” effect was real and did indeed favour the pericyclic conrotatory transition state for hexatriene ring closures over their stepwise diradical alternatives. This finding was in direct opposition to the claims of Dewar and Kirschner.^23^ However, for cyclobutene ring openings, the diradicals were found to be lower in energy than the disrotatory saddle points, which were of second order in most cases. The reason that Berson and Salem got it wrong in this case is probably that their model ignored σ-strain effects, which come into play for reactions involving four-membered rings. However, the fact that Berson and Salem were wrong does not mean that Dewar and Kirshner were correct, because the diradicals were not on the paths for the “forbidden” electrocyclic reactions, as they had assumed, but were instead transition states for internal rotation of the ring-opened dienes. It should be noted, however, that Dewar and Kirschner did recommend against the “allowed” and “forbidden” dichotomy many years ago,^80^ although their arguments were different from those presented here.

Prinzbach showed experimentally that vinylogous sesquifulvalene (compound 25) underwent 14 π electrocyclic ring closure by the “forbidden” conrotation. Although the transition state polarity, previously proposed as an explanation for this result, may play a role, the present calculations do not support its being the dominant effect because an isoelectronic nonpolar reaction is predicted to follow the same stereochemical course with an even stronger preference for conrotation. Instead, it is proposed that the principal effect in both cases comes from the weak antiaromaticity of the nominally forbidden transition state, which is then outweighed by a steric preference for conrotation over disrotation.

Calculations on some specific reactions that had been studied experimentally have led to predictions of stereochemical outcomes that would be different from those predicted by simple application of the W–H rules. The rearrangements of vinyl-benzocyclobutene (7-vinylbicyclo[4.2.0]octa-1,3,5-triene) and its derivatives was studied by Maitland Jones and coworkers. The W–H rules would predict a conrotatory ring opening of the four-membered ring, followed by a disrotatory ring closure to make a new six-membered ring. Had the authors been able to introduce deuterium labels to check this stereochemical outcome, the present calculations suggest that they would have been surprised. The predicted overall stereochemistry corresponds to two disrotatory steps. The reason, in this case, does not have to do with a failure of the W–H rules, but rather with the low barrier to internal rotation of the intermediate formed in the first step.

The situation is different for a similar series of reactions, proposed as a route to the synthesis of some anticancer compounds. In these reactions, a hydroxy or methoxy substituent controls the stereochemistry of the initial ring opening, directly producing the stereoisomer of the intermediate that is required for the final 6 π electrocyclisation. The overall stereochemistry of the reaction is then controlled by this final step. The calculations suggest that the benzocyclobutene derivatives should behave exactly as the authors had expected from application of the W–H rules to the two electrocyclic steps. However, for the analogous dihydrocyclo-buta[*b*]naphthalene derivatives, which would be the compounds needed for the synthesis of the anticancer compounds in which the authors were interested, the present calculations predict that the preferred stereochemistry of the final step would be the supposedly forbidden conrotation. Evidence is also presented that the stereochemistry of the ring expansion of compounds such as 17 (Scheme 4) would be reversed between the alcohol and its conjugate base. The failure of the W–H rule prediction in these cases can be traced to the weak antiaromaticity in the “forbidden” transition states.

## Data availability

The data supporting this article have been included as part of the ESI.†

## Author contributions

The sole author was responsible for all aspects of this work.

## Conflicts of interest

There are no conflicts to declare.

## Supplementary Material

SC-OLF-D4SC08748H-s001
