# Supplementary material for: An argument for abandoning the “allowed” and “forbidden” classification of electrocyclic reactions
Source: Chem Sci. 2025 Feb 19;16(10):4264–78. doi: 10.1039/d4sc08748h (PMC11837749; doi:10.1039/d4sc08748h)
Supplement: SC-016-D4SC08748H-s001 [file SC-016-D4SC08748H-s001.pdf]

Supplementary Information (SI) for Chemical Science.
This journal is © The Royal Society of Chemistry 2025

An Argument for Abandoning the "Allowed" and "Forbidden" Designation of Electrocyclic Reactions.

Electronic Supplementary Information

Calculations were carried out with Gaussian 09 Revision D.01[1], GAMESS-US version 30 SEP 2023 (R2)[2] and ORCA version 5.0.4.[3–5] All calculations used the cc-pVTZ basis set, except for those associated with Scheme 10 in the main text, which used aug-cc-pVTZ. All enthalpies and free energies listed here are at 1.0 atm, 298.15 K. The free energies listed as (HO) used the harmonic oscillator approximation, while those listed as (GR) used the Grimme formula for interpolating between harmonic oscillator and free rotor partition functions for low frequency vibrations. See the main text for citations of the basis set and the Grimme free energy interpolation. The descriptions of CASSCF active spaces and details of the NEVPT2 calculations are also provided in the main text.

The data here are organised to match their appearance in the main text. Consequently, references are given to Table, Figure, and Scheme numbers in the main text.

Data for main text Table 1.

Cyclobutene 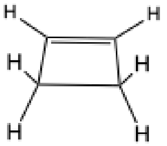

CASSCF(4,4) Optimized Geometry

C	-0.66900611	0.00000000	0.78022879
C	0.66900611	0.00000000	0.78022879
C	-0.79909372	0.00000000	-0.72723973
C	0.79909372	0.00000000	-0.72723973
H	-1.25028634	-0.88275439	-1.16333926
H	1.25028634	-0.88275439	-1.16333926
H	-1.25028634	0.88275439	-1.16333926
H	1.25028634	0.88275439	-1.16333926
H	-1.40451407	0.00000000	1.56126869
H	1.40451407	0.00000000	1.56126869

CASSCF Energy = -154.998588277
NEVPT2 Energy = -155.670028593
NIMAG = 0
CASSCF H = -154.903470644
CASSCF G (HO) = -154.933114675
CASSCF G (GR) = -154.933116698

M06-2X Optimized Geometry

C	-0.78074804	0.00000000	-0.72224702
C	-0.66533654	0.00000000	0.78544381
C	0.66533654	0.00000000	0.78544381
C	0.78074804	0.00000000	-0.72224702
H	1.23613725	0.88720784	-1.16170844
H	1.23613725	-0.88720784	-1.16170844
H	1.40944302	0.00000000	1.57038439
H	-1.40944302	0.00000000	1.57038439
H	-1.23613725	-0.88720784	-1.16170844

H	-1.23613725	0.88720784	-1.16170844

M06-2X Energy = -155.950732745
NIMAG = 0
M06-2X H = -155.858795234
M06-2X G (HO) = -155.888503779
M06-2X G (GR) = -155.888506018

ωB97X-D Optimized Geometry

C	0.78126024	0.00000000	-0.69691767
C	0.66546990	0.00000000	0.81043291
C	-0.66546990	0.00000000	0.81043291
C	-0.78126024	0.00000000	-0.69691767
H	-1.23779328	0.88687033	-1.13777697
H	-1.23779328	-0.88687033	-1.13777697
H	-1.41020061	0.00000000	1.59446246
H	1.41020061	0.00000000	1.59446246
H	1.23779328	-0.88687033	-1.13777697
H	1.23779328	0.88687033	-1.13777697

ωB97X-D Energy = -155.975191355
NIMAG = 0
ωB97X-D H = -155.882892506
ωB97X-D G (HO) = -155.912532628
ωB97X-D G (GR) = -155.912534644

PBE0-D3BJ Optimized Geometry

C	0.77939606	0.00000000	-0.69512644
C	0.66693178	0.00000000	0.80889875
C	-0.66693178	0.00000000	0.80889875
C	-0.77939606	0.00000000	-0.69512644
H	-1.23752506	0.88735980	-1.13857679
H	-1.23752506	-0.88735980	-1.13857679
H	-1.41305026	0.00000000	1.59451969
H	1.41305026	0.00000000	1.59451969
H	1.23752506	-0.88735980	-1.13857679
H	1.23752506	0.88735980	-1.13857679

PBE0-D3BJ Energy = -155.838136837
NIMAG = 0
PBE0-D3BJ H = -155.746677038
PBE0-D3BJ G (HO) = -155.776382404
PBE0-D3BJ G (GR) = -155.776384608

<u>s-Trans-1,3-butadiene</u>

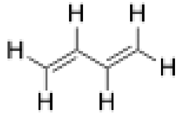

CASSCF(4,4) Optimized Geometry

C	-1.84643352	0.12347838	0.00000000
C	1.84643352	-0.12347838	0.00000000
C	-0.61323613	-0.39838782	0.00000000
C	0.61323613	0.39838782	0.00000000
H	-0.49325129	-1.46797657	0.00000000
H	0.49325129	1.46797657	0.00000000
H	-2.71859264	-0.50123978	0.00000000
H	2.71859264	0.50123978	0.00000000
H	-2.00759745	1.18599832	0.00000000
H	2.00759745	-1.18599832	0.00000000

CASSCF Energy = -155.031768944
NEVPT2 Energy = -155.694377011
NIMAG = 0
CASSCF H = -154.938106049
CASSCF G (HO) = -154.968843765
CASSCF G (GR) = -154.968837367

M06-2X Optimized Geometry

C	0.60571119	1.73377900	0.00000000
C	-0.60571119	-1.73377900	0.00000000
C	0.60572420	0.40499320	0.00000000
C	-0.60572420	-0.40499320	0.00000000
H	1.54696632	-0.13560431	0.00000000
H	-1.54696632	0.13560431	0.00000000
H	1.52485135	2.30213636	0.00000000
H	-1.52485135	-2.30213636	0.00000000
H	-0.32245185	2.29229582	0.00000000
H	0.32245185	-2.29229582	0.00000000

M06-2X Energy = -155.967859648
NIMAG = 0
M06-2X H = -155.876475052
M06-2X G (HO) = -155.907832185
M06-2X G (GR) = -155.907829644

ωB97X-D Optimized Geometry

C	0.60367545	1.73634390	0.00000000
C	-0.60367545	-1.73634390	0.00000000
C	0.60367545	0.40770630	0.00000000
C	-0.60367545	-0.40770630	0.00000000
H	1.54797931	-0.12784494	0.00000000
H	-1.54797931	0.12784494	0.00000000
H	1.52492821	2.30195563	0.00000000
H	-1.52492821	-2.30195563	0.00000000
H	-0.32252677	2.29844914	0.00000000
H	0.32252677	-2.29844914	0.00000000

ωB97X-D Energy = -155.989803645
NIMAG = 0
ωB97X-D H = -155.898461409
ωB97X-D G (HO) = -155.929226108
ωB97X-D G (GR) = -155.929219990

PBE0-D3BJ Optimized Geometry

C	0.60017652	1.73748979	0.00000000
C	-0.60017652	-1.73748979	0.00000000
C	0.60017652	0.40572623	0.00000000
C	-0.60017652	-0.40572623	0.00000000
H	1.54608730	-0.13046825	-0.00000000
H	-1.54608730	0.13046825	0.00000000
H	1.52133437	2.30579951	0.00000000
H	-1.52133437	-2.30579951	0.00000000
H	-0.32777250	2.29928150	-0.00000000
H	0.32777250	-2.29928150	0.00000000

PBE0-D3BJ Energy = -155.851366462
NIMAG = 0
PBE0-D3BJ H = -155.760522478
PBE0-D3BJ G (HO) = -155.791263538

PBE0-D3BJ G (GR) = -155.791262069

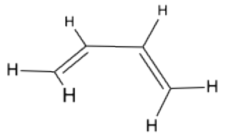

s-Cis-1,3-butadiene

CASSCF(4,4) Optimized Geometry

C	-0.72829452	0.11771020	0.53708780
C	0.72829452	-0.11771020	0.53708780
C	-1.54665875	-0.07382533	-0.50320619
C	1.54665875	0.07382533	-0.50320619
H	-1.19135314	-0.45304015	-1.44293427
H	1.19135314	0.45304015	-1.44293427
H	-2.59671688	0.13420536	-0.42705521
H	2.59671688	-0.13420536	-0.42705621
H	-1.14674896	0.46182266	1.46710372
H	1.14674896	-0.46182266	1.46710372

CASSCF Energy = -155.026959579
NEVPT2 Energy = -155.688672474
NIMAG = 0
CASSCF H = -154.933301986
CASSCF G (HO) = -154.965028492
CASSCF G (GR) = -154.965005019

M06-2X Optimized Geometry

C	0.10846941	-1.52008560	-0.50319507
C	-0.10943485	-0.72613433	0.53803809
C	0.10943485	0.72613433	0.53803809
C	-0.10846941	1.52008560	-0.50319507
H	-0.50564941	1.12983714	-1.43199182
H	0.08855203	2.58176126	-0.45318457
H	0.45547271	1.16060154	1.46999823
H	-0.45547271	-1.16060154	1.46999823
H	-0.08855203	-2.58176126	-0.45318457
H	0.50564941	-1.12983714	-1.43199182

M06-2X Energy = -155.963269792
NIMAG = 0
M06-2X H = -155.871935794
M06-2X G (HO) = -155.904062138
M06-2X G (GR) = -155.904063700

ωB97X-D Optimized Geometry

C	0.11009224	-1.52967145	-0.48622223
C	-0.11009224	-0.72535687	0.54662506
C	0.11009224	0.72535687	0.54662506
C	-0.11009224	1.52967145	-0.48622223
H	-0.51162356	1.15264323	-1.41868300
H	0.08787943	2.59079273	-0.42337526
H	0.46137480	1.15420582	1.47964128
H	-0.46137480	-1.15420582	1.47964128
H	-0.08787943	-2.59079273	-0.42337526
H	0.51162356	-1.15264323	-1.41868300

ωB97X-D Energy = -155.985346553
NIMAG = 0

ωB97X-D H = -155.893959695
ωB97X-D G (HO) = -155.925437037
ωB97X-D G (GR) = -155.925437783

PBE0-D3BJ Optimized Geometry

C	0.10318456	-1.52757225	-0.48911224
C	-0.10318456	-0.72324630	0.54974165
C	0.10318456	0.72324630	0.54974165
C	-0.10318456	1.52757225	-0.48911224
H	-0.48417204	1.14806644	-1.43061630
H	0.08804246	2.59121638	-0.42403955
H	0.43004908	1.15844176	1.49087936
H	-0.43004908	-1.15844176	1.49087936
H	-0.08804246	-2.59121638	-0.42403955
H	0.48417204	-1.14806644	-1.43061630

PBE0-D3BJ Energy = -155.846014201
NIMAG = 0
PBE0-D3BJ H = -155.755205180
PBE0-D3BJ G (HO) = -155.786809479
PBE0-D3BJ G (GR) = -155.786807599

First-Order Saddle Point for Conrotatory Ring Opening of Cyclobutene

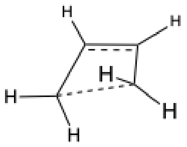

CASSCF(4,4) Optimized Geometry

C	-1.10956824	-0.10397121	0.66741037
C	1.10956824	0.10397121	0.66741037
C	-0.67501724	0.07505514	-0.69446248
C	0.67501724	-0.07505514	-0.69446248
H	-1.31667936	0.31506959	-1.52266693
H	1.31667936	-0.31506959	-1.52266693
H	-0.96844411	-1.06569004	1.11482871
H	0.96844411	1.06569004	1.11482871
H	-1.91464007	0.47512308	1.09410882
H	1.91464007	-0.47512308	1.09410882

CASSCF Energy = -154.942407103
NEVPT2 Energy = -155.617808792
NIMAG = 1
CASSCF H = -154.851067409
CASSCF G (HO) = -154.880968195
CASSCF G (GR) = -154.880969789

M06-2X Optimized Geometry

C	-0.12570777	-1.05178268	-0.64409340
C	0.08727184	-0.68119160	0.70460266
C	-0.08727184	0.68119160	0.70460266
C	0.12570777	1.05178268	-0.64409340
H	0.41146249	-1.86764547	-1.12598242
H	-1.07570132	-0.81895140	-1.09877541
H	0.41146277	-1.33562247	1.50374926
H	-0.41146277	1.33562247	1.50374926
H	-0.41146249	1.86764547	-1.12598242
H	1.07570132	0.81895140	-1.09877541

M06-2X Energy = -155.892513287
NIMAG = 1

M06-2X H = -155.803165393
M06-2X G (HO) = -155.833446412
M06-2X G (GR) = -155.833447455

ωB97X-D Optimized Geometry

C	-1.05592278	-0.61987155	-0.12331702
C	-0.68049484	0.72718514	0.08620619
C	0.68049483	0.72718515	-0.08620619
C	1.05592278	-0.61987156	0.12331702
H	-1.87649765	-1.09376988	0.41489627
H	-0.83181123	-1.07842899	-1.07380087
H	-1.33416334	1.52831734	0.40770874
H	1.33416336	1.52831735	-0.40770872
H	1.87649767	-1.09376988	-0.41489627
H	0.83181124	-1.07842901	1.07380087

ωB97X-D Energy = -155.914284298
NIMAG = 1
ωB97X-D H = -155.825002247
ωB97X-D G (HO) = -155.854633028
ωB97X-D G (GR) = -155.854634074

PBE0-D3BJ Optimized Geometry

C	-1.05674240	-0.62071390	-0.12237017
C	-0.68044483	0.72838636	0.08465386
C	0.68044486	0.72838631	-0.08465385
C	1.05674243	-0.62071386	0.12237019
H	-1.87396432	-1.10020538	0.41951572
H	-0.82930201	-1.07909537	-1.07429899
H	-1.33577419	1.53326603	0.40053896
H	1.33577410	1.53326597	-0.40053907
H	1.87396420	-1.10020541	-0.41951571
H	0.82930191	-1.07909530	1.07429895

PBE0-D3BJ Energy = -155.779607386
NIMAG = 1
PBE0-D3BJ H = -155.690887526
PBE0-D3BJ G (HO) = -155.720559040
PBE0-D3BJ G (GR) = -155.720560136

Second-Order Saddle Point for Disrotatory Ring Opening of Cyclobutene

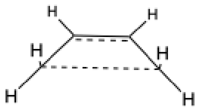

CASSCF(4,4) Optimized Geometry

C	0.68506002	-0.03945762	-1.43060720
C	0.68506002	-0.03945762	1.43060720
C	-0.58239275	-0.03713223	-0.66792512
C	-0.58239275	-0.03713223	0.66792512
H	-1.51249266	-0.03461505	-1.21239758
H	-1.51249266	-0.03461505	1.21239758
H	0.88218814	0.73685557	-2.14519739
H	0.88218814	0.73685557	2.14519739
H	1.25986648	-0.94124514	-1.52146375
H	1.25986648	-0.94124514	1.52146375

CASSCF Energy = -154.920735969
NEVPT2 Energy = -155.571313041
NIMAG = 2

CASSCF H = -154.833966027
CASSCF G (HO) = -154.866069357
CASSCF G (GR) = -154.866072169

First-Order Saddle Point for Internal Rotation of Terminal Methylene in s-Cis-1,3-butadiene

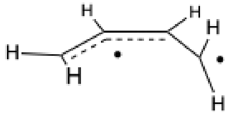

CASSCF(4,4) Optimized Geometry

C	-0.00126686	0.00399511	0.00000000
C	0.86888671	-1.18529117	0.00000000
C	2.25872660	-1.15504634	0.00000000
C	3.05062056	-0.01699630	0.00000000
H	2.62430048	0.96639556	0.00000000
H	4.11943674	-0.09431411	0.00000000
H	2.76071572	-2.10675812	0.00000000
H	0.39070296	-2.14984035	0.00000000
H	-0.41018537	0.38268486	-0.91736770
H	-0.41018537	0.38268486	0.91736770

CASSCF Energy = -154.939172612
NEVPT2 Energy = -155.597173247
NIMAG = 1
CASSCF H = -154.851021822
CASSCF G (HO) = -154.883431481
CASSCF G (GR) = -154.883438068

Bicyclo[4.2.0]octa-1,3,5-triene (Benzocyclobutene)

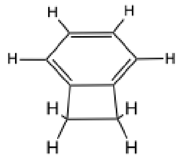

CASSCF(8,8) Optimized Geometry

C	-0.80508596	0.00000000	-1.98805881
C	0.80508596	0.00000000	-1.98805881
C	-0.69200629	0.00000000	-0.47625870
C	0.69200629	0.00000000	-0.47625870
C	-1.43341994	0.00000000	0.69244635
C	1.43341994	0.00000000	0.69244635
C	-0.69923484	0.00000000	1.88377833
C	0.69923484	0.00000000	1.88377833
H	-1.21715415	0.00000000	2.82434940
H	1.21715415	0.00000000	2.82434940
H	-2.50686049	0.00000000	0.70774430
H	2.50686049	0.00000000	0.70774430
H	-1.25160301	-0.88253999	-2.42714977
H	1.25160301	-0.88253999	-2.42714977
H	-1.25160301	0.88253999	-2.42714977
H	1.25160301	0.88253999	-2.42714977

CASSCF Energy = -307.752734636
NEVPT2 Energy = -309.056601280
NIMAG = 0
CASSCF H = -307.605622235
CASSCF G (HO) = -307.642050810
CASSCF G (GR) = -307.642057269

M06-2X Optimized Geometry

C	-0.07092536	1.42959176	-0.69262309
C	-0.03454756	0.69622309	-1.87952257
C	0.03454756	-0.69622309	-1.87952257

C	0.07092536	-1.42959176	-0.69262309
C	0.03416661	-0.69234826	0.47399653
C	-0.03416661	0.69234826	0.47399653
C	-0.03833956	0.78452144	1.98733679
C	0.03833956	-0.78452144	1.98733679
H	-0.12453034	2.50998684	-0.70870964
H	-0.06079478	1.21732940	-2.82725454
H	0.06079478	-1.21732940	-2.82725454
H	0.12453034	-2.50998684	-0.70870964
H	-0.94601165	1.19336421	2.42849981
H	0.82573919	1.27942416	2.42786810
H	0.94601165	-1.19336421	2.42849981
H	-0.82573919	-1.27942416	2.42786810

M06-2X Energy = -309.596466113
NIMAG = 0
M06-2X H = -309.454007017
M06-2X G (HO) = -309.490048758
M06-2X G (GR) = -309.490053705

ωB97X-D Optimized Geometry

C	0.00000000	1.43011564	-0.69166902
C	0.00000000	0.69649340	-1.87751054
C	0.00000000	-0.69649340	-1.87751054
C	0.00000000	-1.43011564	-0.69166902
C	0.00000000	-0.69250408	0.47282276
C	0.00000000	0.69250408	0.47282276
C	0.00000000	0.78612433	1.98535571
C	0.00000000	-0.78612433	1.98535571
H	0.00000000	2.51200266	-0.70733973
H	0.00000000	1.21785901	-2.82566669
H	0.00000000	-1.21785901	-2.82566669
H	0.00000000	-2.51200266	-0.70733973
H	-0.88657258	1.23948311	2.42733869
H	0.88657258	1.23948311	2.42733869
H	0.88657258	-1.23948311	2.42733869
H	-0.88657258	-1.23948311	2.42733869

ωB97X-D Energy = -309.624873661
NIMAG = 0
ωB97X-D H = -309.482221214
ωB97X-D G (HO) = -309.518181889
ωB97X-D G (GR) = -309.518188347

PBE0-D3BJ Optimized Geometry

C	0.00000000	-1.43165222	-0.69141434
C	0.00000000	-0.69666504	-1.87709486
C	0.00000000	0.69666504	-1.87709486
C	0.00000000	1.43165222	-0.69141434
C	0.00000000	0.69395993	0.47397339
C	0.00000000	-0.69395993	0.47397339
C	0.00000000	-0.78440670	1.98354299
C	0.00000000	0.78440670	1.98354299
H	0.00000000	-2.51526971	-0.70802922
H	0.00000000	-1.21871203	-2.82682501
H	0.00000000	1.21871203	-2.82682501
H	0.00000000	2.51526971	-0.70802922
H	0.88703137	-1.23940039	2.42821337
H	-0.88703137	-1.23940039	2.42821337
H	-0.88703137	1.23940039	2.42821337

H 0.88703137 1.23940039 2.42821337

PBE0-D3BJ Energy = -309.371054882
NIMAG = 0
PBE0-D3BJ H = -309.229210234
PBE0-D3BJ G (HO) = -309.265296383
PBE0-D3BJ G (GR) = -309.265300911

5,6-Dimethylenecyclohexa-1,3-diene (Orthoquinodimethane, Orthoxylylene)

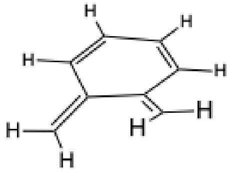

CASSCF(8,8) Optimized Geometry

C	1.41101837	0.00881500	0.66438454
C	0.72871190	0.00576092	1.82344151
C	-0.72871190	-0.00576092	1.82344151
C	-1.41101837	-0.00881500	0.66438454
C	-0.74464327	0.00024540	-0.64101148
C	0.74464327	-0.00024540	-0.64101148
C	1.49850297	-0.01116256	-1.76161242
C	-1.49850297	0.01116256	-1.76161242
H	2.48447680	0.01551438	0.66800553
H	1.25043094	0.01092274	2.76104045
H	-1.25043094	-0.01092274	2.76104045
H	-2.48447680	-0.01551438	0.66800553
H	1.08036745	-0.02218246	-2.74723339
H	2.56932783	-0.00972383	-1.69495046
H	-2.56932783	0.00972383	-1.69495046
H	-1.08036745	0.02218246	-2.74723339

CASSCF Energy = -307.752608966
NEVPT2 Energy = -309.037818184
NIMAG = 0
CASSCF H = -307.608059156
CASSCF G (HO) = -307.647851272
CASSCF G (GR) = -307.646380198

M06-2X Optimized Geometry

C	1.40134395	-0.17889956	-0.65457021
C	0.71795505	-0.11485707	-1.80363201
C	-0.71795505	0.11485707	-1.80363201
C	-1.40134395	0.17889956	-0.65457021
C	-0.74368167	-0.00277277	0.63727847
C	0.74368167	0.00277277	0.63727847
C	1.47190152	0.22483929	1.73835762
C	-1.47190152	-0.22483929	1.73835762
H	2.47540964	-0.31414786	-0.65293827
H	1.23110713	-0.21346469	-2.75043587
H	-1.23110713	0.21346469	-2.75043587
H	-2.47540964	0.31414786	-0.65293827
H	1.01727433	0.46368709	2.68929511
H	2.55245519	0.19347427	1.69791016
H	-2.55245519	-0.19347427	1.69791016
H	-1.01727433	-0.46368709	2.68929511

M06-2X Energy = -309.571264875
NIMAG = 0
M06-2X H = -309.430218317
M06-2X G (HO) = -309.468533752
M06-2X G (GR) = -309.468323496

ωB97X-D Optimized Geometry

C	-0.71635038	-0.11617912	1.80302250
C	0.71635038	0.11617912	1.80302250
C	-1.39907205	-0.18029378	0.65404046
C	1.39907205	0.18029378	0.65404046
C	-0.74359840	0.00413116	-0.63747132
C	0.74359840	-0.00413116	-0.63747132
C	-1.47298110	0.22982639	-1.73709118
C	1.47298110	-0.22982639	-1.73709118
H	-1.22848403	-0.21921068	2.75001049
H	1.22848403	0.21921068	2.75001049
H	-2.47256565	-0.31969902	0.65518832
H	2.47256565	0.31969902	0.65518832
H	-2.55366874	0.19983539	-1.69647777
H	2.55366874	-0.19983539	-1.69647777
H	-1.02093625	0.46676171	-2.68973398
H	1.02093625	-0.46676171	-2.68973398

ωB97X-D Energy = -309.595930841
NIMAG = 0
ωB97X-D H = -309.454818589
ωB97X-D G (HO) = -309.492475700
ωB97X-D G (GR) = -309.492260255

PBE0-D3BJ Optimized Geometry

C	0.71381563	0.10921979	1.80418587
C	-0.71381563	-0.10921979	1.80418587
C	1.39791882	0.17166351	0.65051645
C	-1.39791882	-0.17166351	0.65051645
C	0.74141848	-0.00146877	-0.63305074
C	-0.74141848	0.00146877	-0.63305074
C	1.47303581	-0.21808818	-1.73853815
C	-1.47303581	0.21808818	-1.73853815
H	1.23131633	0.20104086	2.75143147
H	-1.23131633	-0.20104086	2.75143147
H	2.47462487	0.29694176	0.65003902
H	-2.47462487	-0.29694176	0.65003902
H	2.55465221	-0.18206777	-1.69802070
H	-2.55465221	0.18206777	-1.69802070
H	1.01968551	-0.45587587	-2.69175053
H	-1.01968551	0.45587587	-2.69175053

PBE0-D3BJ Energy = -309.341108636
NIMAG = 0
PBE0-D3BJ H = -309.200836066
PBE0-D3BJ G (HO) = -309.238626671
PBE0-D3BJ G (GR) = -309.238377490

First-Order Saddle Point for Conrotatory Ring Opening of Bicyclo[4.2.0]hexa-1,3,5-triene (Benzoyclobutene)

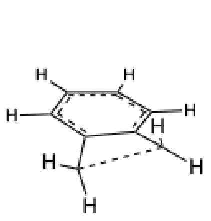

CASSCF(8,8) Optimized Geometry

C	-0.69533193	0.00405641	0.53526127
C	0.69533193	-0.00405641	0.53526127
C	-1.17517841	-0.20772499	1.88936555
C	1.17517841	0.20772499	1.88936555

C -1.41031313 0.12254327 -0.66871893
C 1.41031313 -0.12254327 -0.66871893
C -0.70086849 0.07690535 -1.85397148
C 0.70086849 -0.07690535 -1.85397148
H -0.93934876 -1.13999677 2.35868144
H 0.93934876 1.13999677 2.35868144
H -2.04632878 0.29810685 2.27095270
H 2.04632878 -0.29810685 2.27095270
H -1.22150779 0.12977061 -2.79118967
H 1.22150779 -0.12977061 -2.79118967
H -2.48191571 0.19160488 -0.67237234
H 2.48191571 -0.19160488 -0.67237234

CASSCF Energy = -307.692846788
NEVPT2 Energy = -308.992775250
NIMAG = 1
CASSCF H = -307.550629239
CASSCF G (HO) = -307.586892626
CASSCF G (GR) = -307.586898970

M06-2X Optimized Geometry

C 0.21032518 1.41450959 -0.66439241
C 0.12896261 0.69743157 -1.83126920
C -0.12896261 -0.69743157 -1.83126920
C -0.21032518 -1.41450959 -0.66439241
C -0.00014769 -0.70533103 0.53529810
C 0.00014769 0.70533103 0.53529810
C -0.29146045 1.09651237 1.86162922
C 0.29146045 -1.09651237 1.86162922
H 0.34285622 2.48853108 -0.67245100
H 0.22387758 1.20693924 -2.78064675
H -0.22387758 -1.20693924 -2.78064675
H -0.34285622 -2.48853108 -0.67245100
H -1.17183125 0.68408924 2.32570749
H 0.08342550 2.01620048 2.30387581
H -0.08342550 -2.01620048 2.30387581
H 1.17183125 -0.68408924 2.32570749

M06-2X Energy = -309.523779488
NIMAG = 1
M06-2X H = -309.384570281
M06-2X G (HO) = -309.421232216
M06-2X G (GR) = -309.421240455

ωB97X-D Optimized Geometry

C 0.20754791 1.41246794 -0.66332867
C 0.12739525 0.69660147 -1.83000392
C -0.12739525 -0.69660147 -1.83000392
C -0.20754791 -1.41246794 -0.66332867
C 0.00000000 -0.70426482 0.53488443
C 0.00000000 0.70426482 0.53488443
C -0.28885353 1.09957291 1.85991654
C 0.28885353 -1.09957291 1.85991654
H 0.33991774 2.48654637 -0.67203344
H 0.22253082 1.20597777 -2.77960866
H -0.22253082 -1.20597777 -2.77960866
H -0.33991774 -2.48654637 -0.67203344
H -1.17138661 0.69460252 2.32628684
H 0.08983034 2.01950916 2.29855430
H -0.08983034 -2.01950916 2.29855430

H	1.17138661	-0.69460252	2.32628684

ωB97X-D Energy = -309.549316432
NIMAG = 1
ωB97X-D H = -309.409962709
ωB97X-D G (HO) = -309.445912653
ωB97X-D G (GR) = -309.445920863

PBE0-D3BJ Optimized Geometry

C	0.20504777	1.41348609	-0.66129875
C	0.12558475	0.69485018	-1.83122093
C	-0.12558475	-0.69485018	-1.83122093
C	-0.20504777	-1.41348609	-0.66129875
C	0.00000000	-0.70587141	0.53316309
C	0.00000000	0.70587141	0.53316309
C	-0.28913152	1.09980717	1.86038940
C	0.28913152	-1.09980717	1.86038940
H	0.32993416	2.49010132	-0.67115331
H	0.21604297	1.20712999	-2.78170317
H	-0.21604297	-1.20712999	-2.78170317
H	-0.32993416	-2.49010132	-0.67115331
H	-1.17342224	0.69052661	2.32440342
H	0.09146184	2.01712906	2.30683839
H	-0.09146184	-2.01712906	2.30683839
H	1.17342224	-0.69052661	2.32440342

PBE0-D3BJ Energy = -309.299881829
NIMAG = 1
PBE0-D3BJ H = -309.161446500
PBE0-D3BJ G (HO) = -309.197533456
PBE0-D3BJ G (GR) = -309.197541659

Second-Order Saddle Point for Disrotatory Ring Opening of Bicyclo[4.2.0]hexa-1,3,5-triene (Benzoycyclobutene)

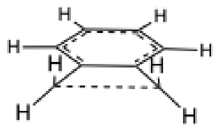

CASSCF(8,8) Optimized Geometry

C	-1.86526775	0.04559825	-1.34946764
C	-1.86526775	0.04559825	1.34946764
C	-0.53482860	-0.00871353	-0.70003396
C	-0.53482860	-0.00871353	0.70003396
C	1.87841296	-0.03391083	-0.69407254
C	1.87841296	-0.03391083	0.69407254
C	0.66935503	-0.01586942	-1.39362609
C	0.66935503	-0.01586942	1.39362609
H	0.67086536	0.00718431	-2.46747422
H	0.67086536	0.00718431	2.46747422
H	2.80708528	-0.03544297	-1.23204482
H	2.80708528	-0.03544297	1.23204482
H	-2.05022502	0.75104165	-2.13706636
H	-2.05022502	0.75104165	2.13706636
H	-2.51571584	-0.80610675	-1.30216420
H	-2.51571584	-0.80610675	1.30216420

CASSCF Energy = -307.680606452
NEVPT2 Energy = -308.966124980
NIMAG = 2
CASSCF H = -307.541558757
CASSCF G (HO) = -307.578881844

CASSCF G (GR) = -307.578892252

First-Order Saddle Point for Internal Rotation of Methylene in 5,6-Dimethylenecyclohexa-1,3-diene (Orthoquinodimethane, Orthoxylylene)

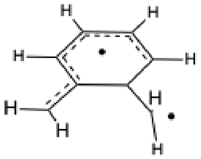

CASSCF(8,8) Optimized Geometry

C	-0.55499148	0.73802633	0.00000000
C	-0.61222583	-0.69414471	0.00000000
C	0.57160255	-1.41885599	0.00000000
C	1.82004722	-0.78930988	0.00000000
C	1.88904922	0.60609668	0.00000000
C	0.72823514	1.35269075	0.00000000
C	-1.90939345	-1.41190935	0.00000000
C	-1.71059695	1.53607078	0.00000000
H	0.78191318	2.42500846	0.00000000
H	2.84279353	1.09833355	0.00000000
H	2.71687857	-1.37767980	0.00000000
H	0.52293700	-2.49134327	0.00000000
H	-2.44527087	-1.56374642	-0.91720751
H	-2.44527087	-1.56374642	0.91720751
H	-2.69324963	1.11307930	0.00000000
H	-1.63081310	2.60481311	0.00000000

CASSCF Energy = -307.692673020
NEVPT2 Energy = -308.980583413
NIMAG = 1
CASSCF H = -307.552745091
CASSCF G (HO) = -307.590745619
CASSCF G (GR) = -307.590743877

Data for main text Scheme 5.

Data for reactions **A** and **C** are provided above.

Reaction B

1,2-Divinylcyclobutene

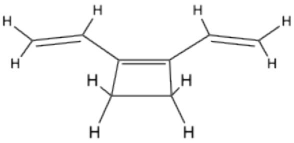

CASSCF(8,8) Optimized Geometry

C	-3.00458503	0.00000000	-0.74461900
C	3.00458503	0.00000000	-0.74461900
C	-1.68365812	0.00000000	-0.97338788
C	1.68365812	0.00000000	-0.97338788
C	-0.79678398	0.00000000	1.58191295
C	0.79678398	0.00000000	1.58191295
C	-0.67634839	0.00000000	0.07336356
C	0.67634839	0.00000000	0.07336356
H	-3.71056485	0.00000000	-1.55239551
H	3.71056485	-0.00000000	-1.55239551
H	-3.40418196	0.00000000	0.25231807
H	3.40418196	-0.00000000	0.25231807
H	-1.32893872	0.00000000	-1.98907821
H	1.32893872	0.00000000	-1.98907821
H	-1.24795234	-0.88299727	2.01803763
H	1.24795234	-0.88299727	2.01803763

H	-1.24795234	0.88299727	2.01803763
H	1.24795234	0.88299727	2.01803763

CASSCF Energy = -308.884323491
NEVPT2 Energy = -310.201584710
NIMAG = 0
CASSCF H = -308.715703482
CASSCF G (HO) = -308.756695860
CASSCF G (GR) = -308.756491579

First-Order Saddle Point for Conrotatory Ring Opening of 1,2-Divinylcyclobutene

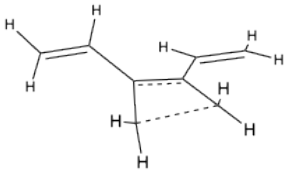

CASSCF(8,8) Optimized Geometry

C	-1.59848607	0.19483744	-0.89014441
C	1.59848607	-0.19483744	-0.89014441
C	-2.91801953	-0.04082541	-0.86940992
C	2.91801953	0.04082541	-0.86940992
C	-0.68851745	-0.03600493	0.23147944
C	0.68851745	0.03600493	0.23147944
C	-1.07662761	-0.29719692	1.59152460
C	1.07662761	0.29719692	1.59152460
H	-1.16132188	0.60308456	-1.78391194
H	1.16132188	-0.60308456	-1.78391194
H	-3.53546715	0.19029383	-1.71587026
H	3.53546715	-0.19029383	-1.71587026
H	-3.40586209	-0.46712855	-0.01322674
H	3.40586209	0.46712855	-0.01322674
H	-0.75629306	-1.21036792	2.04739332
H	0.75629306	1.21036792	2.04739332
H	-1.96476150	0.14004421	2.02210450
H	1.96476150	-0.14004421	2.02210450

CASSCF Energy = -308.823159574
NEVPT2 Energy = -310.146138567
NIMAG = 1
CASSCF H = -308.658440262
CASSCF G (HO) = -308.699407403
CASSCF G (GR) = -308.699166086

Second-Order Saddle Point for Disrotatory Ring Opening of 1,2-Divinylcyclobutene

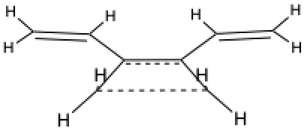

CASSCF(8,8) Optimized Geometry

C	0.25007772	-0.00841715	-0.68239874
C	0.25007772	-0.00841715	0.68239874
C	1.58376539	-0.03509228	-1.32241488
C	1.58376539	-0.03509228	1.32241488
C	-0.95167154	-0.02410028	-1.52128649
C	-0.95167154	-0.02410028	1.52128649
C	-0.96106052	0.04710506	-2.86145234
C	-0.96106052	0.04710506	2.86145234
H	-1.89831674	-0.10073759	-1.02358639
H	-1.89831674	-0.10073759	1.02358639
H	2.25695872	0.79180956	-1.21698070
H	2.25695872	0.79180956	1.21698070
H	1.78597081	-0.72893047	-2.11496305

H	1.78597081	-0.72893047	2.11496305
H	-0.06336749	0.13611864	-3.44167018
H	-0.06336749	0.13611864	3.44167018
H	-1.88678753	0.02282986	-3.40326786
H	-1.88678753	0.02282986	3.40326786

CASSCF Energy = -308.795513076
NEVPT2 Energy = -310.099308563
NIMAG = 2
CASSCF H = -308.635113261
CASSCF G (HO) = -308.677332352
CASSCF G (GR) = -308.677118876

First-Order Saddle Point for Internal Rotation of Methylene in 3,4-Dimethylenehexa-1,5-diene

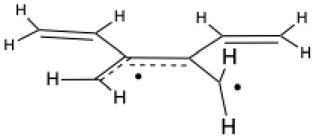

CASSCF(8,8) Optimized Geometry

C	-1.46258795	-0.90903246	0.00000000
C	-2.82284951	-0.99478179	0.00000000
C	-0.71057564	0.30695269	0.00000000
C	-1.46865499	1.58074129	0.00000000
C	0.73118645	0.35738510	0.00000000
C	1.37461686	1.56876242	0.00000000
C	1.52452183	-0.90076905	0.00000000
C	2.85875964	-1.01191235	0.00000000
H	-3.45553994	-0.12987463	0.00000000
H	-3.30423522	-1.95305407	0.00000000
H	-1.71453428	2.07508349	-0.91950971
H	-1.71453428	2.07508349	0.91950971
H	2.44181299	1.64034462	0.00000000
H	0.83455420	2.49183321	0.00000000
H	3.52604795	-0.17292345	0.00000000
H	3.31598306	-1.98266006	0.00000000
H	0.97595668	-1.81890368	0.00000000
H	-0.93086571	-1.83818400	0.00000000

CASSCF Energy = -308.809678981
NEVPT2 Energy = -310.119214601
NIMAG = 1
CASSCF H = -308.649125080
CASSCF G (HO) = -308.691384663
CASSCF G (GR) = -308.690923099

Reaction D

1,2-Dihydrocyclobuta[b]naphthalene

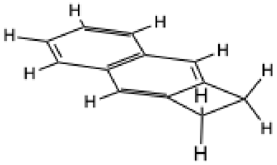

CASSCF(12,12) Optimized Geometry

C	-0.80489999	0.00000000	-3.22754326
C	0.80489999	-0.00000000	-3.22754326
C	-0.70449769	0.00000000	-1.71394220
C	0.70449769	0.00000000	-1.71394220
C	-1.43801999	0.00000000	-0.56934842
C	1.43801999	0.00000000	-0.56934842
C	-0.71053272	0.00000000	0.66426036
C	0.71053272	0.00000000	0.66426036
C	-1.39221334	0.00000000	1.91238687
C	1.39221334	-0.00000000	1.91238687

C	-0.70759457	0.00000000	3.09764394
C	0.70759457	0.00000000	3.09764394
H	-1.25114882	-0.88231409	-3.66658893
H	1.25114882	-0.88231409	-3.66658893
H	-1.25114882	0.88231409	-3.66658893
H	1.25114882	0.88231409	-3.66658893
H	-2.51195049	0.00000000	-0.55362281
H	2.51195049	0.00000000	-0.55362281
H	-2.46628141	0.00000000	1.91160426
H	2.46628141	-0.00000000	1.91160426
H	-1.24093640	0.00000000	4.02893815
H	1.24093640	-0.00000000	4.02893815

CASSCF Energy = -460.499167155
NEVPT2 Energy = -462.432357274
NIMAG = 0
CASSCF H = -460.300845020
CASSCF G (HO) = -460.342606345
CASSCF G (GR) = -460.342529526

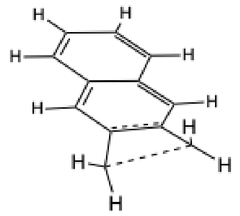

First-Order Saddle Point for Conrotatory Ring Opening of Cyclobuta[b]naphthalene

CASSCF(12,12) Optimized Geometry

C	-0.70996088	0.05425118	-3.07458425
C	0.70996088	-0.05425118	-3.07458425
C	-1.39362049	0.10646672	-1.89574897
C	1.39362049	-0.10646672	-1.89574897
C	-0.70903796	0.05586032	-0.64189601
C	0.70903796	-0.05586032	-0.64189601
C	-1.41135824	0.07254007	0.58335543
C	1.41135824	-0.07254007	0.58335543
C	-0.70560551	-0.01840045	1.76601207
C	0.70560551	0.01840045	1.76601207
C	-1.20537925	-0.24495600	3.11624599
C	1.20537925	0.24495600	3.11624599
H	-1.23801422	0.09516236	-4.00791264
H	1.23801422	-0.09516236	-4.00791264
H	-2.46452165	0.18680932	-1.89421546
H	2.46452165	-0.18680932	-1.89421546
H	-2.48556995	0.09077634	0.57873887
H	2.48556995	-0.09077634	0.57873887
H	-2.09696031	0.23605144	3.47871470
H	2.09696031	-0.23605144	3.47871470
H	-0.93970686	-1.16469932	3.59515071
H	0.93970686	1.16469932	3.59515071

CASSCF Energy = -460.435816885
NEVPT2 Energy = -462.362662338
NIMAG = 1
CASSCF H = -460.242991662
CASSCF G (HO) = -460.286105842
CASSCF G (GR) = -460.286019983

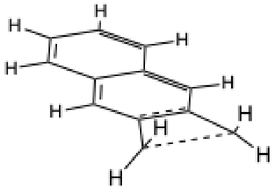

First-Order Saddle Point for Disrotatory

Ring Opening of Cyclobuta[b]naphthalene

CASSCF(12,12) Optimized Geometry

C	-3.08526754	0.00372421	-0.70619899
C	-3.08526754	0.00372421	0.70619899
C	-1.89775360	-0.00040371	-1.39388001
C	-1.89775360	-0.00040371	1.39388001
C	-0.65913892	-0.01063880	-0.70544654
C	-0.65913892	-0.01063880	0.70544654
C	0.59056777	-0.00409021	-1.40239108
C	0.59056777	-0.00409021	1.40239108
C	1.76788485	-0.02758634	-0.71355891
C	1.76788485	-0.02758634	0.71355891
C	3.11778998	0.03419228	-1.31267393
C	3.11778998	0.03419228	1.31267393
H	-4.01681137	0.00824076	-1.23899090
H	-4.01681137	0.00824076	1.23899090
H	-1.89613175	0.00263990	-2.46786666
H	-1.89613175	0.00263990	2.46786666
H	0.58687508	0.04008704	-2.47608447
H	0.58687508	0.04008704	2.47608447
H	3.31291366	0.70522177	-2.12783360
H	3.31291366	0.70522177	2.12783360
H	3.77245593	-0.81062520	-1.22405803
H	3.77245593	-0.81062520	1.22405803

CASSCF Energy = -460.428804175
NEVPT2 Energy = -462.345167306
NIMAG = 1
CASSCF H = -460.237046380
CASSCF G (HO) = -460.282254860
CASSCF G (GR) = -460.281521555

First-Order Saddle Point for Internal Rotation of Methylene in 2,3-Dimethylene-2,3-dihydronaphthalene

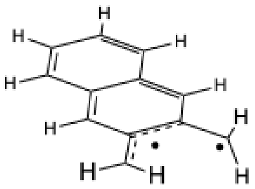

CASSCF(12,12) Optimized Geometry

C	-3.05614223	-1.52032530	0.00000000
C	-3.01185967	1.43084198	0.00000000
C	-1.79195971	-0.74603476	0.00000000
C	-1.81443648	0.71209388	0.00000000
C	-0.59499479	-1.39577843	0.00000000
C	-0.57998936	1.38915215	0.00000000
C	0.66121902	-0.70416352	0.00000000
C	0.65674012	0.71020014	0.00000000
C	1.89318697	-1.39182817	0.00000000
C	1.89954640	1.40072723	0.00000000
C	3.08456835	-0.70258511	0.00000000
C	3.08490848	0.71057066	0.00000000
H	-3.96586761	0.94686244	0.00000000
H	-3.59295677	-1.67541399	-0.91634560
H	-3.59295677	-1.67541399	0.91634560
H	-3.00090629	2.50270817	0.00000000
H	-0.58127778	-2.46979998	0.00000000
H	-0.58164286	2.46304215	0.00000000
H	1.89226359	-2.46576849	0.00000000
H	1.89837502	2.47455926	0.00000000

H	4.01577204	-1.23558076	0.00000000
H	4.01730466	1.24188946	0.00000000

CASSCF Energy = -460.440718898
NEVPT2 Energy = -462.357566804
NIMAG = 1
CASSCF H = -460.249386978
CASSCF G (HO) = -460.293270553
CASSCF G (GR) = -460.293126918

Data for main text Scheme 6.
Reaction E

(Z)-1,3,5-Hexatriene C_2 conformation

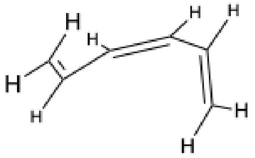

CASSCF(6,6) Optimized Geometry

C	-0.65093774	-0.16128159	-1.12498856
C	0.65093774	0.16128159	-1.12498856
C	-1.56153905	-0.34649995	0.02648215
C	1.56153905	0.34649995	0.02648215
C	-1.73440325	0.51366121	1.03336835
C	1.73440325	-0.51366121	1.03336835
H	-1.11480439	-0.29922909	-2.08718204
H	1.11480439	0.29922909	-2.08718204
H	-2.16677117	-1.23718369	-0.00190451
H	2.16677117	1.23718369	-0.00190451
H	-1.17813265	1.42937386	1.09852970
H	1.17813265	-1.42937386	1.09852970
H	-2.44037485	0.31314224	1.81689787
H	2.44037485	-0.31314224	1.81689787

CASSCF Energy = -231.956938147
NEVPT2 Energy = -232.938678677
NIMAG = 0
CASSCF H = -231.826505917
CASSCF G (HO) = -231.864344160
CASSCF G (GR) = -231.863985980

M06-2X Optimized Geometry

C	0.01578611	0.66706797	1.13201677
C	-0.01578611	-0.66706797	1.13201677
C	-0.01578611	1.58476494	-0.01782076
C	0.01578611	-1.58476494	-0.01782076
C	-0.85019815	1.49959733	-1.04492778
C	0.85019815	-1.49959733	-1.04492778
H	0.06271055	1.15564559	2.10054475
H	-0.06271055	-1.15564559	2.10054475
H	0.66694768	2.42761536	0.03484459
H	-0.66694768	-2.42761536	0.03484459
H	-1.56948855	0.69426872	-1.11986772
H	1.56948855	-0.69426872	-1.11986772
H	-0.83631988	2.23216361	-1.84028658
H	0.83631988	-2.23216361	-1.84028658

M06-2X Energy = -233.348972573
NIMAG = 0
M06-2X H = -233.222483389

M06-2X G (HO) = -233.260768261
M06-2X G (GR) = -233.260181260

ωB97X-D Optimized Geometry

C	0.01441672	0.66682518	1.12680059
C	-0.01441672	-0.66682518	1.12680059
C	-0.01441672	1.59024299	-0.01653230
C	0.01441672	-1.59024299	-0.01653230
C	-0.85283216	1.52971163	-1.04168145
C	0.85283216	-1.52971163	-1.04168145
H	0.06120143	1.15370662	2.09618922
H	-0.06120143	-1.15370662	2.09618922
H	0.68138066	2.42225389	0.04009449
H	-0.68138066	-2.42225389	0.04009449
H	-1.58674102	0.73891191	-1.12572088
H	1.58674102	-0.73891191	-1.12572088
H	-0.82977601	2.27257527	-1.82721458
H	0.82977601	-2.27257527	-1.82721458

ωB97X-D Energy = -233.377884516
NIMAG = 0
ωB97X-D H = -233.250298221
ωB97X-D G (HO) = -233.287384542
ωB97X-D G (GR) = -233.287286185

PBE0-D3BJ Optimized Geometry

C	0.01561474	0.66894093	1.12905379
C	-0.01561474	-0.66894093	1.12905379
C	-0.01561474	1.60506599	0.00553143
C	0.01561474	-1.60506599	0.00553143
C	-0.79735588	1.52521849	-1.06580684
C	0.79735588	-1.52521849	-1.06580684
H	0.06625841	1.14678425	2.10489333
H	-0.06625841	-1.14678425	2.10489333
H	0.62052433	2.48072549	0.11736847
H	-0.62052433	-2.48072549	0.11736847
H	-1.47620224	0.69393026	-1.21151541
H	1.47620224	-0.69393026	-1.21151541
H	-0.77733780	2.29248957	-1.82967880
H	0.77733780	-2.29248957	-1.82967880

PBE0-D3BJ Energy = -233.173987399
NIMAG = 0
PBE0-D3BJ H = -233.047483746
PBE0-D3BJ G (HO) = -233.085496765
PBE0-D3BJ G (GR) = -233.085081098

(Z)-1,3,5-Hexatriene C_{2v} conformation

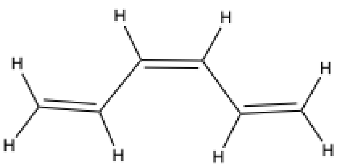

CASSCF(6,6) Optimized Geometry

C	-0.67381054	-0.01713595	-0.75486577
C	0.67381054	0.01713595	-0.75486577
C	-1.55333340	-0.03950074	0.41121214
C	1.55333340	0.03950074	0.41121214
C	-2.89044571	-0.07319111	0.32275399
C	2.89044571	0.07319111	0.32275399
H	-1.10351050	-0.02800602	1.38610190
H	1.10351050	0.02800602	1.38610190

H	-1.16981208	-0.02985657	-1.70927066
H	1.16981208	0.02985657	-1.70927066
H	-3.50838876	-0.08866983	1.19954913
H	3.50838876	0.08866983	1.19954913
H	-3.39200234	-0.08571823	-0.62753177
H	3.39200234	0.08571823	-0.62753177

CASSCF Energy = -231.969658793
NEVPT2 Energy = -232.954612190
NIMAG = 0
CASSCF H = -231.838478712
CASSCF G (HO) = -231.880407752
CASSCF G (GR) = -231.879423035

Cyclohexa-1,3-diene

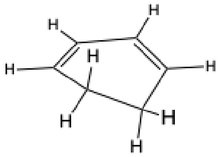

CASSCF(6,6) Optimized Geometry

C	-0.72626354	0.11654629	1.23252641
C	0.72626354	-0.11654629	1.23252641
C	-1.42313268	0.09512160	0.08841224
C	1.42313268	-0.09512160	0.08841224
C	-0.74818368	-0.21952402	-1.22214403
C	0.74818368	0.21952402	-1.22214403
H	-1.21849034	0.29414685	2.17029962
H	1.21849034	-0.29414685	2.17029962
H	-2.48884112	0.23139824	0.09756048
H	2.48884112	-0.23139824	0.09756048
H	-0.80726591	-1.29158181	-1.39937465
H	0.80726591	1.29158181	-1.39937465
H	-1.26453852	0.26022570	-2.04481607
H	1.26453852	-0.26022570	-2.04481607

CASSCF Energy = -231.980997054
NEVPT2 Energy = -232.974078974
NIMAG = 0
CASSCF H = -231.847211972
CASSCF G (HO) = -231.880807373
CASSCF G (GR) = -231.880810926

M06-2X Optimized Geometry

C	0.05514133	0.73108493	1.22201896
C	-0.05514133	-0.73108493	1.22201896
C	-0.05514133	1.41501275	0.08364256
C	0.05514133	-1.41501275	0.08364256
C	-0.32168081	0.69251234	-1.20958757
C	0.32168081	-0.69251234	-1.20958757
H	0.20423417	1.24288643	2.16377834
H	-0.20423417	-1.24288643	2.16377834
H	-0.01246461	2.49676762	0.08263787
H	0.01246461	-2.49676762	0.08263787
H	-1.40777160	0.59119496	-1.33053116
H	1.40777160	-0.59119496	-1.33053116
H	0.03264305	1.27453286	-2.05982104
H	-0.03264305	-1.27453286	-2.05982104

M06-2X Energy = -233.389027765
NIMAG = 0

M06-2X H = -233.259860597
M06-2X G (HO) = -233.293515301
M06-2X G (GR) = -233.293519845

ωB97X-D Optimized Geometry

C	0.05191075	0.73020261	1.22346651
C	-0.05191075	-0.73020261	1.22346651
C	-0.05191075	1.41473014	0.08566500
C	0.05191075	-1.41473014	0.08566500
C	-0.30821440	0.69930305	-1.21198951
C	0.30821440	-0.69930305	-1.21198951
H	0.19331729	1.24203948	2.16636683
H	-0.19331729	-1.24203948	2.16636683
H	-0.00943400	2.49649845	0.08642266
H	0.00943400	-2.49649845	0.08642266
H	-1.39357318	0.62184613	-1.35478694
H	1.39357318	-0.62184613	-1.35478694
H	0.07173685	1.27682808	-2.05465562
H	-0.07173685	-1.27682808	-2.05465562

ωB97X-D Energy = -233.419054623
NIMAG = 0
ωB97X-D H = -233.289558364
ωB97X-D G (HO) = -233.323173961
ωB97X-D G (GR) = -233.323178446

PBE0-D3BJ Optimized Geometry

C	0.05264978	0.72695300	1.22252992
C	-0.05264978	-0.72695300	1.22252992
C	-0.05264978	1.41327605	0.08237274
C	0.05264978	-1.41327605	0.08237274
C	-0.31117255	0.69553841	-1.20811358
C	0.31117255	-0.69553841	-1.20811358
H	0.19344749	1.24179626	2.16599789
H	-0.19344749	-1.24179626	2.16599789
H	-0.01458711	2.49684996	0.08364334
H	0.01458711	-2.49684996	0.08364334
H	-1.39954667	0.61267878	-1.34517542
H	1.39954667	-0.61267878	-1.34517542
H	0.05829016	1.27453492	-2.05691676
H	-0.05829016	-1.27453492	-2.05691676

PBE0-D3BJ Energy = -233.219684599
NIMAG = 0
PBE0-D3BJ H = -233.090956633
PBE0-D3BJ G (HO) = -233.124641868
PBE0-D3BJ G (GR) = -233.124646053

First-Order Saddle Point for Disrotatory Ring Closure of (Z)-1,3,5-Hexatriene

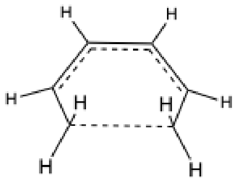

CASSCF(6,6) Optimized Geometry

C	-1.23434329	-0.05981619	-1.14155853
C	-1.23434329	-0.05981619	1.14155853
C	0.10983559	0.17382605	-1.49438524
C	0.10983559	0.17382605	1.49438524
C	1.21142817	-0.12708642	-0.70397913
C	1.21142817	-0.12708642	0.70397913

H	-1.98859441	0.51130998	-1.65770292
H	-1.98859441	0.51130998	1.65770292
H	-1.53004706	-1.06358290	-0.94497365
H	-1.53004706	-1.06358290	0.94497365
H	0.30547366	0.76167899	-2.37542200
H	0.30547366	0.76167899	2.37542200
H	2.17959785	-0.05391282	-1.16629851
H	2.17959785	-0.05391282	1.16629851

CASSCF Energy = -231.897238130
NEVPT2 Energy = -232.904996138
NIMAG = 1
CASSCF H = -231.767265497
CASSCF G (HO) = -231.802476540
CASSCF G (GR) = -231.802342086

M06-2X Optimized Geometry

C	-0.10769861	-1.21715927	1.12047669
C	-0.10769861	-1.21715927	-1.12047669
C	0.20145768	0.09142370	1.46261038
C	0.20145768	0.09142370	-1.46261038
C	-0.10769861	1.21252935	0.69835245
C	-0.10769861	1.21252935	-0.69835245
H	0.44150671	-2.01847875	1.60334645
H	0.44150671	-2.01847875	-1.60334645
H	-1.13207352	-1.45990157	0.91488806
H	-1.13207352	-1.45990157	-0.91488806
H	0.86972706	0.26054899	2.30229047
H	0.86972706	0.26054899	-2.30229047
H	-0.01318453	2.18439263	1.16882430
H	-0.01318453	2.18439263	-1.16882430

M06-2X Energy = -233.313351194
NIMAG = 1
M06-2X H = -233.187035993
M06-2X G (HO) = -233.222504985
M06-2X G (GR) = -233.222158219

ωB97X-D Optimized Geometry

C	-0.10605026	-1.21744658	1.12498128
C	-0.10605026	-1.21744658	-1.12498128
C	0.19837451	0.09264097	1.46460116
C	0.19837451	0.09264097	-1.46460116
C	-0.10605026	1.21152755	0.69796744
C	-0.10605026	1.21152755	-0.69796744
H	0.44205479	-2.01604364	1.61405485
H	0.44205479	-2.01604364	-1.61405485
H	-1.12950425	-1.46378410	0.91884664
H	-1.12950425	-1.46378410	-0.91884664
H	0.86100390	0.26414813	2.30826397
H	0.86100390	0.26414813	-2.30826397
H	-0.01012125	2.18309629	1.16890685
H	-0.01012125	2.18309629	-1.16890685

ωB97X-D Energy = -233.341109049
NIMAG = 1
ωB97X-D H = -233.214737797
ωB97X-D G (HO) = -233.249904718
ωB97X-D G (GR) = -233.249758573

PBE0-D3BJ Optimized Geometry

C	-0.10602123	-1.21780405	1.13247577
C	-0.10602123	-1.21780405	-1.13247577
C	0.19789337	0.09261466	1.46502803
C	0.19789337	0.09261466	-1.46502803
C	-0.10602123	1.21207565	0.69748697
C	-0.10602123	1.21207565	-0.69748697
H	0.44321488	-2.01609516	1.62414809
H	0.44321488	-2.01609516	-1.62414809
H	-1.12651477	-1.46967935	0.91098408
H	-1.12651477	-1.46967935	-0.91098408
H	0.86428908	0.26583847	2.30819340
H	0.86428908	0.26583847	-2.30819340
H	-0.01251832	2.18539619	1.16933684
H	-0.01251832	2.18539619	-1.16933684

PBE0-D3BJ Energy = -233.145232320
NIMAG = 1
PBE0-D3BJ H = -233.019575259
PBE0-D3BJ G (HO) = -233.054967315
PBE0-D3BJ G (GR) = -233.054729831

First-Order Saddle Point for Conrotatory Ring Closure of (Z)-1,3,5-Hexatriene

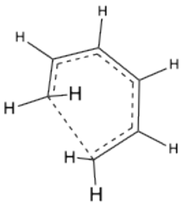

CASSCF(6,6) Optimized Geometry

C	1.17022347	-0.48113438	1.15333509
C	-1.17022347	0.48113438	1.15333509
C	1.50509536	0.19479851	-0.07168052
C	-1.50509536	-0.19479851	-0.07168052
C	0.68311310	0.25868133	-1.16403544
C	-0.68311310	-0.25868133	-1.16403544
H	1.77125764	-0.30921894	2.02572203
H	-1.77125764	0.30921894	2.02572203
H	0.61278403	-1.39320314	1.12975764
H	-0.61278403	1.39320314	1.12975764
H	2.44317603	0.71930230	-0.11136726
H	-2.44317603	-0.71930230	-0.11136726
H	1.05054605	0.71537554	-2.06452298
H	-1.05054605	-0.71537554	-2.06452298

CASSCF Energy = -231.880040966
NEVPT2 Energy = -232.867486508
NIMAG = 1
CASSCF H = -231.754488175
CASSCF G (HO) = -231.789497071
CASSCF G (GR) = -231.789505441

UM06-2X Optimized Geometry

C	-0.40478413	1.11617879	-1.14553362
C	0.40478413	-1.11617879	-1.14553362
C	0.28313083	1.45565503	0.05554383
C	-0.28313083	-1.45565503	0.05554383
C	0.28313083	0.66388705	1.17311323
C	-0.28313083	-0.66388705	1.17311323
H	-0.18777147	1.65260233	-2.05809635
H	0.18777147	-1.65260233	-2.05809635
H	-1.35851275	0.61259213	-1.09365036

H 1.35851275 -0.61259213 -1.09365036
H 0.85835949 2.37462886 0.07226364
H -0.85835949 -2.37462886 0.07226364
H 0.71574475 1.04511300 2.08974651
H -0.71574475 -1.04511300 2.08974651

UM06-2X Energy = -233.276741680
NIMAG = 1
<S^2> = 1.0032
UM06-2X H = -233.155073450
UM06-2X G (HO) = -233.189812685
UM06-2X G (GR) = -233.189821239

<u>Second-Order Saddle Point for Terminal Methylene Internal Rotation of (Z)-1,3,5-Hexatriene</u>

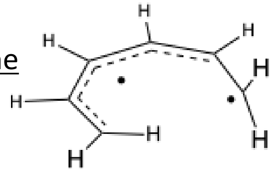

CASSCF(6,6) Optimized Geometry
C -0.81854093 -1.07538939 0.00000000
C -1.70600295 -0.03811785 0.00000000
C -1.47962058 1.41585088 0.00000000
C 0.60889095 -1.19561136 0.00000000
C 1.70710957 -0.28320712 0.00000000
C 1.75800455 1.07610500 0.00000000
H -1.29494488 -2.04057741 0.00000000
H -2.74366164 -0.33349109 0.00000000
H -1.55407739 1.97022831 -0.91658366
H -1.55407739 1.97022831 0.91658366
H 2.71224999 1.56649232 0.00000000
H 0.89135396 1.69871843 0.00000000
H 2.66046333 -0.78344733 0.00000000
H 0.92567533 -2.22233772 0.00000000

CASSCF Energy = -231.869747918
NEVPT2 Energy = -232.851639719
NIMAG = 2
CASSCF H = -231.745039536
CASSCF G (HO) = -231.781141785
CASSCF G (GR) = -231.781150098

Reaction F

<u>3,4-Divinyl-1,3,5-Hexatriene</u>

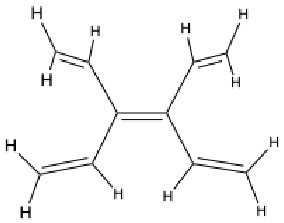

CASSCF(10,10) Optimized Geometry
C -0.68179333 0.00000000 0.00000000
C 0.68179333 0.00000000 0.00000000
C -1.43470728 -1.27629542 0.05706059
C 1.43470728 -1.27629542 -0.05706059
C -1.43470728 1.27629542 -0.05706059
C 1.43470728 1.27629542 0.05706059
C -2.61462522 -1.52476776 -0.52460575
C 2.61462522 -1.52476776 0.52460575

C	-2.61462522	1.52476776	0.52460575
C	2.61462522	1.52476776	-0.52460575
H	-0.97092140	-2.07464623	0.60482168
H	0.97092140	-2.07464623	-0.60482168
H	-0.97092140	2.07464623	-0.60482168
H	0.97092140	2.07464623	0.60482168
H	-3.07135081	-2.49207902	-0.43594608
H	3.07135081	-2.49207902	0.43594608
H	-3.07135081	2.49207902	0.43594608
H	3.07135081	2.49207902	-0.43594608
H	-3.14179707	-0.78820682	-1.09876275
H	3.14179707	-0.78820682	1.09876275
H	-3.14179707	0.78820682	1.09876275
H	3.14179707	0.78820682	-1.09876275

CASSCF Energy = -385.825523973
NEVPT2 Energy = -387.460962231
NIMAG = 0
CASSCF H = -385.621443112
CASSCF G (HO) = -385.670117750
CASSCF G (GR) = -385.668547192

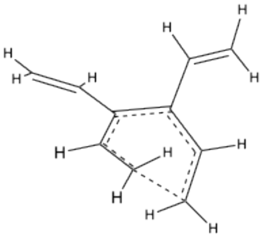

First-order Saddle Point for Conrotatory Ring closure of 3,4-Divinyl-1,3,5-Hexatriene

CASSCF(10,10) Optimized Geometry

C	0.87087313	0.88238729	2.17927209
C	-0.87089283	-0.88231555	2.17928856
C	0.32132902	1.45424601	0.98893595
C	-0.32147702	-1.45426337	0.98894366
C	-0.03605377	0.74078160	-0.13470761
C	0.03628594	-0.74076271	-0.13470877
C	-0.29668879	2.66612382	-1.72536546
C	0.29682209	-2.66621808	-1.72529597
C	-0.54399144	1.40313685	-1.34527005
C	0.54390691	-1.40309019	-1.34533659
H	0.93767808	1.49272964	3.05973639
H	-0.93806975	-1.49295912	3.05968814
H	-1.53523811	-0.04644256	2.13103618
H	1.53550138	0.04691758	2.13095801
H	0.07499703	2.49944198	1.01897681
H	-0.07601861	-2.49990740	1.01887905
H	0.35007153	3.31814227	-1.16988561
H	-0.34937179	-3.31862083	-1.16954135
H	-0.73845895	3.06605361	-2.61769525
H	0.73819215	-3.06593851	-2.61781929
H	-1.16725877	0.79832063	-1.97995782
H	1.16662777	-0.79804293	-1.98036230

CASSCF Energy = -385.751795014
NEVPT2 Energy = -387.391450201
NIMAG = 1
CASSCF H = -385.552750508
CASSCF G (HO) = -385.599404217
CASSCF G (GR) = -385.598623347

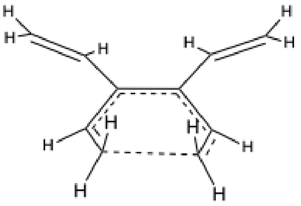

First-order Saddle Point for Disrotatory

Ring closure of 3,4-Divinyl-1,3,5-Hexatriene

CASSCF(10,10) Optimized Geometry

C	-1.55216088	0.82191276	0.28125357
C	-0.69121403	-0.26652344	0.07570736
C	0.72500517	-0.11874634	0.04081710
C	1.60201756	-1.31378169	0.09775090
C	2.85663339	-1.33866470	-0.36890498
C	1.35355005	1.12060260	0.24663966
C	0.87144135	2.38380747	-0.13372828
C	-1.30061223	-1.61404230	0.20905968
C	-2.51877746	-1.92537974	-0.24930793
C	-1.33699496	2.14027111	-0.14848444
H	3.45834753	-2.22333321	-0.28150918
H	3.30001864	-0.49344955	-0.85993350
H	1.22083128	-2.21287722	0.53818357
H	2.21916930	1.09174829	0.88473032
H	0.69013235	2.56136046	-1.16838145
H	1.28514205	3.23952285	0.37509597
H	-2.92979195	-2.90585837	-0.09991445
H	-3.11860711	-1.22198084	-0.79486457
H	-0.75077199	-2.38059649	0.71779765
H	-1.18970925	2.30540942	-1.19081155
H	-1.91343549	2.91626826	0.32774053
H	-2.37715266	0.64001810	0.94696825

CASSCF Energy = -385.760743574
NEVPT2 Energy = -387.420634500
NIMAG = 1
CASSCF H = -385.557779451
CASSCF G (HO) = -385.604727015
CASSCF G (GR) = -385.603415833

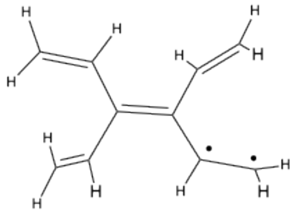

First-order Saddle Point for CH_2 Internal Rotation of 3,4-Divinyl-1,3,5-Hexatriene

CASSCF(10,10) Optimized Geometry

C	0.72843087	-0.08161557	0.13107991
C	-0.72798505	0.01041458	0.11438077
C	-1.51321709	-1.22952390	0.26552838
C	-2.57430906	-1.59100322	-0.46780632
C	-1.35610841	1.28332273	-0.02059355
C	-2.68140696	1.58074604	0.11640101
C	1.36391025	-1.27815672	-0.00705681
C	2.81860021	-1.51374531	0.08057676
C	1.50534398	1.17337392	0.34207417
C	2.43663535	1.65284979	-0.48517468
H	-3.07799652	-2.52043560	-0.28100224
H	-2.94993422	-0.98489760	-1.26949201
H	-1.17082902	-1.90960857	1.02553987
H	-3.01668268	2.59182145	-0.00764536
H	-3.41867299	0.84816082	0.37179779
H	-0.70427887	2.10719351	-0.24032599
H	3.40884483	-1.61198781	-0.81019571
H	3.24383417	-1.89159876	0.99129691
H	0.76144071	-2.15277361	-0.17888004
H	2.96207871	2.56181159	-0.26043706
H	2.69249640	1.15524493	-1.40158830

H 1.27096043 1.72774246 1.23448345

CASSCF Energy = -385.743138224
NEVPT2 Energy = -387.373891548
NIMAG = 1
CASSCF H = -385.544565230
CASSCF G (HO) = -385.593234950
CASSCF G (GR) = -385.592216079

Reaction G

7-Vinylbicyclo[4.2.0]octa-1,3,5-triene **4**

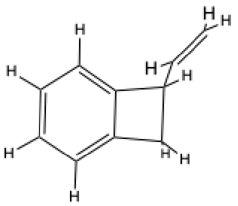

CASSCF(10,10) Optimized Geometry

C	-1.34559652	0.23513826	-0.45188309
C	-2.39776002	-0.21172764	0.51286211
C	-3.60782808	-0.65625200	0.16726887
C	0.06467436	-0.29987442	-0.25092125
C	0.62724041	0.94386889	-0.03332555
C	-0.68397137	1.68792514	-0.17543890
C	1.97995930	1.11273708	0.20875647
C	2.74914655	-0.05568923	0.22579038
C	2.17817510	-1.31453704	0.01023602
C	0.80952090	-1.46688889	-0.23576434
H	-2.13497411	-0.16065179	1.55672947
H	-3.91054971	-0.72639869	-0.86186755
H	-4.32421073	-0.96230398	0.90594623
H	-0.75750565	2.37491293	-1.00845567
H	-1.04897912	2.17238336	0.72099626
H	0.38288758	-2.43815012	-0.39958362
H	2.81121829	-2.18143639	0.03506846
H	3.80497731	0.00910429	0.41011883
H	2.43196044	2.07165152	0.37784456
H	-1.70274682	0.14212984	-1.46985445

CASSCF Energy = -384.686073879
NEVPT2 Energy = -386.310486332
NIMAG = 0
CASSCF H = -384.508294141
CASSCF G (HO) = -384.550818740
CASSCF G (GR) = -384.550356214

First-Order Saddle Point for Outward Conrotatory Ring Opening of 7-Vinylbicyclo[4.2.0]octa-1,3,5-triene

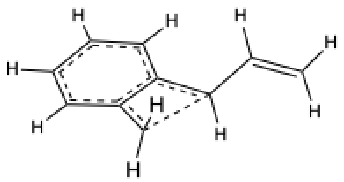

CASSCF(10,10) Optimized Geometry

C	-2.12890188	0.95495781	0.05912088
C	-2.76184549	-0.27812537	0.02294287
C	-2.01701336	-1.46655075	-0.03292347
C	-0.63247076	-1.43293066	-0.10359821
C	0.00130102	-0.18488641	-0.11488873
C	-0.72944955	0.98385218	0.04464854
C	0.18415115	2.10951647	0.25003893

C	1.41175095	0.13606550	-0.37220716
C	2.51216922	-0.50669957	0.29506764
C	3.80650401	-0.38680244	-0.07430562
H	0.73565124	2.14055317	1.16812918
H	0.02461791	3.05979588	-0.23044635
H	4.09435660	0.20069699	-0.92650699
H	4.59129272	-0.86556481	0.47815143
H	2.27734678	-1.09682369	1.16450125
H	1.62052232	0.63399668	-1.29854361
H	-0.06773506	-2.34173770	-0.19413288
H	-2.53009901	-2.40928092	-0.04794895
H	-3.83342550	-0.33004239	0.05866349
H	-2.69983587	1.86097432	0.13895322

CASSCF Energy = -384.637524626
NEVPT2 Energy = -386.258943540
NIMAG = 1
CASSCF H = -384.462444515
CASSCF G (HO) = -384.505095829
CASSCF G (GR) = -384.504575463

First-Order Saddle Point for Inward Conrotatory Ring Opening of 7-Vinylbicyclo[4.2.0]octa-1,3,5-triene

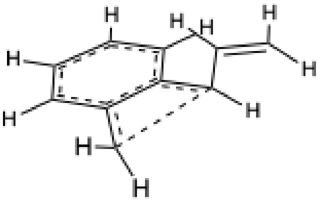

CASSCF(10,10) Optimized Geometry

C	2.35054839	-0.28505212	-0.56112036
C	1.41069707	-0.43990787	0.50633658
C	-0.05277190	-0.35275504	0.28922700
C	-0.92627173	-1.42436292	0.08858605
C	-2.24886240	-1.15884626	-0.24155156
C	-2.69698201	0.16075837	-0.36873857
C	-1.83717951	1.22363457	-0.12818928
C	-0.51553517	0.95375359	0.24117612
C	0.50609596	1.91573685	0.68097181
C	3.65685965	-0.63858342	-0.48528147
H	0.78818457	1.91597550	1.71504831
H	0.69041182	2.82513247	0.13789347
H	-2.18967319	2.23579838	-0.19645282
H	-3.71847111	0.34971802	-0.63899445
H	-2.92859586	-1.96971203	-0.42171818
H	-0.57375924	-2.43698729	0.15037383
H	1.76376839	-0.96014675	1.38213578
H	4.07227089	-1.06720139	0.40786615
H	4.32115349	-0.49265424	-1.31424319
H	1.98260307	0.14331742	-1.47690934

CASSCF Energy = -384.632399241
NEVPT2 Energy = -386.249992144
NIMAG = 1
CASSCF H = -384.454244204
CASSCF G (HO) = -384.497389900
CASSCF G (GR) = -384.497004289

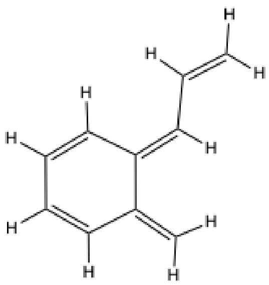

(*E*)-5-Allylidene-6-methylenecyclohexa-1,3-diene **5**

CASSCF(10,10) Optimized Geometry

C	0.26890971	-1.37656653	0.17874086
C	1.55895258	-1.75053860	0.07544590
C	2.60524771	-0.76448940	-0.14842267
C	2.30314178	0.54543736	-0.18448718
C	0.93623072	1.03659060	0.01065379
C	-0.15036242	0.02056258	0.05827565
C	-1.46090345	0.37532235	-0.04805964
C	-2.62111368	-0.50094465	0.00671916
C	-3.88066620	-0.05319420	-0.12279206
C	0.74158651	2.36432273	0.17541601
H	-4.71779537	-0.72228204	-0.07641161
H	-4.09660595	0.98816899	-0.27715133
H	-2.47109618	-1.55213696	0.15854861
H	-1.69440159	1.41115298	-0.20301155
H	-0.21529731	2.79497709	0.38753432
H	1.56961798	3.04369554	0.11274732
H	3.07560714	1.27792303	-0.32230077
H	3.61894807	-1.09456240	-0.26974045
H	1.82633949	-2.78721671	0.14745822
H	-0.47954873	-2.12738932	0.32458812

CASSCF Energy = -384.689989046
NEVPT2 Energy = -386.300080839
NIMAG = 0
CASSCF H = -384.511538147
CASSCF G (HO) = -384.555723416
CASSCF G (GR) = -384.555089256

First-Order Saddle Point for Interconversion of (*E*) and (Z)-5-Allylidene-6-methylenecyclohexa-1,3-diene **27**

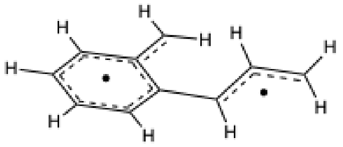

CASSCF(10,10) Optimized Geometry

C	-0.65407753	-1.43373072	-0.19571273
C	-2.02908158	-1.47730422	0.04790131
C	-2.73364186	-0.28486189	0.23691800
C	-2.06986594	0.92315453	0.18088700
C	-0.66981077	0.99669468	-0.06596559
C	0.04076589	-0.23255521	-0.25214493
C	1.50150335	-0.24737057	-0.52879047
C	2.47331572	-0.27316490	0.46132773
C	3.83816695	-0.29995590	0.22251894
C	-0.03782547	2.25016332	-0.12236705
H	4.54208851	-0.32282150	1.02986884
H	4.22854662	-0.30336466	-0.77728397
H	2.14216471	-0.27331871	1.48560357
H	1.81300604	-0.24027653	-1.55906725
H	1.01152384	2.34868193	-0.30290624
H	-0.60612762	3.14784908	0.01840924
H	-2.61262298	1.83816767	0.32507539

H	-3.79008126	-0.30762109	0.42492598
H	-2.53692985	-2.42113566	0.08996850
H	-0.11603190	-2.35146856	-0.34103134

CASSCF Energy = -384.646474194
NEVPT2 Energy = -386.261156439
NIMAG = 1
CASSCF H = -384.469287858
CASSCF G (HO) = -384.511793252
CASSCF G (GR) = -384.511543789

UM06-2X Optimized Geometry

C	-0.65406675	-1.43841338	-0.19694106
C	-2.02217264	-1.47486438	0.05339295
C	-2.72093198	-0.28451654	0.23997527
C	-2.05689807	0.91933628	0.17921460
C	-0.66444265	0.98620586	-0.07136744
C	0.03821286	-0.24007410	-0.26294426
C	1.49100966	-0.24295527	-0.53759220
C	2.45292631	-0.26958713	0.45690543
C	3.80806930	-0.28007996	0.22798338
C	-0.01415064	2.22998416	-0.12329624
H	4.51655257	-0.30182683	1.04159647
H	4.19992694	-0.26989169	-0.78021492
H	2.10232983	-0.28208407	1.48443770
H	1.81330719	-0.21812437	-1.57446089
H	1.04703486	2.30583606	-0.30062947
H	-0.57408026	3.14188511	0.02168728
H	-2.59623836	1.84663556	0.32636539
H	-3.78473586	-0.30490249	0.43424188
H	-2.53706933	-2.42420735	0.10310050
H	-0.10959846	-2.36259445	-0.34331943

UM06-2X Energy = -386.929542552
NIMAG = 1
$\langle S^2 \rangle$ = 1.0635
UM06-2X H = -386.757711405
UM06-2X G (HO) = -386.800605011
UM06-2X G (GR) = -386.800295440

(*Z*)-5-Allylidene-6-methylenecyclohexa-1,3-diene **6a**

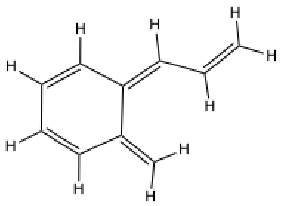

CASSCF(10,10) Optimized Geometry

C	0.92315330	-1.48749606	0.28508302
C	2.23166660	-1.28895128	0.03792646
C	2.71903565	0.03212824	-0.33657965
C	1.88982240	1.09106152	-0.32101319
C	0.48869842	0.96709733	0.10082425
C	-0.06262637	-0.41007731	0.16275406
C	-1.37425269	-0.75754431	0.05965865
C	-2.52047985	0.07275031	-0.28354497
C	-3.78784416	-0.36390012	-0.21131255
C	-0.17587288	2.07044250	0.50552179
H	-4.60920635	0.25918501	-0.50774254
H	-4.02741734	-1.34761271	0.14930562
H	-2.33587959	1.06132077	-0.65522181
H	-1.59962595	-1.80196682	0.18862413

H	-1.15385187	2.03222670	0.93671377
H	0.29170524	3.03507671	0.44804736
H	2.25436615	2.07408247	-0.55162176
H	3.75120873	0.15233855	-0.60402879
H	2.92453700	-2.10646562	0.09000274
H	0.55942666	-2.47125506	0.51404334

CASSCF Energy = -384.687532848
NEVPT2 Energy = -386.297961622
NIMAG = 0
CASSCF H = -384.510342476
CASSCF G (HO) = -384.553668602
CASSCF G (GR) = -384.553309653

First-Order Saddle Point for Disrotatory Ring Closure of (*Z*)-5-Allylidene-6-methylenecyclohexa-1,3-diene

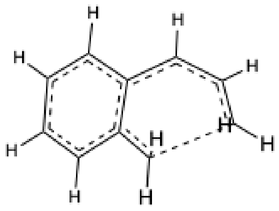

CASSCF(10,10) Optimized Geometry

C	-0.13040766	-0.79525889	0.16929035
C	-0.03965338	0.62263066	0.27574730
C	1.16788472	1.33748358	0.06769248
C	2.38357594	0.84047147	-0.39384731
C	2.95337139	-0.42905684	-0.20839688
C	-1.26288249	1.37254054	0.25280588
C	-2.45852926	0.77930926	-0.02558482
C	-2.50881172	-0.61000066	-0.32972722
C	-1.37328023	-1.36269079	-0.25681958
C	0.95886701	-1.63193785	0.48199768
H	1.03518708	2.39818122	-0.05235878
H	2.89142633	1.49194802	-1.08695058
H	3.12198827	-0.81620474	0.76620493
H	3.68586795	-0.74678160	-0.93186186
H	-1.41291392	-2.41924165	-0.44397828
H	-3.44447430	-1.06564440	-0.59114650
H	-3.35756727	1.36442122	-0.05627810
H	-1.21365711	2.43426182	0.40519492
H	1.47869620	-1.44113644	1.39110508
H	0.90496448	-2.67018343	0.20526395

CASSCF Energy = -384.639599963
NEVPT2 Energy = -386.275281590
NIMAG = 1
CASSCF H = -384.458718826
CASSCF G (HO) = -384.499835690
CASSCF G (GR) = -384.499668822

M06-2X Optimized Geometry

C	0.10625192	0.79470041	0.18996039
C	0.04722313	-0.63397351	0.32214323
C	-1.13675603	-1.34350672	0.10579530
C	-2.32350750	-0.82710113	-0.43782976
C	-2.92905816	0.39210376	-0.25765454
C	1.28846103	-1.35174565	0.31899802
C	2.44838644	-0.73823127	-0.02457905
C	2.45064112	0.63529399	-0.40765540
C	1.31164071	1.36763244	-0.32945687
C	-0.96016627	1.58456665	0.56744164
H	-1.00672124	-2.41731731	0.01532749

H	-2.74600951	-1.45762618	-1.21837789
H	-3.08348808	0.82926588	0.70695848
H	-3.63054778	0.72654062	-1.01470877
H	1.31591770	2.42550303	-0.56104384
H	3.37355772	1.09657549	-0.73172198
H	3.37224288	-1.29981303	-0.05166765
H	1.26835940	-2.41420630	0.52768950
H	-1.50469247	1.30432218	1.44938207
H	-0.96777330	2.63868300	0.31660126

M06-2X Energy = -386.939860704
NIMAG = 1
M06-2X H = -386.764031440
M06-2X G (HO) = -386.805257708
M06-2X G (GR) = -386.805116483

First-Order Saddle Point for Conrotatory Ring Closure of (Z)-5-Allylidene-6-methylenecyclohexa-1,3-diene

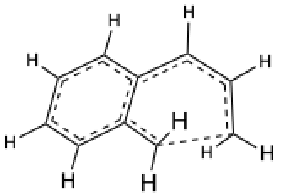

CASSCF(10,10) Optimized Geometry

C	0.91000069	1.70655024	-0.64254059
C	-0.17283928	0.81054255	-0.31126162
C	0.00877764	-0.60242123	-0.26571958
C	1.34334820	-1.20451877	-0.47274241
C	2.48790517	-0.72760553	0.09955209
C	2.54288350	0.36474647	1.01228692
C	-1.08382244	-1.41837888	0.00207846
C	-2.34791779	-0.88424009	0.26508733
C	-2.52106869	0.49366225	0.26805143
C	-1.44284165	1.32705318	-0.01521565
H	3.49280979	0.77633568	1.29232471
H	1.70461466	0.62029971	1.62322022
H	3.42078770	-1.17271674	-0.19773615
H	1.40166402	-2.06831517	-1.10858287
H	-1.58763673	2.39086202	-0.03410488
H	-3.48424791	0.91847825	0.47669615
H	-3.17376378	-1.53725906	0.47152976
H	-0.94615316	-2.48344066	0.02335797
H	0.75328736	2.76308646	-0.53810424
H	1.69985820	1.39544549	-1.28914709

CASSCF Energy = -384.638276522
NEVPT2 Energy = -386.259254090
NIMAG = 1
CASSCF H = -384.460556717
CASSCF G (HO) = -384.501919986
CASSCF G (GR) = -384.501819648

UM06-2X Optimized Geometry

C	0.94200188	1.67021717	-0.61394889
C	-0.15228019	0.79218088	-0.28459695
C	0.00963705	-0.62024774	-0.25723095
C	1.33435773	-1.22123143	-0.45688639
C	2.46871471	-0.69603654	0.08344432
C	2.48411060	0.41328892	0.95968655
C	-1.09647748	-1.42391150	-0.01605135
C	-2.34825017	-0.87745093	0.24510106
C	-2.50025443	0.50038221	0.27223083
C	-1.41305488	1.32060720	0.01017133

H	3.41655024	0.89964017	1.20541240
H	1.63217672	0.64223242	1.57990638
H	3.42084069	-1.12703621	-0.20542184
H	1.39361677	-2.12054813	-1.05725432
H	-1.54092470	2.39581154	0.00113702
H	-3.46599096	0.93860225	0.48457590
H	-3.19241967	-1.52563805	0.43550678
H	-0.96670065	-2.49912629	-0.01006975
H	0.83149187	2.73022349	-0.43374758
H	1.70400623	1.35901097	-1.30849120

UM06-2X Energy = -386.925618718
NIMAG = 1
<S^2> = 0.9049
UM06-2X H = -386.753160888
UM06-2X G (HO) = -386.794557096
UM06-2X G (GR) = -386.794467684

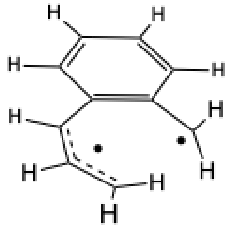

Second-Order Saddle Point for Internal Rotation of (Z)-5-Allylidene-6-methylenecyclohexa-1,3-diene

CASSCF(10,10) Optimized Geometry

C	1.09452387	1.43031237	0.00000000
C	0.01163766	0.51334313	0.00000000
C	0.34369215	-0.86711195	0.00000000
C	1.69062384	-1.24551299	0.00000000
C	2.72724851	-0.32223653	0.00000000
C	2.41750988	1.03491268	0.00000000
C	-0.64393578	-1.96842876	0.00000000
C	-1.29361753	1.15427992	0.00000000
C	-2.66685502	0.78658685	0.00000000
C	-3.35132400	-0.39624095	0.00000000
H	3.19618567	1.77328870	0.00000000
H	3.74725898	-0.65454653	0.00000000
H	1.92232903	-2.29399624	0.00000000
H	-0.92669741	-2.44305625	-0.91905171
H	-0.92669741	-2.44305625	0.91905171
H	-4.42376248	-0.36793488	0.00000000
H	-2.89561883	-1.35884598	0.00000000
H	-3.29941145	1.65813920	0.00000000
H	-1.18881110	2.22260270	0.00000000
H	0.87188186	2.47973237	0.00000000

CASSCF Energy = -384.619181857
NEVPT2 Energy = -386.235480741
NIMAG = 2
CASSCF H = -384.442725777
CASSCF G (HO) = -384.484516864
CASSCF G (GR) = -384.484452589

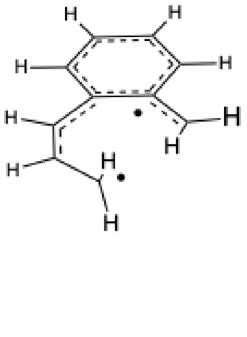

Second-Order Saddle Point for Internal Rotation of (Z)-5-Allylidene-6-methylenecyclohexa-1,3-diene

CASSCF(10,10) Optimized Geometry

C	1.38225598	1.10129057	0.00000000
C	2.67218715	0.70614676	0.00000000
C	3.34261662	-0.60499849	0.00000000

C	0.02517146	0.50446848	0.00000000
C	-0.37677562	-0.88117720	0.00000000
C	0.47810180	-1.98457839	0.00000000
C	-1.78147453	-1.16546883	0.00000000
C	-2.74314254	-0.18671979	0.00000000
C	-2.34842437	1.15723668	0.00000000
C	-0.99495285	1.46808074	0.00000000
H	3.74599368	-0.99421235	-0.91581380
H	3.74599368	-0.99421235	0.91581380
H	3.36736185	1.53306363	0.00000000
H	0.05793125	-2.97087254	0.00000000
H	1.53717393	-1.90694036	0.00000000
H	-0.71600693	2.50393949	0.00000000
H	-3.07810586	1.94320105	0.00000000
H	-3.78332036	-0.45029833	0.00000000
H	-2.07673543	-2.19724573	0.00000000
H	1.30086560	2.17285882	0.00000000

CASSCF Energy = -384.611819386
NEVPT2 Energy = -386.225228651
NIMAG = 2
CASSCF H = -384.435625238
CASSCF G (HO) = -384.477060969
CASSCF G (GR) = -384.476976618

1,2-Dihydronaphthalene **7**

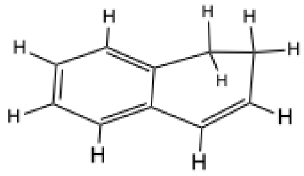

CASSCF(10,10) Optimized Geometry

C	-1.27472008	-1.40211610	-0.29351206
C	-2.45330239	-0.63240004	0.36877172
C	-2.37599717	0.83911032	0.05926348
C	-1.19974808	1.44388268	-0.14810664
C	0.07187428	0.70561694	-0.08791404
C	1.29304810	1.37180532	0.00625291
C	2.48631724	0.66145828	0.09586400
C	2.46278683	-0.72755063	0.09036191
C	1.24575336	-1.39938469	-0.01156208
C	0.04839152	-0.69892570	-0.10201522
H	1.23242880	-2.47377739	-0.02380609
H	3.37773301	-1.28404797	0.16237150
H	3.41932159	1.18648516	0.17165918
H	1.30903920	2.44597415	0.01593269
H	-1.15626587	2.50302436	-0.32412768
H	-3.28413271	1.41315432	0.06309532
H	-3.39204593	-1.04717553	0.02187721
H	-2.42645318	-0.77577998	1.44680083
H	-1.22810489	-2.41200560	0.09436891
H	-1.47600169	-1.47876133	-1.35912499

CASSCF Energy = -384.741668453
NEVPT2 Energy = -386.368694965
NIMAG = 0
CASSCF H = -384.561747286
CASSCF G (HO) = -384.601958955
CASSCF G (GR) = -384.601922402

Reaction H

<u>1,2-Divinylbenzene</u>

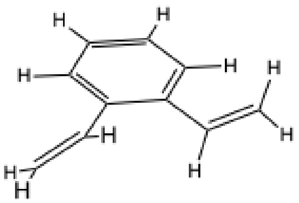

CASSCF(10,10) Optimized Geometry

C	-2.67729855	0.25639510	-1.64673105
C	2.67729855	-0.25639510	-1.64673105
C	-1.44871974	-0.22292414	-1.42540064
C	1.44871974	0.22292414	-1.42540064
C	-1.36749184	-0.19824675	1.06969902
C	1.36749184	0.19824675	1.06969902
C	-0.69748092	-0.09594178	-0.15307717
C	0.69748092	0.09594178	-0.15307717
C	-0.68776488	-0.10028079	2.27665219
C	0.68776488	0.10028079	2.27665219
H	-3.21650743	0.81523067	-0.90502867
H	3.21650743	-0.81523067	-0.90502867
H	-3.16488290	0.10682943	-2.59098473
H	3.16488290	-0.10682943	-2.59098473
H	-2.42438436	-0.38029805	1.07277998
H	2.42438436	0.38029805	1.07277998
H	-1.22424781	-0.18523271	3.20220217
H	1.22424781	0.18523271	3.20220217
H	-0.95572168	-0.75012946	-2.22138992
H	0.95572168	0.75012946	-2.22138992

CASSCF Energy = -384.718183787
NEVPT2 Energy = -386.336237146
NIMAG = 0
CASSCF H = -384.535717925
CASSCF G (HO) = -384.578775810
CASSCF G (GR) = -384.578370026

<u>First-Order Saddle Point for Disrotatory Ring Closure of Ortho-divinylbenzene</u>

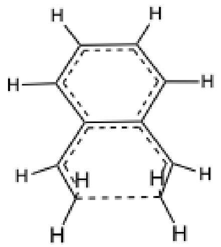

CASSCF(10,10) Optimized Geometry

C	2.44184651	-1.10175370	0.13990814
C	2.46815641	1.04551679	0.10646810
C	1.16125595	1.45177003	-0.23498826
C	1.12530518	-1.48636138	-0.18935246
C	-0.02867919	-0.71652072	0.05090331
C	-0.01112326	0.71823641	0.02861017
C	-1.27809509	1.40555431	-0.01256341
C	-1.31209668	-1.37358404	0.03063160
C	-2.46361895	0.74553174	0.03518261
C	-2.48110444	-0.68357881	0.05738783
H	3.23896323	-1.60189014	-0.38695667
H	3.27688481	1.50932861	-0.43565552
H	2.68309865	0.93925209	1.14572694
H	2.65939120	-0.96833501	1.17552999
H	1.04784061	2.29921859	-0.88882792
H	0.99092612	-2.35076564	-0.81651971
H	-1.26354989	2.47794543	-0.06212458

H	-1.32380939	-2.44704729	0.01442991
H	-3.38830380	1.29008991	0.03525534
H	-3.41883167	-1.20507603	0.07402613

CASSCF Energy = -384.634618097
NEVPT2 Energy = -386.273409333
NIMAG = 1
CASSCF H = -384.453483926
CASSCF G (HO) = -384.494746724
CASSCF G (GR) = -384.494347724

M06-2X Optimized Geometry

C	2.36929683	-1.04066543	0.25907916
C	2.48768668	1.01667440	-0.01205954
C	1.16866673	1.41639185	-0.24234556
C	1.13091447	-1.47226149	-0.20601746
C	-0.03777984	-0.73764293	0.01967446
C	-0.00116292	0.70602139	0.07570592
C	-1.25541723	1.39846874	0.09195043
C	-1.31683028	-1.37153259	-0.06763144
C	-2.44412490	0.74540661	0.09403392
C	-2.47713144	-0.67251174	0.00388632
H	3.26466240	-1.52691688	-0.11065555
H	3.23509373	1.42351739	-0.68922344
H	2.85136333	0.96980043	0.99794730
H	2.40628837	-0.81095833	1.31028424
H	1.01424930	2.25876344	-0.91118104
H	1.06820577	-2.31867644	-0.88162863
H	-1.23133601	2.48031825	0.12317954
H	-1.33494194	-2.45081438	-0.15203038
H	-3.37053715	1.30172782	0.14015687
H	-3.42748639	-1.18803278	-0.02064726

M06-2X Energy = -386.938673614
NIMAG = 1
M06-2X H = -386.762997191
M06-2X G (HO) = -386.804312973
M06-2X G (GR) = -386.803936650

First-Order Saddle Point for Conrotatory Ring Closure of Ortho-divinylbenzene

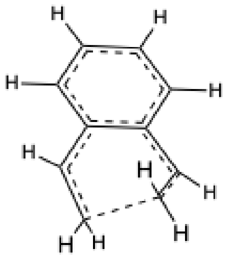

CASSCF(10,10) Optimized Geometry

C	0.69193023	0.05045102	2.48646975
C	-0.69193023	-0.05045092	2.48646975
C	1.38296664	0.09229597	1.27407777
C	-1.38296664	-0.09229605	1.27407777
C	0.71258336	0.03504508	0.05422030
C	-0.71258336	-0.03504520	0.05422028
C	1.45056689	0.06885524	-1.21586382
C	-1.45056689	-0.06885528	-1.21586382
C	0.86487758	-0.45128596	-2.40452218
C	-0.86487758	0.45128602	-2.40452218
H	1.23318779	0.08886535	3.41225958
H	-1.23318779	-0.08886517	3.41225958
H	2.45553994	0.14844516	1.27765644
H	-2.45553994	-0.14844528	1.27765644
H	2.23349595	0.80055976	-1.30288303

H	-2.23349595	-0.80055970	-1.30288303
H	0.49317604	-1.45870352	-2.37243557
H	-0.49317595	1.45870352	-2.37243557
H	1.37899685	-0.23140888	-3.32599473
H	-1.37899697	0.23140910	-3.32599473

CASSCF Energy = -384.625158031
NEVPT2 Energy = -386.255022272
NIMAG = 1
CASSCF H = -384.445885383
CASSCF G (HO) = -384.486238188
CASSCF G (GR) = -384.486144460

UM06-2X Optimized Geometry

C	0.01101011	-0.69185953	2.47795455
C	-0.01101011	0.69185953	2.47795455
C	0.01350114	-1.38240774	1.26962332
C	-0.01350114	1.38240774	1.26962332
C	-0.00256283	-0.71299391	0.05270185
C	0.00256283	0.71299391	0.05270185
C	0.01101011	-1.43335552	-1.21996807
C	-0.01101011	1.43335552	-1.21996807
C	-0.50421379	-0.84908625	-2.38726459
C	0.50421379	0.84908625	-2.38726459
H	0.01954958	-1.23849592	3.41094410
H	-0.01954958	1.23849592	3.41094410
H	0.00502163	-2.46525625	1.26840181
H	-0.00502163	2.46525625	1.26840181
H	0.67940553	-2.28214193	-1.30115613
H	-0.67940553	2.28214193	-1.30115613
H	-1.47267733	-0.36764347	-2.34948517
H	1.47267733	0.36764347	-2.34948517
H	-0.30914335	-1.35350762	-3.32728295
H	0.30914335	1.35350762	-3.32728295

UM06-2X Energy = -386.920554962
NIMAG = 1
$\langle S^2 \rangle$ = 0.8039
UM06-2X H = -386.747427893
UM06-2X G (HO) = -386.788148210
UM06-2X G (GR) = -386.788035895

Second-Order Saddle Point for Internal Rotation of Ortho-divinylbenzene

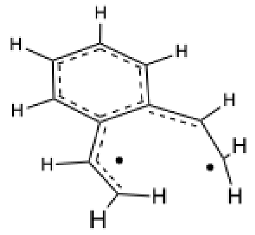

CASSCF(10,10) Optimized Geometry

C	-2.50322383	-1.53792662	0.00000000
C	-2.47578638	1.53911453	0.00000000
C	-1.14040165	1.65542835	0.00000000
C	-1.04245656	-1.67209548	0.00000000
C	-0.00329859	0.70812612	0.00000000
C	0.01574527	-0.74112136	0.00000000
C	1.23666317	1.36942952	0.00000000
C	1.31318636	-1.36427969	0.00000000
C	2.46534927	0.72740560	0.00000000
C	2.49587543	-0.67183936	0.00000000
H	-3.06495206	2.43690699	0.00000000
H	-3.05334561	-1.64072019	-0.91659796
H	-3.05334561	-1.64072019	0.91659796

H -3.01110714 0.61916494 0.00000000
H -0.78906201 2.67136997 0.00000000
H -0.71086382 -2.69711715 0.00000000
H 1.23172791 2.44193905 0.00000000
H 1.34363468 -2.43630177 0.00000000
H 3.37172181 1.30049115 0.00000000
H 3.42945726 -1.20077152 0.00000000

CASSCF Energy = -384.608111174
NEVPT2 Energy = -386.224772631
NIMAG = 2
CASSCF H = -384.431816438
CASSCF G (HO) = -384.474071814
CASSCF G (GR) = -384.473754352

Reaction I

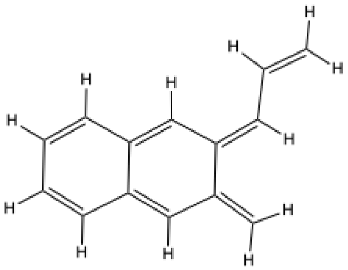

(*E*)-2-Allylidene-3-methylene-2,3-dihydronaphthalene

CASSCF(14,14) Optimized Geometry
C 3.47775165 -0.99995688 0.00000000
C 3.78283523 0.41985663 0.00000000
C 2.79087601 1.33658071 0.00000000
C 1.39668856 0.93360646 0.00000000
C 1.08975397 -0.49178187 0.00000000
C 2.19668541 -1.42936019 0.00000000
C -0.20949664 -0.90748644 0.00000000
C -1.33725347 0.00172933 0.00000000
C -1.02448739 1.45409771 0.00000000
C 0.37734709 1.83516641 0.00000000
C -1.95664110 2.44359203 0.00000000
C -2.62909116 -0.45635633 0.00000000
C -3.07684221 -1.83652513 0.00000000
C -4.37603940 -2.18350009 0.00000000
H 1.97358904 -2.47965829 0.00000000
H 0.60153041 2.88530989 0.00000000
H -3.01118221 2.26464124 0.00000000
H -1.64808311 3.47072454 0.00000000
H -4.67789735 -3.21275548 0.00000000
H -5.15752115 -1.44607745 0.00000000
H -2.34638681 -2.62176887 0.00000000
H -3.42370452 0.26335438 0.00000000
H -0.40070857 -1.96058224 0.00000000
H 3.01630182 2.38630624 0.00000000
H 4.80964647 0.73114152 0.00000000
H 4.28615813 -1.70543496 0.00000000

CASSCF Energy = -537.422612753
NEVPT2 Energy = -539.660951919
NIMAG = 0
CASSCF H = -537.191474074
CASSCF G (HO) = -537.239295415
CASSCF G (GR) = -537.239051852

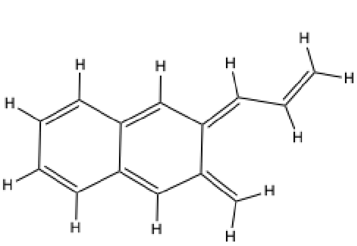

(*Z*)-2-Allylidene-3-methylene-2,3-dihydronaphthalene

CASSCF(14,14) Optimized Geometry

C	3.56475583	-1.26592833	0.00000000
C	3.89026847	0.14980680	0.00000000
C	2.91252604	1.08169264	0.00000000
C	1.51288548	0.69764572	0.00000000
C	1.18666068	-0.72133684	0.00000000
C	2.27772727	-1.67695850	0.00000000
C	-0.11971729	-1.10613424	0.00000000
C	-1.24611125	-0.18734132	0.00000000
C	-0.91047517	1.25981849	0.00000000
C	0.50399884	1.60899597	0.00000000
C	-1.78941366	2.29782873	0.00000000
C	-2.48339090	-0.78749985	0.00000000
C	-3.83691296	-0.26750860	0.00000000
C	-4.91043815	-1.07945758	0.00000000
H	2.03837672	-2.72365087	0.00000000
H	0.74329856	2.65564424	0.00000000
H	-2.84834966	2.19675976	0.00000000
H	-1.41408977	3.30274016	0.00000000
H	-5.90716991	-0.68324864	0.00000000
H	-4.81043968	-2.14921588	0.00000000
H	-4.01582107	0.78457344	0.00000000
H	-2.45445523	-1.86252695	0.00000000
H	-0.34694347	-2.15519542	0.00000000
H	3.15375891	2.12791973	0.00000000
H	4.92167035	0.44567105	0.00000000
H	4.36326924	-1.98260981	0.00000000

CASSCF Energy = -537.416703810
NEVPT2 Energy = -539.656072570
NIMAG = 0
CASSCF H = -537.185303498
CASSCF G (HO) = -537.233410212
CASSCF G (GR) = -537.232830226

First-Order Saddle Point for Disrotatory Ring Closure of (Z)-2-Allylidene-3-methylene-2,3-dihydronaphthalene

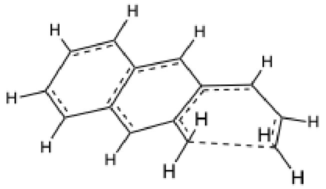

CASSCF(14,14) Optimized Geometry

C	0.05075638	-1.32025654	-0.29319591
C	1.27286928	-0.68045770	-0.14557207
C	1.29688951	0.73377430	0.06695072
C	0.10344568	1.44070158	0.02672503
C	-1.12838348	0.81567127	-0.26035660
C	-1.17731627	-0.62056818	-0.27415774
C	-2.34881409	-1.36583024	0.04651219
C	-3.56426502	-0.90801347	0.54736163
C	-4.22897566	0.30911287	0.34165434
C	-2.26522054	1.59912079	-0.55891507
C	2.56544222	1.37976599	0.27342770
C	3.72073221	0.66609063	0.26890151
C	3.69699776	-0.75395417	0.05567751
C	2.51896892	-1.39963703	-0.14249572

H	-4.95762926	0.60278424	1.07787663
H	-4.41987384	0.68559985	-0.63214442
H	-4.00684899	-1.56253969	1.28134292
H	-2.16240569	-2.41191508	0.21231312
H	-2.85018539	1.32041986	-1.40385214
H	-2.23172261	2.65449122	-0.35624720
H	0.12467142	2.51014366	0.12476619
H	2.49981626	-2.46181669	-0.29886365
H	4.62056745	-1.30032168	0.05573939
H	4.66161530	1.15786363	0.42506915
H	2.58196986	2.44189537	0.43035320
H	0.03031151	-2.39258049	-0.35098744

CASSCF Energy = -537.378602450
NEVPT2 Energy = -539.641701980
NIMAG = 1
CASSCF H = -537.147225624
CASSCF G (HO) = -537.194605093
CASSCF G (GR) = -537.194093768

M06-2X Optimized Geometry

C	0.06181352	-1.33851821	-0.37184973
C	1.25781560	-0.68957141	-0.18128141
C	1.26132676	0.73050786	0.09760804
C	0.08018445	1.42919028	0.05181295
C	-1.13598616	0.79933494	-0.31775970
C	-1.17609060	-0.64804903	-0.32083874
C	-2.32869127	-1.35279540	0.03733127
C	-3.49239398	-0.83015768	0.63215682
C	-4.17437886	0.33684283	0.42183554
C	-2.23553426	1.53219963	-0.70645227
C	2.51983411	1.37227852	0.36052746
C	3.67456616	0.67177766	0.34818708
C	3.67159138	-0.73638078	0.06922720
C	2.51435362	-1.38599537	-0.18374769
H	-4.85140840	0.67842979	1.19693719
H	-4.35510727	0.75593795	-0.54587956
H	-3.83605209	-1.42622606	1.47640696
H	-2.17105816	-2.41608577	0.18742474
H	-2.86102646	1.13593711	-1.48438166
H	-2.25437132	2.60590790	-0.56659992
H	0.08908545	2.50560566	0.17899409
H	2.50915893	-2.44864295	-0.39178449
H	4.60986446	-1.27419104	0.06416049
H	4.61502929	1.16721233	0.54740525
H	2.51809973	2.43549288	0.56569529
H	0.05329776	-2.41753078	-0.47535281

M06-2X Energy = -540.561164578
NIMAG = 1
M06-2X H = -540.336158287
M06-2X G (HO) = -540.383708502
M06-2X G (GR) = -540.383234823

First-Order Saddle Point for Conrotatory Ring Closure of (Z)-2-Allylidene-3-methylene-2,3-dihydronaphthalene

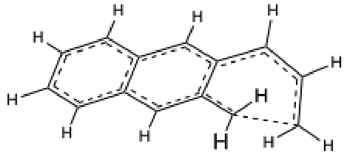

CASSCF(14,14) Optimized Geometry

C	0.05812966	-1.30231708	-0.20663276
C	-1.22651890	-0.68906885	-0.05845683
C	-1.30307732	0.71852706	-0.04504655
C	-0.10681726	1.47184179	-0.21360014
C	1.12837779	0.87505494	-0.39082826
C	1.20562338	-0.57575735	-0.34608558
C	2.51322029	-1.26028770	-0.42498093
C	3.62047609	-0.86797128	0.27382650
C	3.64984930	0.19735134	1.21208093
C	2.29624099	1.67878282	-0.61183736
C	-2.56683675	1.33764718	0.11646918
C	-3.70498057	0.58164319	0.25996829
C	-3.62634977	-0.82724162	0.24673699
C	-2.40912324	-1.44785556	0.08996841
H	4.58820509	0.53400285	1.60739202
H	2.76703629	0.51611967	1.72201918
H	4.54825136	-1.37037359	0.06468778
H	2.58060869	-2.12127447	-1.06326718
H	3.13978458	1.29456119	-1.13892874
H	2.20642187	2.74605232	-0.54455008
H	-0.17771505	2.54334045	-0.24018713
H	-2.34711918	-2.52000786	0.08136147
H	-4.51890375	-1.41204110	0.35987629
H	-4.65759316	1.06054308	0.38175591
H	-2.62604678	2.40991872	0.12365954
H	0.11454869	-2.37524408	-0.18197151

CASSCF Energy = -537.387350020
NEVPT2 Energy = -539.639535886
NIMAG = 1
CASSCF H = -537.157984155
CASSCF G (HO) = -537.205177305
CASSCF G (GR) = -537.204753106

UM06-2X Optimized Geometry

C	0.05001606	-1.31484572	-0.22341128
C	-1.22288408	-0.69850692	-0.07306653
C	-1.28877989	0.71525933	-0.02959003
C	-0.09185665	1.45808513	-0.17739725
C	1.13483790	0.85546157	-0.35781846
C	1.20263435	-0.59382714	-0.33729486
C	2.50270948	-1.27008669	-0.41158834
C	3.60392841	-0.82618818	0.25900225
C	3.59170327	0.25285537	1.16402011
C	2.30946242	1.64678749	-0.57533275
C	-2.54502308	1.33597605	0.13849186
C	-3.68541268	0.58556697	0.25932930
C	-3.61817721	-0.81909189	0.21559710
C	-2.40941981	-1.44618285	0.05201171
H	4.51929161	0.66363663	1.53476592
H	2.68864639	0.53795260	1.67915460
H	4.55396388	-1.30891584	0.05834833
H	2.56953969	-2.16293056	-1.02035252
H	3.13558238	1.26554674	-1.14986801
H	2.25075317	2.71606822	-0.42857357
H	-0.15524130	2.54009549	-0.18968585
H	-2.35137217	-2.52709425	0.01960505
H	-4.52409512	-1.40184538	0.31130995
H	-4.64298750	1.07173595	0.38796616

H -2.59285665 2.41743254 0.16836475
H 0.10047143 -2.39789512 -0.21551080

UM06-2X Energy = -540.561379117
NIMAG = 1
<S^2> = 0.8620
UM06-2X H = -540.338919141
UM06-2X G (HO) = -540.386358779
UM06-2X G (GR) = -540.385915062

Second-Order Saddle Point for Internal Rotation of (Z)-2-Allylidene-3-methylene-2,3-dihydronaphthalene

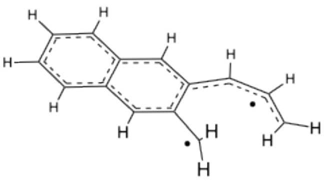

CASSCF(14,14) Optimized Geometry

C 2.11615109 1.98386288 0.00000000
C 0.99593520 1.01860690 0.00000000
C 1.16531527 -0.42071962 0.00000000
C 2.38659525 -1.19994378 0.00000000
C 3.79582691 -0.99458808 0.00000000
C 4.61815262 0.09527795 0.00000000
C -0.00092867 -1.19041610 0.00000000
C -1.30339718 -0.64700377 0.00000000
C -2.46839929 -1.46473753 0.00000000
C -3.71496010 -0.89916146 0.00000000
C -3.85939384 0.51119691 0.00000000
C -2.75060630 1.31737864 0.00000000
C -1.44994855 0.75252742 0.00000000
C -0.27070487 1.54585314 0.00000000
H 2.51841807 2.35663509 -0.92062557
H 2.51841807 2.35663509 0.92062557
H 5.67905092 -0.06422526 0.00000000
H 4.28537893 1.10670185 0.00000000
H 4.31942081 -1.93561924 0.00000000
H 2.16139483 -2.24956536 0.00000000
H -0.38040307 2.61446476 0.00000000
H -2.85707045 2.38595343 0.00000000
H -4.84135246 0.94384873 0.00000000
H -4.58940172 -1.52122891 0.00000000
H -2.35705400 -2.53276944 0.00000000
H 0.09071220 -2.25967717 0.00000000

CASSCF Energy = -537.365745891
NEVPT2 Energy = -539.612021341
NIMAG = 2
CASSCF H = -537.137809488
CASSCF G (HO) = -537.185607776
CASSCF G (GR) = -537.185245534

Second-Order Saddle Point for Internal Rotation of (Z)-2-Allylidene-3-methylene-2,3-dihydronaphthalene

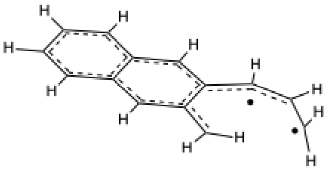

CASSCF(14,14) Optimized Geometry

C -4.65063261 0.13107446 0.00000000
C -3.77438934 -1.05263548 0.00000000
C -2.43930938 -1.23595285 0.00000000
C -2.05228259 1.95871990 0.00000000
C -1.18969301 -0.43092081 0.00000000
C -1.02011585 1.02448292 0.00000000
C -0.05053863 -1.19953177 0.00000000

C	0.30098495	1.53108881	0.00000000
C	1.27788104	-0.67926872	0.00000000
C	1.44920098	0.72174312	0.00000000
C	2.41443226	-1.51794095	0.00000000
C	2.76762797	1.25612880	0.00000000
C	3.68077351	-0.98119366	0.00000000
C	3.85648239	0.42322344	0.00000000
H	-5.11447640	0.44614240	-0.91569203
H	-5.11447640	0.44614240	0.91569203
H	-4.32672161	-1.98119753	0.00000000
H	-3.08111488	1.69671951	0.00000000
H	-2.18643843	-2.28033588	0.00000000
H	-1.81126484	3.00345546	0.00000000
H	-0.14984501	-2.26818306	0.00000000
H	0.42566252	2.59727406	0.00000000
H	2.27997508	-2.58344052	0.00000000
H	2.89929885	2.32176305	0.00000000
H	4.53930839	-1.62465063	0.00000000
H	4.84817944	0.83335418	0.00000000

CASSCF Energy = -537.358896121
NEVPT2 Energy = -539.601906162
NIMAG = 2
CASSCF H = -537.131298116
CASSCF G (HO) = -537.178633084
CASSCF G (GR) = -537.178243754

Reaction J

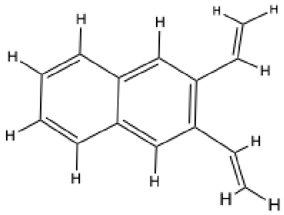

2,3-Divinylnaphthalene

CASSCF(14,14) Optimized Geometry

C	-2.64414239	-0.35017309	2.84389294
C	2.64414239	0.35017309	2.84389294
C	-1.45122242	0.19779898	2.59076656
C	1.45122242	-0.19779898	2.59076656
C	-0.71319604	0.08407348	1.31002343
C	0.71319604	-0.08407348	1.31002343
C	-1.37611806	0.17187931	0.11127780
C	1.37611806	-0.17187931	0.11127780
C	-0.69867796	0.08649664	-1.13366484
C	0.69867796	-0.08649664	-1.13366484
C	-1.38576114	0.17508884	-2.37537729
C	1.38576114	-0.17508884	-2.37537729
C	-0.70323825	0.08912260	-3.55855810
C	0.70323825	-0.08912260	-3.55855810
H	-3.13001013	-0.20125787	3.78921307
H	3.13001013	0.20125787	3.78921307
H	-0.97836375	0.77674866	3.36334313
H	0.97836375	-0.77674866	3.36334313
H	-3.15392137	-0.96447706	2.12561072
H	3.15392137	0.96447706	2.12561072
H	-2.43679261	0.33548084	0.10591352
H	2.43679261	-0.33548084	0.10591352
H	-2.45123363	0.30932468	-2.37270700
H	2.45123363	-0.30932468	-2.37270700
H	-1.22945869	0.15608288	-4.49141800

H	1.22945869	-0.15608288	-4.49141800

CASSCF Energy = -537.464714930
NEVPT2 Energy = -539.712042933
NIMAG = 0
CASSCF H = -537.230985072
CASSCF G (HO) = -537.279968437
CASSCF G (GR) = -537.279287780

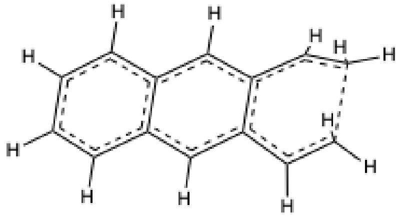

First-Order Saddle Point for Disrotatory Ring Closure of 2,3-Divinylnaphthalene

CASSCF(14,14) Optimized Geometry

C	-3.66989654	1.05209180	0.19260331
C	-2.37842761	1.47565343	-0.19589085
C	-1.19520925	0.72508019	0.01950514
C	-1.19957725	-0.71803388	-0.00736421
C	-2.38700681	-1.45306577	-0.25154786
C	-3.67688691	-1.03728145	0.15098533
C	0.05136785	-1.38683283	-0.05477576
C	1.25982751	-0.72066928	-0.00637804
C	1.26412376	0.71305902	0.01997384
C	0.05965216	1.38773899	-0.00357143
C	2.53189346	1.39930886	0.05085622
C	3.69843778	0.70814748	0.05663354
C	3.69412680	-0.73161340	0.03024305
C	2.52347224	-1.41514501	-0.00078191
H	-4.50961021	-1.48379757	-0.36943183
H	-3.84625812	-0.97790778	1.20400324
H	-2.30361112	-2.26989444	-0.94653548
H	0.06403927	2.46176859	-0.02057778
H	2.51897833	-2.48874367	-0.02117748
H	4.62912344	-1.25818865	0.03435699
H	4.63656995	1.22863621	0.07991448
H	2.53382082	2.47291365	0.06982706
H	0.04937362	-2.45949866	-0.11142316
H	-2.29112288	2.31748731	-0.85981615
H	-4.50109961	1.52355005	-0.30785580
H	-3.83731275	0.95227171	1.24287716

CASSCF Energy = -537.372075351
NEVPT2 Energy = -539.640649975
NIMAG = 1
CASSCF H = -537.140262992
CASSCF G (HO) = -537.187565236
CASSCF G (GR) = -537.186913499

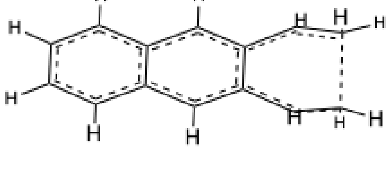

First-Order Saddle Point for Conrotatory Ring Closure of 2,3-Divinylnaphthalene

CASSCF(14,14) Optimized Geometry

C	-0.84590352	0.47145104	-3.62955487
C	0.84590352	-0.47145104	-3.62955487
C	-1.45119226	-0.02946394	-2.44357575
C	1.45119226	0.02946394	-2.44357575

C	-0.72852886	-0.01902720	-1.16964460
C	0.72852886	0.01902720	-1.16964460
C	-1.39002132	-0.06185153	0.02867245
C	1.39002132	0.06185153	0.02867245
C	-0.70345539	-0.03778789	1.28330516
C	0.70345539	0.03778789	1.28330516
C	-1.39224911	-0.07329118	2.51698720
C	1.39224911	0.07329118	2.51698720
C	-0.70383698	-0.03687087	3.70728838
C	0.70383698	0.03687087	3.70728838
H	-1.36904359	0.26740700	-4.54995740
H	1.36904359	-0.26740700	-4.54995740
H	-0.44666409	1.46799755	-3.60187090
H	0.44666409	-1.46799755	-3.60187090
H	-2.27146220	-0.71799910	-2.53271926
H	2.27146220	0.71799910	-2.53271926
H	-2.46435952	-0.08355321	0.03607169
H	2.46435952	0.08355321	0.03607169
H	-2.46486664	-0.12753490	2.51552069
H	2.46486664	0.12753490	2.51552069
H	-1.23640645	-0.06402647	4.63856303
H	1.23640645	0.06402647	4.63856303

CASSCF Energy = -537.374766011
NEVPT2 Energy = -539.637317388
NIMAG = 1
CASSCF H = -537.143752169
CASSCF G (HO) = -537.189821590
CASSCF G (GR) = -537.189449703

Reaction K

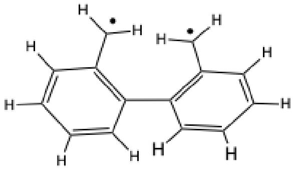

Ortho,ortho'-bibenzyl (singlet state)

CASSCF(14,14) Optimized Geometry

C	-3.55806351	-0.18341400	0.35902628
C	3.55806351	0.18341400	0.35902628
C	-2.94822001	0.57835567	-0.61670193
C	2.94822001	-0.57835567	-0.61670193
C	-2.77923989	-0.87287772	1.29219320
C	2.77923989	0.87287772	1.29219320
C	-1.53246999	0.68708980	-0.70610818
C	1.53246999	-0.68708980	-0.70610818
C	-1.38659728	-0.78723872	1.22402766
C	1.38659728	0.78723872	1.22402766
C	-0.95973939	1.49096310	-1.70701894
C	0.95973939	-1.49096310	-1.70701894
C	-0.74659836	-0.03246148	0.24980737
C	0.74659836	0.03246148	0.24980737
H	-4.62882996	-0.24291527	0.40141818
H	4.62882996	0.24291527	0.40141818
H	-3.54774117	1.11390972	-1.32827148
H	3.54774117	-1.11390972	-1.32827148
H	-3.24339509	-1.46658981	2.05539730
H	3.24339509	1.46658981	2.05539730
H	-1.58662665	2.01514244	-2.40046308
H	1.58662665	-2.01514244	-2.40046308
H	-0.79107511	-1.32266855	1.93892982

H 0.79107511 1.32266855 1.93892982
H -0.09787586 -1.61438048 -1.80084416
H 0.09787586 1.61438048 -1.80084416

CASSCF Energy = -537.399814775
NEVPT2 Energy = -539.650908812
NIMAG = 0
CASSCF H = -537.169827401
CASSCF G (HO) = -537.219303390
CASSCF G (GR) = -537.218275862

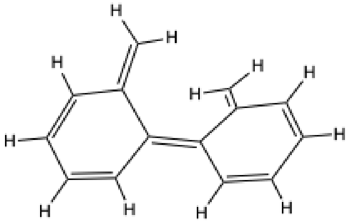

<u>(*Z*)-6,6'-dimethylene-[1,1'-bi(cyclohexylidene)]-2,2',4,4'-tetraene</u>

CASSCF(14,14) Optimized Geometry

C	-0.68354177	0.08999307	0.32790710
C	0.68354177	-0.08999307	0.32790710
C	-1.08568788	1.23186874	-1.85833250
C	1.08568788	-1.23186874	-1.85833250
C	-1.46899211	-0.02008505	1.56177725
C	1.46899211	0.02008505	1.56177725
C	-1.50311720	0.40861347	-0.87720905
C	1.50311720	-0.40861347	-0.87720905
C	-2.79716349	-0.25743476	1.53618957
C	2.79716349	0.25743476	1.53618957
C	-2.89773536	-0.05585722	-0.87434058
C	2.89773536	0.05585722	-0.87434058
C	-3.52013397	-0.36177447	0.27684666
C	3.52013397	0.36177447	0.27684666
H	-0.10747307	1.66712260	-1.86481844
H	0.10747307	-1.66712260	-1.86481844
H	-0.97594398	-0.00136665	2.50948538
H	0.97594398	0.00136665	2.50948538
H	-1.73124826	1.46779382	-2.68330251
H	1.73124826	-1.46779382	-2.68330251
H	-3.33128595	-0.39912909	2.45613612
H	3.33128595	0.39912909	2.45613612
H	-3.42711186	-0.06452410	-1.80839752
H	3.42711186	0.06452410	-1.80839752
H	-4.55256796	-0.65366375	0.28548567
H	4.55256796	0.65366375	0.28548567

CASSCF Energy = -537.393123992
NEVPT2 Energy = -539.630681040
NIMAG = 0
CASSCF H = -537.160946857
CASSCF G (HO) = -537.210859599
CASSCF G (GR) = -537.209696102

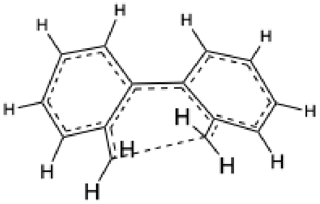

<u>First-Order Saddle Point for Disrotatory Ring Opening of 9,10-Dihydrophenanthrene</u>

CASSCF(14,14) Optimized Geometry

C	3.35597777	-0.61175817	0.65642017
C	2.86181545	0.63756049	0.45700106

C	1.59353602	0.84763741	-0.18961209
C	1.28897429	2.11897802	-0.65934688
C	0.71838576	-0.26944590	-0.38728315
C	1.31912196	-1.56902087	-0.23807491
C	2.58098674	-1.73619342	0.25233084
C	-0.71938390	-0.17177029	-0.44315577
C	-1.47970986	0.93210799	0.09924215
C	-2.71671224	0.60006791	0.76611698
C	-3.33958364	-0.59523386	0.59499824
C	-2.73320937	-1.57613146	-0.24093768
C	-1.47519433	-1.37236571	-0.71571767
C	-1.18200493	2.30531025	0.04266771
H	1.93117452	2.94501996	-0.41575840
H	0.71412253	2.22282100	-1.54920328
H	2.97184825	-2.72705030	0.38242635
H	0.74215138	-2.44615436	-0.43495715
H	-1.02094471	-2.13934755	-1.30786180
H	-3.25477147	-2.48525405	-0.47082970
H	-4.29591751	-0.78130984	1.04392445
H	-3.19501138	1.37574852	1.33306837
H	-1.68418729	2.94592643	0.74616706
H	-0.89627033	2.78404331	-0.85781342
H	3.44691968	1.50196505	0.70802635
H	4.32635117	-0.75477880	1.09133410

CASSCF Energy = -537.3691333456
NEVPT2 Energy = -539.6351116598
NIMAG = 1
CASSCF H = -537.137428429
CASSCF G (HO) = -537.189412004
CASSCF G (GR) = -537.186909777

M06-2X Optimized Geometry

C	3.25445634	-0.59532535	0.75667457
C	2.77282399	0.65279357	0.58529331
C	1.56885033	0.87753705	-0.17958306
C	1.34238780	2.10144412	-0.72879854
C	0.69577223	-0.24962500	-0.43455674
C	1.31881410	-1.54535135	-0.35021089
C	2.53796908	-1.70965602	0.21633800
C	-0.70649263	-0.14828097	-0.49151260
C	-1.46520058	0.97308066	0.06644822
C	-2.63036598	0.59974241	0.85382870
C	-3.23696738	-0.59476413	0.71217825
C	-2.69458873	-1.55151440	-0.20448336
C	-1.48311311	-1.33837970	-0.76100967
C	-1.25913792	2.31540997	-0.06888289
H	1.96930043	2.94630292	-0.47226433
H	0.73996401	2.18759688	-1.61479673
H	2.95251438	-2.70377877	0.31414085
H	0.76593978	-2.42349974	-0.64621355
H	-1.06163715	-2.09067360	-1.41196940
H	-3.24167708	-2.45832557	-0.42312618
H	-4.15131150	-0.81343620	1.24596231
H	-3.07088672	1.37045847	1.47347535
H	-1.79712334	2.98228162	0.59528828
H	-0.80428637	2.77721825	-0.91788259
H	3.32686684	1.51972046	0.92282547
H	4.19126337	-0.75903013	1.27124646

M06-2X Energy = -540.553843141
NIMAG = 1
M06-2X H = -540.328613749
M06-2X G (HO) = -540.376507202
M06-2X G (GR) = -540.375728967

First-Order Saddle Point for Conrotatory Ring Opening of 9,10-Dihydrophenanthrene

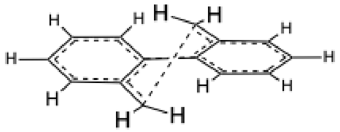

CASSCF(14,14) Optimized Geometry

C	-1.43754244	-0.58917093	1.38758469
C	1.43754244	0.58917093	1.38758469
C	-2.83429074	-0.63934189	1.40500700
C	2.83429074	0.63934189	1.40500700
C	-3.55300403	-0.11144480	0.33860576
C	3.55300403	0.11144480	0.33860576
C	-0.74583894	-0.00696159	0.33412993
C	0.74583894	0.00696159	0.33412993
C	-2.87959695	0.46924475	-0.72765738
C	2.87959695	-0.46924475	-0.72765738
C	-1.47486389	0.55257213	-0.75333416
C	1.47486389	-0.55257213	-0.75333416
C	-0.81289333	1.14748096	-1.87469304
C	0.81289333	-1.14748096	-1.87469304
H	-0.88474780	-1.02335596	2.19874120
H	0.88474780	1.02335596	2.19874120
H	-3.34341621	-1.09311318	2.23315263
H	3.34341621	1.09311318	2.23315263
H	-4.62553024	-0.14994635	0.33676341
H	4.62553024	0.14994635	0.33676341
H	-3.43749261	0.88074595	-1.54759872
H	3.43749261	-0.88074595	-1.54759872
H	0.18950772	1.50684536	-1.80904043
H	-0.18950772	-1.50684536	-1.80904043
H	-1.39566624	1.46918249	-2.71578264
H	1.39566624	-1.46918249	-2.71578264

CASSCF Energy = -537.395733197
NEVPT2 Energy = -539.652216138
NIMAG = 1
CASSCF H = -537.165965479
CASSCF G (HO) = -537.212126272
CASSCF G (GR) = -537.211809607

UM06-2X Optimized Geometry

C	-1.44130932	-1.41661305	-0.53485825
C	1.44130966	-1.41661292	0.53485857
C	-2.83211707	-1.42271735	-0.57810074
C	2.83211745	-1.42271705	0.57810054
C	-3.53695906	-0.32855192	-0.09687247
C	3.53695919	-0.32855152	0.09687211
C	-0.74150411	-0.34591618	-0.00255843
C	0.74150420	-0.34591608	0.00255905
C	-2.85603140	0.75133589	0.43644890
C	2.85603130	0.75133622	-0.43644909
C	-1.45478024	0.76586192	0.51725243
C	1.45478008	0.76586208	-0.51725216
C	-0.77554927	1.88515807	1.08804308

C	0.77554872	1.88515750	-1.08804335
H	-0.88524079	-2.25195097	-0.94175226
H	0.88524130	-2.25195107	0.94175229
H	-3.35580364	-2.27005990	-0.99838261
H	3.35580433	-2.27005973	0.99838173
H	-4.61772662	-0.31745259	-0.13621740
H	4.61772675	-0.31745216	0.13621665
H	-3.40596873	1.59981104	0.82399396
H	3.40596849	1.59981152	-0.82399400
H	0.22065482	1.79427313	1.48633527
H	-0.22065636	1.79427246	-1.48633291
H	-1.33866442	2.77230284	1.33984502
H	1.33866349	2.77230174	-1.33984808

UM06-2X Energy = -540.574545675
NIMAG = 1
$\langle S^2 \rangle$ = 0.8674
UM06-2X H = -540.351549816
UM06-2X G (HO) = -540.398078400
UM06-2X G (GR) = -540.397717985

Data for main text Scheme 7.

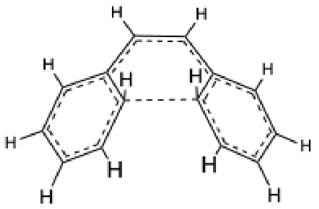

First-Order Saddle Point for Disrotatory Ring Closure of (Z)-Stilbene

CASSCF(14,14) Optimized Geometry

C	-0.82334685	2.02198566	0.27166065
C	0.57627853	2.11101949	0.19115725
C	-1.52466590	0.81872571	0.31030803
C	-0.95537824	-0.38057723	0.91542478
C	-1.52111858	-1.66192759	0.47997717
C	-2.58986159	-1.72420339	-0.34374584
C	-3.24479035	-0.51780474	-0.78819327
C	-2.73850139	0.69623993	-0.45324637
C	1.42135627	1.00500818	0.18056241
C	1.05659574	-0.21726563	0.88239727
C	1.77975070	-1.43394682	0.50214971
C	2.78111546	-1.41040028	-0.40417221
C	3.21123276	-0.16422775	-0.99080872
C	2.56743913	0.99202090	-0.68800909
H	-1.36832307	2.89917494	-0.02825984
H	0.96942236	3.04240660	-0.17523869
H	-0.89057619	-0.31194001	1.98142995
H	-1.08961645	-2.56516844	0.86769797
H	-2.99230352	-2.67546390	-0.63657435
H	-4.11941713	-0.58949314	-1.40526363
H	-3.19756561	1.59406926	-0.82309960
H	1.01880554	-0.07901975	1.94270150
H	1.51837086	-2.35397542	0.98985930
H	3.29988388	-2.31462135	-0.66064431
H	4.03279823	-0.16330896	-1.68063282
H	2.86488994	1.91412813	-1.15141765

CASSCF Energy = -537.368779111
NEVPT2 Energy = -539.633519533
NIMAG = 1
CASSCF H = -537.135371831
CASSCF G (HO) = -537.182532585
CASSCF G (GR) = -537.181441545

M06-2X Optimized Geometry

C	-0.82736274	2.02656523	0.36495402
C	0.54395287	2.12412504	0.11124403
C	-1.49927484	0.80512763	0.40507696
C	-0.86223766	-0.44135736	0.83208511
C	-1.33442600	-1.65198245	0.16688327
C	-2.43767108	-1.64878160	-0.60899375
C	-3.20469717	-0.45286438	-0.78313022
C	-2.75060209	0.72194487	-0.27983517
C	1.36571384	1.00815154	0.10212151
C	0.99461411	-0.11869473	0.94103507
C	1.75349234	-1.34889123	0.76471439
C	2.71933410	-1.44678881	-0.17087206
C	3.09116646	-0.31210401	-0.96550987
C	2.45333715	0.87512759	-0.80830485
H	-1.42725764	2.90672760	0.16235202
H	0.90235457	3.04604336	-0.33186568
H	-0.85819292	-0.57637996	1.90649460
H	-0.80664213	-2.57760216	0.35824721
H	-2.77766530	-2.56672612	-1.07007804
H	-4.11753167	-0.48166072	-1.36143996
H	-3.27501708	1.64678837	-0.48812202
H	0.90571635	0.19137166	1.97369089
H	1.52358397	-2.19220828	1.40463991
H	3.25558563	-2.37750877	-0.30075279
H	3.88561620	-0.41278471	-1.69188205
H	2.72587602	1.73201669	-1.41224669

M06-2X Energy = -540.561877693
NIMAG = 1
M06-2X H = -540.335723467
M06-2X G (HO) = -540.382387833
M06-2X G (GR) = -540.381661580

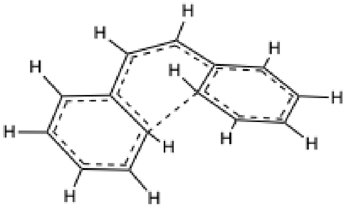

First-Order Saddle Point for Conrotatory Ring Closure of (Z)-Stilbene

CASSCF(14,14) Optimized Geometry

C	-0.67545241	0.06471224	2.14045499
C	0.67545241	-0.06471224	2.14045499
C	-1.44619370	0.17315678	0.90885059
C	1.44619370	-0.17315678	0.90885059
C	-0.71890283	0.57687342	-0.31537666
C	0.71890283	-0.57687342	-0.31537666
C	-1.50566530	0.54373109	-1.54713084
C	1.50566530	-0.54373109	-1.54713084
C	-2.79414916	0.08242533	-1.57524087
C	2.79414916	-0.08242533	-1.57524087

C	-3.44714332	-0.31341347	-0.38414805
C	3.44714332	0.31341347	-0.38414805
C	-2.75726247	-0.25580484	0.83652649
C	2.75726247	0.25580484	0.83652649
H	-1.20832682	0.06974687	3.07343028
H	1.20832682	-0.06974687	3.07343028
H	-0.16533928	1.49461627	-0.19942827
H	0.16533928	-1.49461627	-0.19942827
H	-1.04252815	0.88687789	-2.45273521
H	1.04252815	-0.88687789	-2.45273521
H	-3.33138418	0.05566803	-2.50457217
H	3.33138418	-0.05566803	-2.50457217
H	-4.46274185	-0.65521204	-0.41339522
H	4.46274185	0.65521204	-0.41339522
H	-3.25191092	-0.57555312	1.73542998
H	3.25191092	0.57555312	1.73542998

CASSCF Energy = -537.363308874
NEVPT2 Energy = -539.622002113
NIMAG = 1
CASSCF H = -537.133293576
CASSCF G (HO) = -537.179102874
CASSCF G (GR) = -537.178713023

UM06-2X Optimized Geometry

C	0.67231226	0.06589010	-2.14206958
C	-0.67231226	-0.06589010	-2.14206958
C	1.43049002	0.18069144	-0.91055143
C	-1.43049002	-0.18069144	-0.91055143
C	0.69386905	0.57374293	0.30386984
C	-0.69386905	-0.57374293	0.30386984
C	1.45629251	0.53841954	1.53970742
C	-1.45629251	-0.53841954	1.53970742
C	2.74892759	0.10071111	1.57639205
C	-2.74892759	-0.10071111	1.57639205
C	3.42044830	-0.26878256	0.39508134
C	-3.42044830	0.26878256	0.39508134
C	2.74732542	-0.21435039	-0.82751840
C	-2.74732542	0.21435039	-0.82751840
H	1.21735394	0.07408555	-3.07835293
H	-1.21735394	-0.07408555	-3.07835293
H	0.10042831	1.48085678	0.18469726
H	-0.10042831	-1.48085678	0.18469726
H	0.96883821	0.86938065	2.44784641
H	-0.96883821	-0.86938065	2.44784641
H	3.27802110	0.07100245	2.51986361
H	-3.27802110	-0.07100245	2.51986361
H	4.45037651	-0.59234232	0.43392485
H	-4.45037651	0.59234232	0.43392485
H	3.25650668	-0.51990104	-1.73400712
H	-3.25650668	0.51990104	-1.73400712

UM06-2X Energy = -540.549415988
NIMAG = 1
<S^2> = 0.9932
UM06-2X H = -540.326555463
UM06-2X G (HO) = -540.372718622
UM06-2X G (GR) = -540.372260763

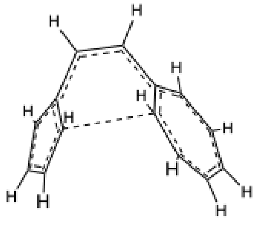

First-Order Saddle Point for Disrotatory Ring Closure of Compound **25**

CASSCF(14,14) Optimized Geometry

C	1.58568290	1.87047659	-0.39771564
C	2.02638653	0.50871341	-0.02373605
C	1.86502853	-0.67305787	-0.87111975
C	2.20866921	-1.82271757	-0.08609469
C	2.55898121	-1.38799705	1.15959726
C	2.43507483	0.07794469	1.19648515
C	0.28715002	2.16240786	-0.54965837
C	-0.77223161	1.12943624	-0.34587703
C	-0.69814643	-0.00997026	-1.22546531
C	-1.50444386	-1.19116749	-1.19118155
C	-2.49644849	-1.54793157	-0.33232586
C	-3.05363843	-0.77729096	0.76667361
C	-2.69687578	0.45421196	1.18628894
C	-1.64164838	1.32242451	0.68587826
H	1.83665147	-0.65311386	-1.93893625
H	2.58810356	0.68521486	2.06535994
H	2.87612632	-1.99696161	1.98142277
H	2.22428325	-2.83285024	-0.43834974
H	2.32185939	2.64756463	-0.49779914
H	-1.52595779	2.23964499	1.23408567
H	-3.24667499	0.85607247	2.01764022
H	-3.86056611	-1.25224604	1.29397784
H	-2.95358750	-2.50548412	-0.49860774
H	-1.27852461	-1.90183260	-1.96621312
H	-0.19489929	0.16893105	-2.15114774
H	-0.01964957	3.17002962	-0.76505221

CASSCF Energy = -537.321258124
NEVPT2 Energy = -539.563060476
NIMAG = 1
CASSCF H = -537.091435874
CASSCF G (HO) = -537.140236176
CASSCF G (GR) = -537.139151123

M06-2X Optimized Geometry

C	1.37750008	1.88801888	-0.23987571
C	2.01046152	0.62524447	-0.10867615
C	1.69925412	-0.63607269	-0.79073517
C	2.34244459	-1.65862455	-0.07084341
C	3.01684333	-1.08179829	1.00254386
C	2.85193793	0.31535337	0.96077120
C	0.04240224	2.12809138	-0.41538920
C	-0.92841470	1.07903698	-0.37769138
C	-0.59540623	-0.17931092	-1.02845103
C	-1.18693412	-1.43070104	-0.79464425
C	-2.22724424	-1.77939310	0.03075454
C	-3.10459100	-0.92810107	0.72155635
C	-3.09059031	0.44373028	0.76932200
C	-2.11783464	1.32844457	0.27651055
H	1.58187113	-0.70698463	-1.85892698
H	3.18472121	1.01742563	1.71055581
H	3.58530214	-1.61708820	1.74911726
H	2.39660079	-2.69411613	-0.36935813
H	1.97512484	2.74821712	0.04815732

H	-2.28726153	2.37381835	0.51596765
H	-3.90508779	0.91111709	1.30863093
H	-3.91976884	-1.41550263	1.24268767
H	-2.46218407	-2.83540596	0.07643778
H	-0.74292707	-2.24373671	-1.35856330
H	-0.16100781	-0.03674526	-2.00227462
H	-0.31495657	3.14259608	-0.29029968

M06-2X Energy = -540.500152082
NIMAG = 1
M06-2X H = -540.275327815
M06-2X G (HO) = -540.324135772
M06-2X G (GR) = -540.322904889

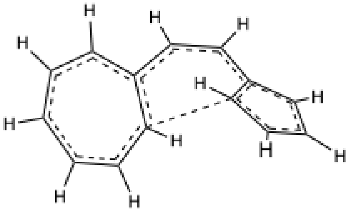

First-Order Saddle Point for Conrotatory Ring Closure of Compound **25**

CASSCF(14,14) Optimized Geometry

C	-2.66276644	-1.63946638	-0.47811676
C	-3.51124976	-0.71510706	0.25761175
C	-3.25096653	0.56711897	0.58968412
C	-1.41770993	-1.41525111	-0.98108150
C	-2.06196783	1.36103394	0.33041112
C	-0.59178052	-0.25329411	-0.90554483
C	-0.89917831	1.01122847	-0.30652769
C	0.16564684	2.04559877	-0.38279371
C	1.48132922	1.83335128	-0.20819062
C	2.10815789	0.56032791	0.14952190
C	3.34133698	0.12466974	-0.22294716
C	1.48092927	-0.52364233	0.91927759
C	3.52330973	-1.24269495	0.27453880
C	2.38768770	-1.61286566	0.95738606
H	-3.08592413	-2.61034510	-0.65612067
H	-4.46480177	-1.10873053	0.55815743
H	-4.02083538	1.08595207	1.13135444
H	-0.97462235	-2.23561237	-1.51668765
H	-2.10543321	2.36317256	0.71538369
H	0.28002747	-0.27567241	-1.52375902
H	-0.16291895	3.04896858	-0.58321591
H	4.40345098	-1.83584330	0.13332285
H	2.22501709	-2.54025957	1.46525215
H	2.14651873	2.66670611	-0.34587626
H	4.05392029	0.66514656	-0.81242259
H	0.60090508	-0.42616171	1.51523255

CASSCF Energy = -537.331422177
NEVPT2 Energy = -539.575831840
NIMAG = 1
CASSCF H = -537.102203067
CASSCF G (HO) = -537.150660852
CASSCF G (GR) = -537.149887798

UM06-2X Optimized Geometry

C	-2.33161817	-1.70062082	-0.35822974
C	-3.28087178	-0.85823661	0.22598384
C	-3.15975581	0.50227065	0.42649313
C	-1.06395655	-1.40314488	-0.80562386

C	-2.07437407	1.34938335	0.21022915
C	-0.31746375	-0.21561188	-0.68202275
C	-0.77603458	1.07063850	-0.21663348
C	0.18874527	2.13991617	-0.24928842
C	1.52806346	1.91285760	-0.12265480
C	2.04153731	0.64662772	0.26235391
C	3.13102978	-0.00891390	-0.33946211
C	1.27165140	-0.35153723	0.96170099
C	3.03681595	-1.37633330	-0.04901149
C	1.92164391	-1.58027618	0.77719955
H	-2.65467805	-2.72213158	-0.52614938
H	-4.22733037	-1.30771696	0.49542608
H	-4.03850318	0.99208716	0.83110936
H	-0.53913371	-2.20395264	-1.31111847
H	-2.24500684	2.38674080	0.47453939
H	0.56864221	-0.18107747	-1.30174022
H	-0.18713989	3.14855844	-0.36601364
H	3.71945654	-2.13963425	-0.38914107
H	1.64029174	-2.51622057	1.23553413
H	2.22063919	2.72048875	-0.33839914
H	3.84975247	0.45555785	-0.99838568
H	0.51881454	-0.14509524	1.70575476

UM06-2X Energy	= -540.519381764
NIMAG	= 1
<S^2>	= 0.0000
UM06-2X H	= -540.295007903
UM06-2X G (HO)	= -540.342744448
UM06-2X G (GR)	= -540.342157169

First-Order Saddle Point for Disrotatory Ring Closure of Compound **26**

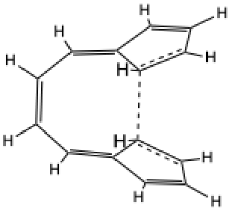

CASSCF(14,14) Optimized Geometry

C	2.60736199	0.40735534	-0.67028236
C	2.60736199	0.40735534	0.67028236
C	1.67823763	-0.39365682	-1.49569213
C	1.67823763	-0.39365682	1.49569213
C	0.34855409	-0.21120898	-1.58141375
C	0.34855409	-0.21120898	1.58141375
C	-0.45647217	0.92594484	-1.01457787
C	-0.45647217	0.92594484	1.01457787
C	-0.58367406	-1.05576768	-2.32146859
C	-0.58367406	-1.05576768	2.32146859
C	-1.75961940	0.76624343	-1.60774767
C	-1.75961940	0.76624343	1.60774767
C	-1.82409446	-0.45884731	-2.31055498
C	-1.82409446	-0.45884731	2.31055498
H	3.37933727	0.95201281	-1.18684661
H	3.37933727	0.95201281	1.18684661
H	2.11691638	-1.21047189	-2.04412985
H	2.11691638	-1.21047189	2.04412985
H	-0.00499402	1.89793823	-1.05942273
H	-0.00499402	1.89793823	1.05942273
H	-0.33002917	-1.99561885	-2.76645756
H	-0.33002917	-1.99561885	2.76645756
H	-2.57897095	1.44219587	-1.47460735

H	-2.57897095	1.44219587	1.47460735
H	-2.70482378	-0.84866741	-2.78067112
H	-2.70482378	-0.84866741	2.78067112

CASSCF Energy = -537.294613652
NEVPT2 Energy = -539.544518710
NIMAG = 1
CASSCF H = -537.066299276
CASSCF G (HO) = -537.114846093
CASSCF G (GR) = -537.113859589

First-Order Saddle Point for Conrotatory Ring Closure of Compound **26**

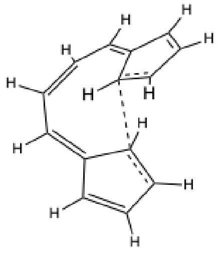

CASSCF(14,14) Optimized Geometry

C	-2.67189789	0.83524096	-1.76668062
C	2.67189789	-0.83524096	-1.76668062
C	-1.43289530	1.43309951	-1.70315292
C	1.43289530	-1.43309951	-1.70315292
C	-2.90770960	0.12743510	-0.50812300
C	2.90770960	-0.12743510	-0.50812300
C	-0.86727172	1.13387096	-0.44166555
C	0.86727172	-1.13387096	-0.44166555
C	-1.82629299	0.29895100	0.30340863
C	1.82629299	-0.29895100	0.30340863
C	-1.69100440	-0.30296740	1.62082467
C	1.69100440	0.30296740	1.62082467
C	-0.66630614	-0.31936646	2.49593291
C	0.66630614	0.31936646	2.49593291
H	-3.35753870	0.87516922	-2.58794417
H	3.35753870	-0.87516922	-2.58794417
H	-0.97676986	2.03597116	-2.45968285
H	0.97676986	-2.03597116	-2.45968285
H	-3.78409219	-0.44341981	-0.27743205
H	3.78409219	0.44341981	-0.27743205
H	-0.01269188	-1.57745612	-0.03771006
H	0.01269188	1.57745612	-0.03771006
H	-2.57021141	-0.83880436	1.93332884
H	2.57021141	0.83880436	1.93332884
H	-0.87171328	-0.82788116	3.42296157
H	0.87171328	0.82788116	3.42296157

CASSCF Energy = -537.313997063
NEVPT2 Energy = -539.559202400
NIMAG = 1
CASSCF H = -537.086080511
CASSCF G (HO) = -537.135088749
CASSCF G (GR) = -537.134457028

Data for main text Figure 7

All data for Figure 7 are listed under Reaction **G** above.

Data for main text Figure 8

7-Methoxy-7-vinylbicyclo[4.2.0]octa-1,3,5-triene

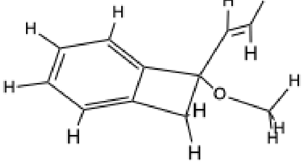

M06-2X Optimized Geometry

C	-0.96312589	-0.09265179	0.01340439
C	0.52021958	-0.29800201	0.27040121
C	0.89812327	0.55032041	-0.75228202
C	2.22458691	0.82641696	-1.01899960
C	3.15562304	0.18553433	-0.20150808
C	2.76828914	-0.67979542	0.82155278
C	1.42507455	-0.94428610	1.08685803
C	-0.52517561	0.83001456	-1.19282531
C	-2.90285925	-1.13990916	-0.83923610
C	-1.69325434	0.54344676	1.16224127
C	-2.23769816	1.74841295	1.19592373
O	-1.58287722	-1.30063520	-0.36903695
H	1.13180072	-1.61670091	1.88155263
H	3.53369174	-1.15406080	1.42125718
H	4.21127452	0.35848661	-0.36460574
H	2.54453632	1.48900670	-1.81198922
H	-0.78925167	0.38794294	-2.15300374
H	-0.84531771	1.86925266	-1.16707254
H	-3.22300581	-2.10679490	-1.21945772
H	-3.58093104	-0.81985054	-0.04433871
H	-2.94904379	-0.40468312	-1.64978501
H	-2.20665898	2.41952438	0.34799536
H	-2.73565511	2.10933628	2.08565760
H	-1.75891896	-0.10094707	2.03513839

M06-2X Energy = -501.501434184
NIMAG = 0
M06-2X H = -501.288634902
M06-2X G (HO) = -501.337605230
M06-2X G (GR) = -501.336510252

First-Order Saddle Point for Outward Conrotatory Ring Opening of 7-Methoxy-7-vinylbicyclo[4.2.0]octa-1,3,5-triene

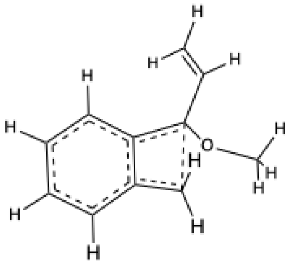

M06-2X Optimized Geometry

C	-2.63776708	-1.54494031	-0.67235093
C	-1.09497125	0.17898380	-0.20085293
C	-2.00603155	1.25518398	0.14131069
C	-1.67233002	2.22156052	1.00069776
C	0.33993429	0.31324752	-0.09689665
C	1.20945454	1.27411719	-0.62228553
C	2.55079816	0.96000191	-0.64959549
C	3.01380166	-0.28118566	-0.15909292
C	2.17328384	-1.18251653	0.44904714
C	0.82022773	-0.81302795	0.59465469
C	-0.21945106	-1.32102612	1.40981198
O	-1.53812638	-0.76191971	-1.07794703
H	-2.98626029	1.25092914	-0.32275920
H	-0.70857080	2.21520638	1.49526083
H	-2.34811559	3.03424194	1.22598237
H	-0.77260868	-0.62192311	2.01695936
H	-0.24008096	-2.35456856	1.74209580
H	2.54964451	-2.10928807	0.86254732
H	4.06789491	-0.51109170	-0.24562097
H	3.25640220	1.64339791	-1.10148975
H	0.83422968	2.18394641	-1.07295872

H	-2.94680744	-2.13109870	-1.53370007
H	-2.31309362	-2.19965981	0.14438437
H	-3.47406268	-0.93385454	-0.32498523

M06-2X Energy = -501.436271872
NIMAG = 1
M06-2X H = -501.226589931
M06-2X G (HO) = -501.274368389
M06-2X G (GR) = -501.274004674

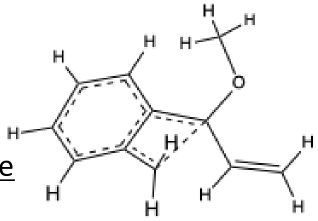

First-Order Saddle Point for Inward Conrotatory Ring Opening of 7-Methoxy-7-vinylbicyclo[4.2.0]octa-1,3,5-triene

M06-2X Optimized Geometry

C	1.58301069	2.10063877	0.90869698
C	1.08745380	-0.01400471	-0.04454371
C	-0.33062682	0.25117710	-0.05339771
C	-0.93772845	-0.81719756	0.62717269
C	-2.29798861	-1.09972687	0.38249099
C	-3.02681187	-0.16931691	-0.31663303
C	-2.44102562	1.01922772	-0.80958574
C	-1.09021390	1.24649921	-0.68610284
C	0.03536276	-1.40661893	1.46641529
C	1.64068932	-1.16209130	-0.75953705
C	2.92306167	-1.28217000	-1.09779764
O	1.99254648	0.94228882	0.19567856
H	-0.63078344	2.11344795	-1.14300327
H	-3.06029086	1.73172871	-1.33663795
H	-4.08464332	-0.33122683	-0.47710953
H	-2.76761412	-1.98259111	0.79635607
H	0.02123843	-2.45774146	1.73468586
H	0.58621088	-0.76404985	2.13590684
H	0.90999328	-1.90205515	-1.05434529
H	3.65586596	-0.54422164	-0.80572770
H	3.25904757	-2.13266437	-1.67244295
H	2.47545443	2.50259984	1.37991587
H	0.83899905	1.84623641	1.66398185
H	1.16518101	2.84448006	0.23105250

M06-2X Energy = -501.452880175
NIMAG = 1
M06-2X H = -501.242588917
M06-2X G (HO) = -501.290211608
M06-2X G (GR) = -501.289850516

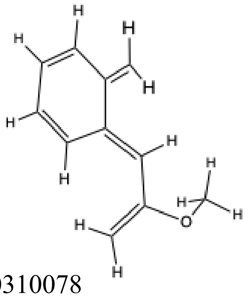

(E)-5-(2-methoxyallylidene)-6-methylenecyclohexa-1,3-diene

M06-2X Optimized Geometry

C	1.92888805	-1.78489158	0.30310078
C	3.02484948	-0.87213305	0.56952433
C	2.87527021	0.44438265	0.37576339
C	1.63253270	0.99573815	-0.15457134
C	0.45513001	0.09082210	-0.21033996

C	0.72260998	-1.33649302	-0.07711866
C	-0.79480324	0.59530289	-0.28792858
C	1.61366141	2.24131588	-0.65130130
C	-2.04409724	-0.14024183	-0.50839250
C	-2.22404145	-1.08317345	-1.43021845
C	-2.91522589	0.40855423	1.61060938
O	-3.12626161	0.29338779	0.21391463
H	2.08583265	-2.84421908	0.45604313
H	3.96648653	-1.27130229	0.92133942
H	3.69368466	1.13183299	0.54740025
H	-0.09997514	-2.02346396	-0.20541879
H	-0.92616996	1.66571130	-0.16360355
H	2.48701375	2.87516444	-0.57425024
H	0.75363452	2.64228933	-1.16844210
H	-3.19248831	-1.54678709	-1.54905181
H	-1.41953261	-1.35599349	-2.09559893
H	-2.13442644	1.13496355	1.84443383
H	-2.64055499	-0.55796985	2.04078291
H	-3.85646617	0.74222998	2.03892050

M06-2X Energy = -501.484336602
NIMAG = 0
M06-2X H = -501.272074636
M06-2X G (HO) = -501.322927879
M06-2X G (GR) = -501.321349808

(Z)-5-(2-methoxyallylidene)-6-methylenecyclohexa-1,3-diene Conformation A

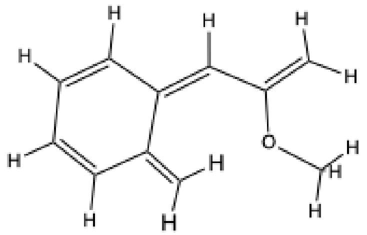

M06-2X Optimized Geometry

C	3.02925428	0.90645891	-0.30343498
C	3.18590811	-0.53404851	-0.38833348
C	2.16810453	-1.34422556	-0.07018986
C	0.88792394	-0.80969010	0.38858818
C	0.64179191	0.63244836	0.15195525
C	1.83472612	1.45263214	-0.03197164
C	-0.56080432	1.24482259	0.09263061
C	0.06419956	-1.57654254	1.11330300
C	-1.90864632	0.67637557	0.00366171
C	-2.97407621	1.34568267	0.46292219
C	-3.21219042	-1.16751639	-0.67140859
O	-1.96103252	-0.52395696	-0.61710741
H	3.88572253	1.53935896	-0.49261059
H	4.14237963	-0.94231124	-0.68499539
H	2.28613633	-2.42035260	-0.08068226
H	1.70889292	2.52808291	-0.02064821
H	-0.55010122	2.32841876	0.06668129
H	0.29765267	-2.61884201	1.28900509
H	-0.83485083	-1.18185714	1.56444632
H	-3.98150666	0.96693395	0.40541012
H	-2.82434260	2.31150760	0.91944746
H	-3.92899066	-0.58463310	-1.25525119
H	-3.61467287	-1.32427682	0.33323346
H	-3.04353783	-2.12622115	-1.15309595

M06-2X Energy = -501.486819918

NIMAG	= 0
M06-2X H	= -501.274309179
M06-2X G (HO)	= -501.323744268
M06-2X G (GR)	= -501.322410257

<u>(Z)-5-(2-methoxyallylidene)-6-methylenecyclohexa-1,3-diene Conformation B</u>

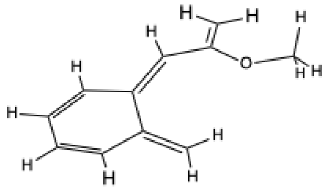

M06-2X Optimized Geometry

C	3.09495001	-0.93412091	-0.15094532
C	3.31532303	0.45432930	0.21893280
C	2.29257042	1.31724347	0.25932252
C	0.93372747	0.90612539	-0.08843267
C	0.67976663	-0.55469166	-0.16327461
C	1.85632735	-1.40401630	-0.34639038
C	-0.52130632	-1.13813649	-0.01467388
C	0.01852738	1.82842019	-0.41326658
C	-1.79248693	-0.48041757	0.36444808
C	-2.10784736	-0.18807759	1.62348702
C	-3.84129881	0.32456301	-0.48072990
O	-2.58172591	-0.26440640	-0.72228496
H	3.94777880	-1.59310118	-0.24225638
H	4.32176930	0.78580657	0.43511169
H	2.45451475	2.36342189	0.48629780
H	1.68744062	-2.44814840	-0.57778886
H	-0.58012935	-2.21370947	-0.15019494
H	0.25792709	2.88123254	-0.33469116
H	-0.95682694	1.56333790	-0.79378560
H	-3.03828046	0.27776741	1.90756269
H	-1.39456387	-0.41127298	2.40092250
H	-3.72630573	1.31135858	-0.02427337
H	-4.44557967	-0.30451154	0.17706865
H	-4.32691463	0.42171585	-1.44718443

M06-2X Energy	= -501.484560456
NIMAG	= 0
M06-2X H	= -501.272258115
M06-2X G (HO)	= -501.321677474
M06-2X G (GR)	= -501.320479862

<u>First-Order Saddle Point for (*E*)–(*Z*) Interconversion of 5-(2-methoxyallylidene)-6-methylenecyclohexa-1,3-diene</u>

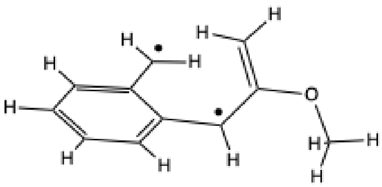

UM06-2X Optimized Geometry

C	3.65737469	-0.26949553	-0.99716163
C	1.82040909	-0.08355784	0.50138407
C	0.92011180	-0.20970546	-0.53122633
C	-0.54325258	-0.23791493	-0.30765127
C	-1.29563575	0.97095224	-0.23547509
C	-2.69846671	0.87084930	-0.07211054
C	-3.32586327	-0.35001112	0.03201269
C	-2.57732312	-1.52349270	-0.02042779
C	-1.19821195	-1.45299679	-0.18956600

C	-0.67776484	2.23144379	-0.29688480
C	1.43536631	0.07955491	1.83002461
O	3.17351595	-0.08569871	0.31164571
H	-0.61357648	-2.36307965	-0.23293380
H	-3.06248734	-2.48572965	0.06698396
H	-4.39915492	-0.39642360	0.15807698
H	-3.27653608	1.78527235	-0.02588081
H	-1.27373765	3.13067726	-0.24721120
H	0.39225549	2.32943704	-0.38617717
H	1.26453818	-0.28311691	-1.55339689
H	2.18831163	0.18527443	2.59485652
H	0.39152520	0.10323421	2.09472661
H	3.34723136	0.54567083	-1.65647216
H	3.31591725	-1.21909327	-1.41748832
H	4.74107725	-0.27714523	-0.92289030

UM06-2X Energy = -501.453649287
NIMAG = 1
<S^2> = 1.0503
UM06-2X H = -501.245970945
UM06-2X G (HO) = -501.293798195
UM06-2X G (GR) = -501.293161057

First-Order Saddle Point for (A)–(B) Conformational Interconversion of (*Z*)-5-(2-methoxyallylidene)-6-methylenecyclohexa-1,3-diene

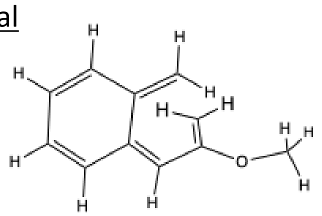

M06-2X Optimized Geometry

C	3.93895211	0.19593048	-0.51745341
C	1.79385743	-0.39612920	0.41450095
C	0.54270815	-1.09204057	0.03333893
C	-0.67207013	-0.54776515	-0.14563648
C	-0.96589234	0.90733829	-0.13006757
C	-2.33469568	1.29493584	0.20535466
C	-3.33359510	0.40340435	0.19119058
C	-3.07565165	-0.98922386	-0.13752860
C	-1.82423814	-1.43293162	-0.31278262
C	-0.07623739	1.83501922	-0.50409708
C	2.02653310	0.00624141	1.66024996
O	2.61876730	-0.29668374	-0.66408607
H	-1.62758102	-2.47883402	-0.51217185
H	-3.91098124	-1.67203942	-0.21408163
H	-4.34906141	0.71356470	0.39685023
H	-2.52447029	2.34304337	0.39954510
H	-0.33842981	2.88493538	-0.47387523
H	0.90487098	1.57307117	-0.87273605
H	0.63679220	-2.16782632	-0.08165874
H	2.92651620	0.50384254	1.98240385
H	1.25518478	-0.15477702	2.39652284
H	4.15909769	0.51782078	0.49531535
H	4.06482996	1.04017999	-1.19384017
H	4.63459771	-0.59452904	-0.79704069

M06-2X Energy = -501.477987916
NIMAG = 1
M06-2X H = -501.266955756
M06-2X G (HO) = -501.316837470
M06-2X G (GR) = -501.315157986

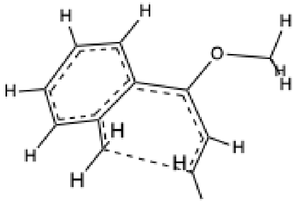

First-Order Saddle Point for Disrotatory Ring Closure of (*Z*)-5-(2-methoxyallylidene)-6-methylenecyclohexa-1,3-diene

M06-2X Optimized Geometry

C	1.56371543	2.31453887	0.24357610
C	1.76936989	0.98245849	0.47033093
C	1.13430740	-0.13563460	-0.11985337
C	-0.25152208	-0.23554946	-0.32838636
C	-1.11966258	0.90103537	-0.18728148
C	-2.42429921	0.66876251	0.35752077
C	-2.93196392	-0.58575654	0.44369663
C	-2.14871301	-1.70261693	0.03275275
C	-0.85223450	-1.53823710	-0.33357076
C	-0.73733848	2.16425557	-0.58555277
C	3.18358878	-1.34387685	0.09397682
O	1.79063873	-1.32874569	-0.13478331
H	1.88817026	3.01471359	1.00527558
H	-0.14282272	2.26183367	-1.47440746
H	-0.23674512	-2.39212622	-0.57131906
H	-2.58295282	-2.69298435	0.04967038
H	-3.94545355	-0.74051075	0.78823439
H	-3.03293762	1.53130527	0.59922139
H	-1.35083090	3.01838709	-0.32561009
H	3.54450341	-2.29725724	-0.28286166
H	3.41486355	-1.27481959	1.15884725
H	3.67705231	-0.52481600	-0.43257711
H	2.38888294	0.71862759	1.32369912
H	1.47430750	2.73202056	-0.73744956

M06-2X Energy	= -501.463291879
NIMAG	= 1
M06-2X H	= -501.251944252
M06-2X G (HO)	= -501.299051054
M06-2X G (GR)	= -501.298445442

First-Order Saddle Point for Conrotatory Ring Closure of (*Z*)-5-(2-methoxyallylidene)-6-methylenecyclohexa-1,3-diene

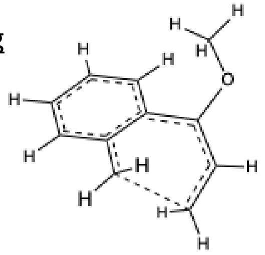

UM06-2X Optimized Geometry

C	-1.26528295	2.24594619	-0.90762428
C	-1.96426302	1.06288740	-0.59407371
C	-1.34221825	-0.10575856	-0.26620003
C	0.12431537	-0.20677287	-0.13537763
C	0.85396724	0.82618229	0.50893970
C	0.19297848	1.95033440	1.12433522
C	2.24993173	0.73653505	0.51387526
C	2.90940706	-0.34580186	-0.04945794
C	2.18510145	-1.36886122	-0.64263887
C	0.79783322	-1.28633230	-0.68811110
C	-1.87892644	-1.97916702	1.06229147
O	-2.09036486	-1.23742011	-0.13049391
H	-1.79906535	3.18209345	-0.97686634
H	-0.77861171	1.83825822	1.57476245

H	0.79029365	2.79890922	1.42679944
H	0.22072881	-2.06171414	-1.17686577
H	2.69186416	-2.21810643	-1.07939254
H	3.98960571	-0.39109058	-0.01897590
H	2.81841991	1.52327589	0.99363995
H	-2.55970938	-2.82499343	1.02382537
H	-2.10810500	-1.36369207	1.93571959
H	-0.85213375	-2.33864689	1.13736578
H	-3.04676826	1.07283390	-0.54691120
H	-0.27830319	2.21380654	-1.34046343

UM06-2X Energy = -501.449234058
NIMAG = 1
$\langle S^2 \rangle$ = 0.8807
UM06-2X H = -501.240842998
UM06-2X G (HO) = -501.288171849
UM06-2X G (GR) = -501.287777099

4-Methoxy-1,2-dihydronaphthalene

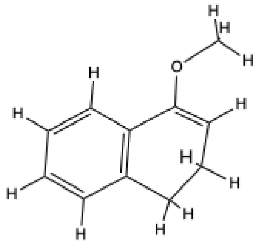

M06-2X Optimized Geometry

C	-2.17410690	-1.74007919	-0.16120087
C	-2.99740274	-0.63157776	-0.03474828
C	-2.43871697	0.63075432	0.11813497
C	-1.06291929	0.80135232	0.14111763
C	-0.23523112	-0.31964492	0.00406621
C	-0.79620188	-1.58456866	-0.14107303
C	-0.42591688	2.14610145	0.36768995
C	0.87430561	2.28542502	-0.41539076
C	1.76233919	1.08859467	-0.20084449
C	1.22596306	-0.12013088	-0.00229915
C	3.32002581	-1.19207296	0.12904088
O	1.91525800	-1.27746272	0.16486288
H	-2.60370571	-2.72572623	-0.27832553
H	-4.07264581	-0.74782670	-0.05344903
H	-3.08027713	1.49720745	0.22329646
H	-0.14408609	-2.44031804	-0.23958808
H	-0.20155396	2.24295185	1.43556740
H	-1.12515121	2.94192130	0.11105649
H	1.39316194	3.19388139	-0.11017684
H	0.65274280	2.39632950	-1.48309943
H	2.83190210	1.22327458	-0.26181803
H	3.69610245	-2.19817401	0.28967989
H	3.69049783	-0.53136053	0.91711138
H	3.66616751	-0.82064115	-0.83888056

M06-2X Energy = -501.568600147
NIMAG = 1
M06-2X H = -501.353195021
M06-2X G (HO) = -501.398964753
M06-2X G (GR) = -501.398684126

Data for main text Figure 9

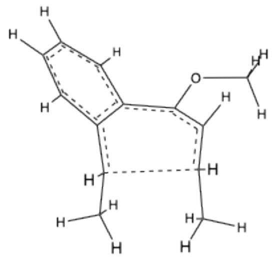

First-Order Saddle Point for Disrotatory Ring Closure of (5*Z*,6*E*)-5-Ethylidene-6-((*Z*)-1-methoxybut-2-en-1-ylidene) cyclohexa-1,3-diene

M06-2X Optimized Geometry

C	-0.93841583	-0.93607939	-0.18403032
C	-0.55907495	0.35480901	0.33497710
C	0.70857704	0.89566480	0.06047489
C	1.59392883	0.31079546	-0.88062550
C	2.05739469	-0.97525423	-0.93196511
C	-1.61180158	1.26179653	0.69414335
C	-2.88285875	1.03951371	0.26970822
C	-3.18087285	-0.08297516	-0.55206871
C	-2.22859390	-1.02399017	-0.79105528
C	-0.14244635	-2.06518032	-0.07108845
C	2.85097264	-1.62808409	0.16151775
C	0.35667894	-2.50129290	1.27843986
C	2.05248978	2.86273999	0.04651818
O	0.83132824	2.21882497	0.34799714
H	1.74619801	0.94202412	-1.75532898
H	2.35513363	-1.31599945	-1.92108294
H	-2.47235418	-1.92797242	-1.33616511
H	-4.18130829	-0.21217333	-0.94214947
H	-3.66624869	1.74265609	0.51804072
H	-1.36137815	2.16292106	1.23314520
H	-0.41341520	-2.89100534	-0.72518903
H	2.57077588	-1.27737878	1.15047586
H	3.89597524	-1.34668851	-0.00107908
H	2.81127981	-2.71427745	0.12911705
H	0.59185445	-1.65558387	1.92038135
H	1.20777157	-3.17502506	1.24430680
H	-0.45759512	-3.05612688	1.75616149
H	2.07244383	3.77288162	0.64047199
H	2.11353333	3.12787051	-1.01026092
H	2.90255527	2.22818923	0.30418927

M06-2X Energy = -580.057862831
NIMAG = 1
M06-2X H = -579.787468710
M06-2X G (HO) = -579.840174331
M06-2X G (GR) = -579.839277193

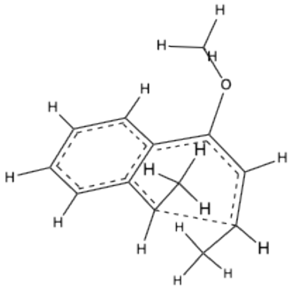

First-Order Saddle Point for Conrotatory Ring Closure of (5*Z*,6*E*)-5-Ethylidene-6-((*Z*)-1-methoxybut-2-en-1-ylidene) cyclohexa-1,3-diene

UM06-2X Optimized Geometry

C	0.25805809	-1.61768237	1.19876271
C	-0.72018171	-0.73430248	0.56570946
C	-0.35914029	0.43363086	-0.15110523
C	1.04977696	0.75699641	-0.42401194
C	2.00804566	-0.17836828	-0.69884419
C	1.77833982	-1.54134069	-0.96660419

C	-1.35605518	1.25485399	-0.67199417
C	-2.69897200	0.93303343	-0.54127787
C	-3.05800525	-0.23896812	0.10714916
C	-2.07621947	-1.05633123	0.64921877
C	0.65355530	-2.09776713	-1.78459488
C	1.22050493	-1.13163634	2.22733995
C	1.12818801	2.80370241	0.75682949
O	1.41636047	2.07638871	-0.42716506
H	2.62817121	-2.20211657	-0.83996596
H	3.03439556	0.16927690	-0.63272162
H	-2.36181532	-1.95819205	1.17635906
H	-4.09967440	-0.51512652	0.19925316
H	-3.45516405	1.58219581	-0.96080201
H	-1.05741808	2.14021580	-1.21951526
H	-0.01255834	-2.66639704	1.23768015
H	2.03210206	-1.84291080	2.37854337
H	0.71641473	-0.99547439	3.19151461
H	1.64910016	-0.16953634	1.95037742
H	-0.11351282	-1.35642226	-1.99307703
H	0.17758356	-2.95778859	-1.30944422
H	1.04932651	-2.44472291	-2.74374247
H	1.45979269	3.82385252	0.58207981
H	1.67624502	2.38391538	1.60443566
H	0.06122670	2.80065771	0.98435702

UM06-2X Energy = -580.063705103
NIMAG = 1
<S^2> = 0.9437
UM06-2X H = -579.795966913
UM06-2X G (HO) = -579.851561888
UM06-2X G (GR) = -579.850401817

First-Order Saddle Point for Disrotatory Ring Closure of (5*Z*,6*E*)-5-Ethylidene-6-((*E*)-1-methoxybut-2-en-1-ylidene) cyclohexa-1,3-diene

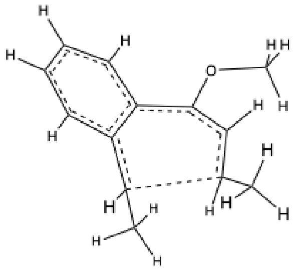

M06-2X Optimized Geometry

C	2.93908679	-1.73851440	-1.31153439
C	2.08448417	-1.07433570	-0.27144790
C	1.73290840	0.24547084	-0.39114109
C	0.64540186	0.94283090	0.17774125
C	-0.65698507	0.42240249	0.27444214
C	-0.91747500	-0.97177230	0.04673991
C	-2.11850608	-1.29980207	-0.65816429
C	-3.11435841	-0.38824732	-0.80665160
C	-2.95631001	0.93096172	-0.29662893
C	-1.76510718	1.32943093	0.22040260
C	-0.08652883	-1.97688335	0.51702083
C	0.42148405	-1.98565543	1.93476860
C	1.97068701	2.93145608	0.16661280
O	0.71518493	2.29928935	0.28706104
H	-1.61335956	2.35321206	0.52667332
H	-3.77802432	1.63108098	-0.36205167
H	-4.04721490	-0.67432454	-1.27346541
H	-2.25986568	-2.32727035	-0.97101143
H	0.67030759	-0.98580861	2.28573602

H	1.29415018	-2.62795217	2.06380263
H	-0.36049428	-2.38569228	2.58675150
H	-0.26937944	-2.96623003	0.10389830
H	1.85472385	3.92262228	0.59742365
H	2.27000060	3.03392899	-0.87863695
H	2.74268269	2.37944528	0.70615969
H	2.25583820	0.81655914	-1.15609832
H	2.09779237	-1.54521834	0.68851935
H	3.95167098	-1.91050950	-0.93874726
H	2.99681594	-1.14453556	-2.22238358
H	2.52878608	-2.71409475	-1.57542315

M06-2X Energy = -580.079193097
NIMAG = 1
M06-2X H = -579.808949832
M06-2X G (HO) = -579.862909611
M06-2X G (GR) = -579.861936790

First-Order Saddle Point for Conrotatory Ring Closure of (5*Z*,6*E*)-5-Ethylidene-6-((*E*)-1-methoxybut-2-en-1-ylidene) cyclohexa-1,3-diene

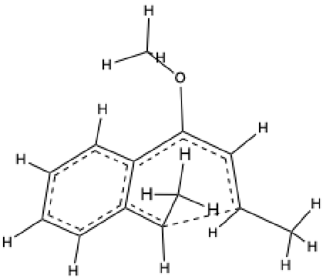

UM06-2X Optimized Geometry

C	3.53152471	-0.99646622	-1.05709294
C	2.11101174	-0.54644713	-0.97704683
C	1.75581825	0.74933135	-0.60065850
C	0.47597617	1.14802892	-0.30701100
C	-0.66210143	0.22134795	-0.22103078
C	-0.52586428	-1.07862232	0.32818711
C	0.71610618	-1.55845910	0.94186220
C	1.33867968	-0.87088298	2.10787433
C	-1.62844302	-1.93230367	0.27342821
C	-2.84541303	-1.52530403	-0.25581732
C	-2.98440326	-0.23789580	-0.75029755
C	-1.89456414	0.61950595	-0.73562446
C	-0.39194465	2.87869025	1.04628970
O	0.23207675	2.48678955	-0.16668792
H	2.37267490	-1.18674949	2.24964882
H	0.79314041	-1.10245574	3.03026448
H	1.31959852	0.21196305	1.98695977
H	0.91815215	-2.61703275	0.82810996
H	-1.97809427	1.61433639	-1.15494657
H	-3.92858437	0.09387477	-1.15998177
H	-3.68123047	-2.21144340	-0.27435029
H	-1.52861788	-2.93125501	0.67957787
H	-0.52721602	3.95569818	0.99159049
H	0.24720836	2.63475052	1.89871964
H	-1.36156048	2.39554599	1.17664001
H	2.53454773	1.49545229	-0.47624463
H	1.36403449	-1.18764235	-1.42240110
H	4.20522738	-0.26558068	-0.61115549
H	3.84177377	-1.15684214	-2.09381846

H 3.67561621 -1.94762997 -0.53918139

UM06-2X Energy = -580.068902034
NIMAG = 1
<S^2> = 0.9188
UM06-2X H = -579.801105516
UM06-2X G (HO) = -579.856727533
UM06-2X G (GR) = -579.855677686

Data for main text Figure 10

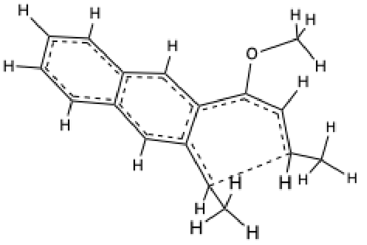

First-Order Saddle Point for Disrotatory Ring Closure of (2*Z*,3*E*)-2-Ethylidene-3-((*E*)-1-methoxybut-2-en-1-ylidene)-2,3-dihydronaphthalene

M06-2X Optimized Geometry

C	3.94392455	-1.42017725	-1.62314418
C	3.15095022	-0.84125796	-0.49022749
C	2.60482800	0.40827809	-0.57717249
C	1.53194703	0.99835460	0.13171379
C	0.31935901	0.34849578	0.43038353
C	0.18972335	-1.08846285	0.30295021
C	-1.03778784	-1.57929928	-0.20660277
C	-2.16642445	-0.79384000	-0.25049380
C	-2.08994420	0.57738741	0.19509165
C	-0.86540764	1.11922855	0.51627331
C	-3.29546861	1.35526649	0.21946563
C	-4.47687839	0.82479815	-0.16931512
C	-4.55172650	-0.53589943	-0.61610076
C	-3.44418496	-1.31012459	-0.65374458
C	1.17516689	-1.96799474	0.72158590
C	1.90663003	-1.80133594	2.02431150
C	2.61994589	3.11664267	-0.08686183
O	1.46657422	2.35640871	0.20291943
H	-0.79418005	2.17389689	0.73920385
H	-3.49913364	-2.33980103	-0.98460881
H	-5.50763074	-0.93822389	-0.92302659
H	-5.37699456	1.42387239	-0.14853786
H	-3.23224076	2.38370962	0.55254969
H	-1.11558398	-2.63614424	-0.43619122
H	2.06948908	-0.75452101	2.27288287
H	2.86995180	-2.31406154	2.03700970
H	1.30839668	-2.24944046	2.82355691
H	1.04855741	-2.99979082	0.40423044
H	2.44916911	4.10157701	0.33952384
H	2.77228197	3.22002161	-1.16295318
H	3.50917680	2.66748472	0.35884470
H	2.94386939	1.01859578	-1.41234965
H	3.29224740	-1.31632895	0.45635379
H	4.98885271	-1.56687194	-1.34170366
H	3.90296322	-0.78592815	-2.50742103
H	3.55030006	-2.39967383	-1.89939412

M06-2X Energy = -733.701607665
NIMAG = 1
M06-2X H = -733.381388877

M06-2X G (HO) = -733.441745535
M06-2X G (GR) = -733.440218778

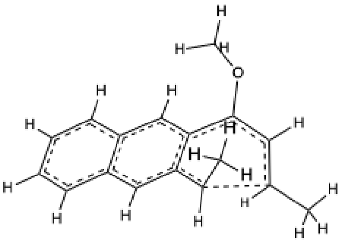

First-Order Saddle Point for Conrotatory Ring Closure of (2*Z*,3*E*)-2-Ethylidene-3-((*E*)-1-methoxybut-2-en-1-ylidene)-2,3-dihydronaphthalene

UM06-2X Optimized Geometry

C	4.03529042	-1.60178741	-1.52681440
C	2.77381389	-0.86225998	-1.23050987
C	2.76406025	0.45236796	-0.77901415
C	1.65133860	1.10081039	-0.29402940
C	0.36591683	0.43373647	-0.05934373
C	0.29975519	-0.91790669	0.45387956
C	1.48256755	-1.64646127	0.90072796
C	2.41101516	-1.11917796	1.93970795
C	-0.92955500	-1.52752281	0.51454274
C	-2.13215130	-0.86882537	0.15442143
C	-2.06557022	0.47528936	-0.28392572
C	-0.79280966	1.08773617	-0.38892450
C	-3.25800372	1.14715148	-0.62947642
C	-4.46775360	0.51186465	-0.54033833
C	-4.53489406	-0.82538082	-0.10147854
C	-3.39216390	-1.50002445	0.23701404
C	1.37848843	2.93319384	1.17305342
O	1.72232066	2.45490999	-0.11867738
H	2.58024715	-0.04956376	1.82485026
H	3.37420354	-1.62962871	1.90735683
H	1.99116772	-1.27319412	2.94065489
H	1.43517649	-2.72360647	0.79211667
H	-0.72866726	2.09560460	-0.78286411
H	-3.44014945	-2.52758547	0.57523025
H	-5.49521159	-1.31806041	-0.03326624
H	-5.37626319	1.03358090	-0.80826883
H	-3.19824075	2.17330973	-0.97004133
H	-0.99494758	-2.54320715	0.88771822
H	1.44441912	4.01697006	1.13044747
H	2.08404424	2.55485837	1.91689041
H	0.36701336	2.63920709	1.45600419
H	3.69561271	1.00928974	-0.75561885
H	1.84776493	-1.31776082	-1.54958440
H	4.12069254	-1.83485123	-2.59153574
H	4.06732270	-2.55637452	-0.99566439
H	4.91118089	-1.02508907	-1.23195853

UM06-2X Energy = -733.703877435
NIMAG = 1
<S^2> = 0.9001
UM06-2X H = -733.386096482
UM06-2X G (HO) = -733.448066790
UM06-2X G (GR) = -733.446395560

Data for main text Scheme 10

Results in this section used the aug-cc-pVTZ basis set.

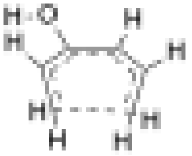

First-Order Saddle Point for Disrotatory
Ring Closure of (*E*)-1,3,5-Hexatrien-3-ol

M06-2X Optimized Geometry

C	0.85937039	1.65351848	0.03837554
C	-0.42187787	1.20515317	-0.24583519
C	-0.92623685	-0.04927106	0.10661462
C	-0.15437521	-1.21212658	0.07332114
C	1.20340821	-1.22129715	-0.21352006
C	2.08454881	-0.21344796	0.15092060
O	-2.27821907	-0.26448745	0.09573762
H	3.05060750	-0.16516673	-0.33797683
H	2.09999395	0.08589839	1.18123071
H	1.55087907	-2.01006108	-0.87381333
H	-0.73123515	-2.12002626	-0.05754298
H	-2.73225612	0.58022983	0.13610751
H	-1.02424347	1.74658465	-0.97125415
H	1.26082361	2.44902846	-0.58018851
H	1.19079383	1.69591665	1.05709529

M06-2X Energy = -308.550557391
NIMAG = 1
M06-2X H = -308.418650610
M06-2X G (HO) = -308.456498474
M06-2X G (GR) = -308.456207922

First-Order Saddle Point for Disrotatory
Ring Closure of (*E*)-1,3,5-Hexatrien-3-oxide

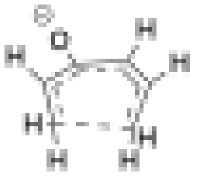

M06-2X Optimized Geometry

C	-0.83791267	1.62048087	0.01171006
C	0.46253423	1.18435202	0.26062999
C	1.04640285	-0.06771340	-0.15436497
C	0.15576098	-1.21144749	0.04007238
C	-1.19538173	-1.19374044	0.28521135
C	-2.05322318	-0.15478822	-0.13242820
O	2.27966312	-0.24234969	-0.30324297
H	-3.04091535	-0.10154422	0.31972012
H	-2.06757928	0.06670422	-1.18610573
H	-1.58762803	-1.96100419	0.95318593
H	0.71619579	-2.10177807	0.31201566
H	1.04430338	1.67767569	1.03310960
H	-1.23144267	2.38711102	0.68020761
H	-1.17665169	1.76988570	-1.00045402

M06-2X Energy = -307.983838897
NIMAG = 1
M06-2X H = -307.866493470
M06-2X G (HO) = -307.903824400
M06-2X G (GR) = -307.903532089

First-Order Saddle Point for Conrotatory
Ring Closure of (*E*)-1,3,5-Hexatrien-3-ol

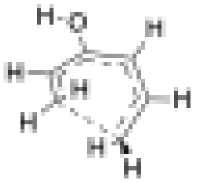

UM06-2X Optimized Geometry

C	1.94018823	0.15900874	-0.61532382
C	1.27852993	1.15040090	0.16950709
C	-0.06333663	1.15933773	0.41170606
C	-0.91615539	0.05917493	0.01553027
C	-0.47917346	-1.23882621	-0.03752070
C	0.79593693	-1.64846211	0.44135044
O	-2.19260201	0.42225128	-0.29249118
H	1.16784966	-2.62795633	0.17836361
H	1.21426516	-1.19507753	1.32683900
H	-1.11968993	-1.97330311	-0.51792485
H	-2.72835609	-0.36514014	-0.42502428
H	-0.52780727	1.99295639	0.92148400
H	1.88558572	1.92153933	0.62972578
H	3.01619351	0.07364573	-0.56958636
H	1.45641382	-0.24918882	-1.48892740

UM06-2X Energy = -308.514354370
NIMAG = 1
$\langle S^2 \rangle$ = 0.9849
UM06-2X H = -308.386643075
UM06-2X G (HO) = -308.424576533
UM06-2X G (GR) = -308.424565705

<u>First-Order Saddle Point for Conrotatory Ring Closure of (*E*)-1,3,5-Hexatrien-3-oxide</u>

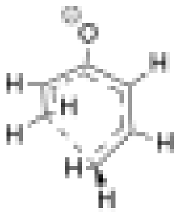

UM06-2X Optimized Geometry

C	-0.81090366	1.47716825	0.42543492
C	0.45933147	1.26887895	-0.08522599
C	1.05078333	-0.05197498	0.03099379
C	0.11591892	-1.13581414	0.46471544
C	-1.19846649	-1.13534063	0.11323855
C	-1.95304294	-0.12439553	-0.59032578
O	2.21642934	-0.31064963	-0.27400274
H	-1.32420876	2.40841375	0.20811612
H	-1.53853548	0.31842369	-1.48420351
H	-3.01913680	-0.33479367	-0.67329306
H	-1.77935953	-1.97495085	0.49744544
H	0.53856952	-1.95605656	1.03081771
H	0.86431461	1.91784438	-0.85231017
H	-1.09911681	0.99690488	1.34951145

UM06-2X Energy = -307.975037287
NIMAG = 1
$\langle S^2 \rangle$ = 0.0000
UM06-2X H = -307.858688782
UM06-2X G (HO) = -307.895487371
UM06-2X G (GR) = -307.895470537

Data for main text Scheme 11

Results in this section used the SMD solvation model for DMSO.

<u>7-Alkoxy-7-vinylbicyclo[4.2.0]octa-1,3,5-triene anion with a Tetramethylammonium Counterion</u>

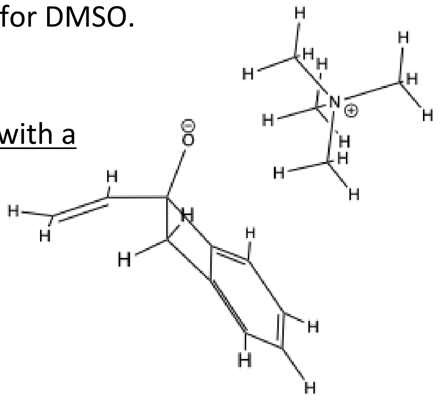

M06-2X Optimized Geometry

C	-1.18102616	1.16540355	-0.00257691
C	-1.23824916	-0.34559846	-0.40500191
C	-1.63488416	-0.75572046	0.86027009
C	-1.81719416	-2.09018747	1.17690709
C	-1.56929116	-3.01279147	0.15519209
C	-1.15680816	-2.60336447	-1.11246092
C	-0.97863716	-1.25013947	-1.42006992
C	-1.64841516	0.63784954	1.44681810
C	-2.23734417	1.95438755	-0.75156092
C	-3.34948317	2.51236655	-0.28759591
O	0.01407885	1.76539755	-0.07597991
H	-0.65063116	-0.95027046	-2.40878492
H	-0.97352016	-3.35448848	-1.87114992
H	-1.69373616	-4.07065048	0.35011409
H	-2.12446717	-2.42773347	2.15938510
H	-0.86388016	0.82372254	2.18147510
H	-2.60408117	0.97983954	1.84636110
H	-3.66241217	2.41206455	0.74505909
H	-3.98948417	3.10543556	-0.93093192
H	-1.97244516	2.10760655	-1.79689792
N	2.98160286	0.24521354	0.06682809
C	4.32689187	-0.38221246	0.06804109
C	2.05590086	-0.52519546	0.94511109
C	2.42835286	0.27137854	-1.31802592
C	3.06588486	1.64462055	0.57602909
H	4.70286187	-0.39328946	1.08782109
H	4.23646987	-1.39622547	-0.31333391
H	4.98136987	0.20551354	-0.57032891
H	1.10987985	0.01330054	0.94625009
H	1.94216485	-1.52646747	0.53471209
H	2.48906786	-0.57153546	1.94134810
H	3.11816386	0.83084054	-1.94591592
H	2.34685786	-0.75425746	-1.67059492
H	1.45497285	0.76255254	-1.25559892
H	3.73648186	2.20297655	-0.07268991
H	2.05560486	2.05301755	0.55332909
H	3.46177686	1.61432455	1.58835910

M06-2X Energy = -675.944655066
NIMAG = 0
M06-2X H = -675.601273759
M06-2X G (HO) = -675.665007083
M06-2X G (GR) = -675.661955268

First Order Saddle Point for Conrotatory Inward Ring Opening of 7-Alkoxy-7-vinylbicyclo[4.2.0]octa-1,3,5-triene anion with a Tetramethyl-ammonium Counterion

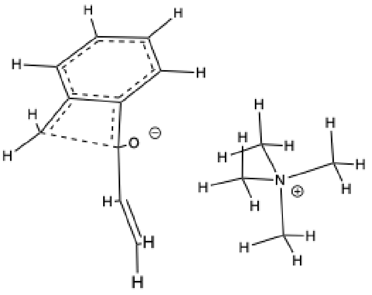

M06-2X Optimized Geometry

C	0.72591598	0.97320360	0.37182596
C	1.67501698	-0.18790440	0.18129996
C	2.92496599	0.41874760	0.26095996
C	4.06893299	-0.28951940	-0.10780004

C	3.92319399	-1.63677141	-0.42742504
C	2.67509399	-2.26433141	-0.38487204
C	1.52299198	-1.54258541	-0.07638504
C	2.59841099	1.76900861	0.73392596
C	0.49847198	1.74286861	-0.89285504
C	-0.59722203	2.47073561	-1.10819305
O	-0.15705803	0.97577260	1.27317597
H	0.55314198	-2.02643141	-0.09011004
H	2.60379599	-3.31782242	-0.62433804
H	4.79518800	-2.21974341	-0.69759204
H	5.04898500	0.17368660	-0.11449104
H	3.16112099	2.62236361	0.35220196
H	2.40887799	1.83792661	1.80123297
H	1.27125998	1.66843761	-1.65137605
H	-1.36033703	2.54505361	-0.34214704
H	-0.74159503	3.02490261	-2.02710805
N	-3.45158804	-0.50524440	0.00627896
C	-2.14997304	-1.18668341	-0.23988904
C	-3.80480104	0.32439160	-1.17775505
C	-3.32504704	0.36704460	1.20852797
C	-4.51463405	-1.52108241	0.23183096
H	-2.25690404	-1.82797541	-1.11091305
H	-1.39349203	-0.42211540	-0.39885704
H	-1.90318103	-1.77527041	0.64021296
H	-4.76011005	0.80642060	-0.98708205
H	-3.02412004	1.06721261	-1.32355105
H	-3.87435604	-0.32631840	-2.04550905
H	-3.13399104	-0.27149640	2.06740497
H	-2.48161404	1.03789660	1.05846396
H	-4.25926505	0.90754660	1.33665097
H	-4.23862505	-2.12186841	1.09471296
H	-5.45258905	-1.00305841	0.41348096
H	-4.58815605	-2.14343641	-0.65605004

M06-2X Energy = -675.931419480
NIMAG = 1
M06-2X H = -675.590141078
M06-2X G (HO) = -675.654249795
M06-2X G (GR) = -675.651093499

<u>First Order Saddle Point for Internal Rotation of (*E*)-1-(6-methylene-cyclohexa-2,4-dien-1-ylidene)prop-2-en-1-ol anion with a Tetramethylammonium Counterion</u>

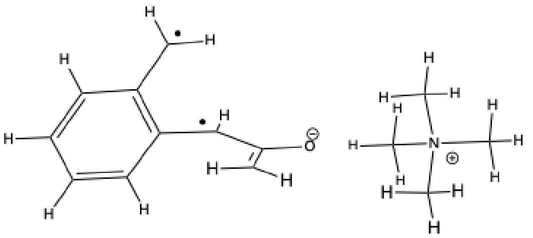

UM06-2X Optimized Geometry

C	-0.02964015	0.00621886	0.62997832
C	-0.96944337	-0.22603036	-0.44759588
C	-2.43796549	-0.27316090	-0.30495508
C	-3.22453834	0.91978588	-0.35108577
C	-4.63255565	0.80107798	-0.23813882
C	-5.24115988	-0.42467147	-0.07698537
C	-4.46635099	-1.58277870	-0.01960211
C	-3.08175399	-1.49215953	-0.13322897
C	-2.63518842	2.18927724	-0.48385925
C	-0.56187820	0.24279098	1.89281143

O	1.23180781	-0.00585398	0.36068861
H	-2.48219573	-2.39382206	-0.09273656
H	-4.93578607	-2.54893193	0.10951567
H	-6.31825106	-0.48717710	0.00760866
H	-5.23008107	1.70409385	-0.27717721
H	-3.25518592	3.07457466	-0.51289311
H	-1.56575654	2.31114865	-0.56372222
H	-0.55074755	-0.35898779	-1.44015789
H	0.09862645	0.43701217	2.72936897
H	-1.62819437	0.24334468	2.07146680
N	4.51262455	-0.05059266	-0.18268901
C	3.80096366	-0.81332580	-1.24779440
C	4.01167065	1.35306030	-0.17333953
C	5.97541556	-0.06796605	-0.43913261
C	4.21960402	-0.67895567	1.13713354
H	2.73746819	-0.77468072	-1.01550280
H	4.01474200	-0.34310067	-2.20465225
H	4.17259507	-1.83502943	-1.23932904
H	4.53337242	1.89574058	0.61114061
H	4.22240638	1.79651742	-1.14350718
H	2.94064336	1.31075938	0.01852131
H	6.31203708	-1.10131893	-0.43935086
H	6.16079506	0.39084160	-1.40706745
H	6.47230544	0.49202821	0.34889024
H	4.57632399	-1.70556365	1.11099880
H	4.74403611	-0.11769637	1.90667222
H	3.14106294	-0.63621049	1.28156988

UM06-2X Energy = -675.909523981
NIMAG = 1
<S^2> = 1.0447
UM06-2X H = -675.571168083
UM06-2X G (HO) = -675.636802969
UM06-2X G (GR) = -675.633080848

First Order Saddle Point for Conrotatory Ring Closure of (*E*)-1-(6-Methylenecyclohexa-2,4-dien-1-ylidene)prop-2-en-1-ol Anion with a Tetramethylammonium Counterion

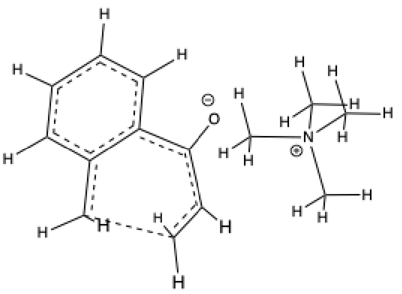

M06-2X Optimized Geometry

C	-0.63499633	-1.79637093	-1.48931908
C	-0.29207820	-2.11366934	-0.19877474
C	-0.62179522	-1.20393458	0.86972871
C	-1.47477556	-0.01577405	0.49297040
C	-2.58368636	-0.15983258	-0.40189254
C	-2.93983348	-1.38404059	-1.05949935
C	-3.27162261	1.04353328	-0.69593452
C	-2.90262562	2.26973225	-0.16732670
C	-1.82684953	2.37938481	0.70925529
C	-1.12641169	1.21620889	1.02140385
O	-0.28447824	-1.37275008	2.04194592
H	-3.83127251	-1.31973519	-1.68135001
H	-2.82248968	-2.32849622	-0.55176846
H	-0.28068251	1.25515888	1.69998604
H	-1.54681175	3.33140636	1.13886590
H	-3.47367280	3.15115029	-0.43521877

H	-4.12729274	0.99394671	-1.36020210
H	-0.03825551	-3.13001418	0.07810332
H	-0.66331830	-0.77026387	-1.82437367
H	-0.53287914	-2.53919044	-2.27325121
N	3.16039229	0.27636704	-0.22176061
C	2.02364676	1.01454802	-0.83908267
C	4.38212539	1.12618669	-0.25820942
C	2.82560612	-0.05224308	1.19213922
C	3.40501074	-0.98199755	-0.97889924
H	2.28026170	1.23921683	-1.87126116
H	1.13974943	0.38194193	-0.79435023
H	1.86683558	1.93185216	-0.27765392
H	4.17995912	2.03842616	0.29649617
H	5.19787707	0.57490690	0.20173247
H	4.61253907	1.35355019	-1.29565517
H	1.93229853	-0.67319136	1.20368288
H	3.66698287	-0.58532444	1.62725719
H	2.65069643	0.87867917	1.72569474
H	3.65771340	-0.72185359	-2.00343655
H	4.22787379	-1.51113607	-0.50613485
H	2.49717004	-1.58019478	-0.94984610

M06-2X Energy = -675.937240751
NIMAG = 1
M06-2X H = -675.595974350
M06-2X G (HO) = -675.659362448
M06-2X G (GR) = -675.656106887

First Order Saddle Point for Disrotatory Ring Closure of (1*E*,2*E*)-1-((*Z*)-6-Ethylidene-cyclohexa-2,4-dien-1-ylidene) but-2-en-1-ol Anion with a Tetramethylammonium Counterion

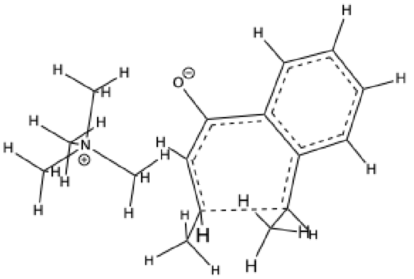

M06-2X Optimized Geometry

C	0.71885993	3.10347251	-1.87723933
C	0.47793804	2.01141577	-0.86749360
C	0.10616695	0.75164040	-1.28509524
C	0.18829900	-0.51196779	-0.58873710
C	1.49115952	-0.81196753	0.01585318
C	2.46845192	0.18198192	0.34546781
C	3.83866813	-0.19590808	0.22565339
C	4.21446496	-1.50263969	0.06851841
C	3.23194118	-2.51688810	-0.02016338
C	1.90771699	-2.16599411	-0.06134363
C	2.11352450	1.47528231	0.76266455
C	1.12491486	1.67079634	1.88647608
O	-0.65913096	-1.42346182	-0.78598443
H	1.14498639	-2.91510906	-0.22852155
H	3.52863775	-3.55479951	-0.10054644
H	5.26514778	-1.76461980	0.04406195
H	4.59297338	0.57395135	0.35098066
H	0.29012396	0.97175718	1.82054714
H	0.70825859	2.68082744	1.90606827
H	1.61162498	1.51869152	2.85670794
H	2.94037357	2.18350981	0.79216336

H	-0.15377692	0.62041363	-2.33531141
H	0.15083600	2.38170522	0.08758955
H	-0.19352327	3.68028597	-2.05551843
H	1.06040130	2.70228922	-2.83164042
H	1.46927755	3.80936399	-1.51804437
N	-3.71176405	-0.10655494	0.41413876
C	-2.63271885	0.44695296	1.27953824
C	-3.42880088	0.24024224	-1.00828632
C	-3.73588214	-1.58958490	0.55484812
C	-5.02340111	0.46350202	0.82054766
H	-2.47527457	-0.21394171	-1.27430014
H	-4.23565552	-0.15624461	-1.61991838
H	-3.38741127	1.32343185	-1.09282086
H	-3.92566376	-1.83272197	1.59727142
H	-4.53153065	-1.97869449	-0.07543631
H	-2.76779516	-1.96793287	0.23403733
H	-4.97415426	1.54425066	0.71609716
H	-5.79109111	0.05332559	0.16980189
H	-5.21376520	0.19003914	1.85508945
H	-2.58954020	1.52336039	1.13223589
H	-2.86971579	0.21297360	2.31441570
H	-1.69803216	-0.02040296	0.98384415

M06-2X Energy = -754.552066457
NIMAG = 1
M06-2X H = -754.150879050
M06-2X G (HO) = -754.220028992
M06-2X G (GR) = -754.216789291

First Order Saddle Point for Conrotatory Ring Closure of (1*E*,2*E*)-1-((*Z*)-6-Ethylidene-cyclohexa-2,4-dien-1-ylidene) but-2-en-1-ol Anion with a Tetramethylammonium Counterion

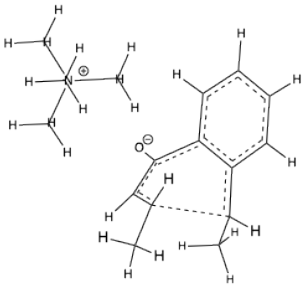

M06-2X Optimized Geometry

C	0.50350205	3.11353274	-1.43011002
C	0.55897937	1.80747376	-0.70143087
C	0.21803266	1.71958310	0.62825357
C	0.50374803	0.52260459	1.38225828
C	1.24198349	-0.56624505	0.64039014
C	2.29799756	-0.27322553	-0.28209703
C	2.86222141	1.03338714	-0.44623415
C	3.23699459	1.86948888	0.74456665
C	2.69585912	-1.35738251	-1.10323296
C	2.15911790	-2.62734908	-0.98050766
C	1.18806206	-2.90614534	-0.02028700
C	0.73520810	-1.85258785	0.76757754
O	0.18849064	0.36776511	2.56207830
H	3.68664805	1.29017669	1.56391938
H	2.37961458	2.39034768	1.19617985
H	3.96557309	2.63287746	0.46404498
H	3.57434482	1.08676955	-1.26748088
H	-0.05977451	-2.01386839	1.48841841
H	0.78656694	-3.90337864	0.09805631

H	2.51714287	-3.41677339	-1.63124073
H	3.47284944	-1.18222570	-1.83962307
H	0.01247686	2.61676399	1.20230538
H	0.52169596	0.92098686	-1.31865750
H	0.85960028	3.93000718	-0.79959708
H	-0.52929521	3.34592927	-1.71257718
H	1.09345984	3.09136968	-2.34516203
N	-3.29329384	-0.30439057	-0.35649654
C	-2.17267609	-1.01318171	-1.03532698
C	-4.57765724	-0.98642535	-0.67392430
C	-3.06639108	-0.32300433	1.11588968
C	-3.34787378	1.10525113	-0.83373316
H	-2.34888482	-0.98375735	-2.10755376
H	-1.24600098	-0.50187237	-0.78379739
H	-2.15141824	-2.04024132	-0.68022389
H	-4.51891302	-2.01115422	-0.31711861
H	-5.38253506	-0.45483080	-0.17344155
H	-4.71879151	-0.96558850	-1.75126185
H	-2.12513336	0.18084391	1.32583155
H	-3.89151087	0.19539217	1.59712289
H	-3.02754610	-1.35915032	1.44178209
H	-3.51788645	1.09611952	-1.90711454
H	-4.16404264	1.60972347	-0.32364946
H	-2.39752891	1.58043952	-0.60011721

M06-2X Energy = -754.554574277
NIMAG = 1
M06-2X H = -754.154411244
M06-2X G (HO) = -754.225817786
M06-2X G (GR) = -754.221700969

References

1 Gaussian 09, Revision D.01, M. J. Frisch, G. W. Trucks, H. B. Schlegel, G. E. Scuseria, M. A. Robb, J. R. Cheeseman, G. Scalmani, V. Barone, B. Mennucci, G. A. Petersson, H. Nakatsuji, M. Caricato, X. Li, H. P. Hratchian, A. F. Izmaylov, J. Bloino, G. Zheng, J. L. Sonnenberg, M. Hada, M. Ehara, K. Toyota, R. Fukuda, J. Hasegawa, M. Ishida, T. Nakajima, Y. Honda, O. Kitao, H. Nakai, T. Vreven, J. A. Montgomery, Jr., J. E. Peralta, F. Ogliaro, M. Bearpark, J. J. Heyd, E. Brothers, K. N. Kudin, V. N. Staroverov, T. Keith, R. Kobayashi, J. Normand, K. Raghavachari, A. Rendell, J. C. Burant, S. S. Iyengar, J. Tomasi, M. Cossi, N. Rega, J. M. Millam, M. Klene, J. E. Knox, J. B. Cross, V. Bakken, C. Adamo, J. Jaramillo, R. Gomperts, R. E. Stratmann, O. Yazyev, A. J. Austin, R. Cammi, C. Pomelli, J. W. Ochterski, R. L. Martin, K. Morokuma, V. G. Zakrzewski, G. A. Voth, P. Salvador, J. J Dannenberg, S. Dapprich, A. D. Daniels, O. Farkas, J. B. Foresman, J. V. Ortiz, J. Cioslowski, and D. J. Fox, Gaussian, Inc., Wallingford CT, 2013.

2 G. M. J. Barca, C. Bertoni, L. Carrington, D. Datta, N. De Silva, J. E. Deustua, D. G. Fedorov, J. R. Gour, A. O. Gunina, E. Guidez, T. Harville, S. Irle, J. Ivanic, K. Kowalski, S. S. Leang, H. Li, W. L., J. J. Lutz, I. Magoulas, J. Mato, V. Mironov, H. Nakata, B. Q. Pham, P. Piecuch, D. Poole, S. R. Pruitt, A. P. Rendell, L. B. Roskop, K. Ruedenberg, T. Sattasathuchana, M. W. Schmidt, J. Shen, L. Slipchenko, M. Sosonkina, V. Sundriyal, A. Tiwari, J. L. Galvez Vallejo, B. Westheimer, M. Wloch, P. Xu, F. Zahariev and M. S. Gordon. *J. Chem. Phys*. 2020, **152**, 154102.

3 F. Neese, *WIRES Comput. Molec. Sci.*, 2022, **12**, e1606.

4 I. Schapiro, K. Sivalingam and F. Neese, *J. Theo. Comp. Chem.*, 2013, **9**, 3567-3580.

5 C. Kollmar, K. Sivalingam and F. Neese, *J. Chem. Phys.*, 2021, **155**, 234104.
